# COVAC1 phase 2a expanded safety and immunogenicity study of a self-amplifying RNA vaccine against SARS-CoV-2

**DOI:** 10.1016/j.eclinm.2022.101823

**Published:** 2023-01-13

**Authors:** Alex J. Szubert, Katrina M. Pollock, Hannah M. Cheeseman, Jasmini Alagaratnam, Henry Bern, Olivia Bird, Marta Boffito, Ruth Byrne, Tom Cole, Catherine A. Cosgrove, Saul N. Faust, Sarah Fidler, Eva Galiza, Hana Hassanin, Mohini Kalyan, Vincenzo Libri, Leon R. McFarlane, Ana Milinkovic, Jessica O'Hara, David R. Owen, Daniel Owens, Mihaela Pacurar, Tommy Rampling, Simon Skene, Alan Winston, James Woolley, Yee Ting N. Yim, David T. Dunn, Sheena McCormack, Robin J. Shattock, COVAC 1 Study Team

**Affiliations:** a cMRC Clinical Trials Unit at UCL, London, UK; b Department of Infectious Disease, Imperial College London, UK; c St George's Vaccine Institute, Institute for Infection and Immunity, St George's University of London, UK; d Chelsea & Westminster Hospital, London, UK; e NIHR Southampton Clinical Research Facility and Biomedical Research Centre, University Hospital Southampton NHS Foundation Trust, Southampton, UK; f Faculty of Medicine and Institute for Life Sciences, University of Southampton, Southampton, UK; g Surrey Clinical Research Facility, Faculty of Health and Medical Sciences, University of Surrey, Guildford, UK; h dNIHR UCLH Clinical Research Facility and NIHR UCLH Biomedical Research Centre, London, UK; i NIHR Imperial Clinical Research Facility and NIHR Imperial Biomedical Research Centre, London, UK; j Department of Brain Sciences, Imperial College London, London, UK

**Keywords:** Self-amplifying RNA, Vaccine, SARS-CoV-2, Clinical trial, Safety, Immunogenicity

## Abstract

**Background:**

Lipid nanoparticle (LNP) encapsulated self-amplifying RNA (saRNA) is well tolerated and immunogenic in SARS-CoV-2 seronegative and seropositive individuals aged 18–75.

**Methods:**

A phase 2a expanded safety and immunogenicity study of a saRNA SARS-CoV-2 vaccine candidate LNP-nCoVsaRNA, was conducted at participating centres in the UK between 10th August 2020 and 30th July 2021. Participants received 1 μg then 10 μg of LNP-nCoVsaRNA, ∼14 weeks apart. Solicited adverse events (AEs) were collected for one week post-each vaccine, and unsolicited AEs throughout. Binding and neutralisating anti-SARS-CoV-2 antibody raised in participant sera was measured by means of an anti-Spike (S) IgG ELISA, and SARS-CoV-2 pseudoneutralisation assay. (The trial is registered: ISRCTN17072692, EudraCT 2020-001646-20).

**Findings:**

216 healthy individuals (median age 51 years) received 1.0 μg followed by 10.0 μg of the vaccine. 28/216 participants were either known to have previous SARS-CoV2 infection and/or were positive for anti-Spike (S) IgG at baseline. Reactogenicity was as expected based on the reactions following licensed COVID-19 vaccines, and there were no serious AEs related to vaccination. 80% of baseline SARS-CoV-2 naïve individuals (147/183) seroconverted two weeks post second immunization, irrespective of age (18–75); 56% (102/183) had detectable neutralising antibodies. Almost all (28/31) SARS-CoV-2 positive individuals had increased S IgG binding antibodies following their first 1.0 μg dose with a ≥0.5log10 increase in 71% (22/31).

**Interpretation:**

Encapsulated saRNA was well tolerated and immunogenic in adults aged 18–75 years. Seroconversion rates in antigen naïve were higher than those reported in our dose-ranging study. Further work is required to determine if this difference is related to a longer dosing interval (14 vs. 4 weeks) or dosing with 1.0 μg followed by 10.0 μg. Boosting of S IgG antibodies was observed with a single 1.0 μg injection in those with pre-existing immune responses.

**Funding:**

Grants and gifts from the 10.13039/501100000265Medical Research Council UKRI (MC_PC_19076), the 10.13039/100000002National Institute for Health Research/Vaccine Task Force, Partners of Citadel and Citadel Securities, Sir Joseph Hotung Charitable Settlement, 10.13039/501100023262Jon Moulton Charity Trust, Pierre Andurand, and Restore the Earth.

**Research in context**

**Evidence before this study**

We previously reported the first published phase I dose ranging study of an LNP encapsulated saRNA vaccine (LNP-nCoVsaRNA). Using the search terms “self-amplifying RNA” AND “clinical trial”, no additional clinical studies have been published between October 9 2021 and 1 June 2022.

**Added value of this study**

The SARS-CoV-2 saRNA vaccine (LNP-nCoVsaRNA) given at 1 μg and 10 μg doses 14 weeks apart was well tolerated in adults aged 18–75 years, with fewer adverse reactions with increasing age. Seroconversion rates by ELISA in SARS-CoV-2 naïve individuals; 80% (147/183), were higher than those previously reported following two 1 μg 43% (18/42), or 10 μg 61% (14/23) doses given 4 weeks apart, and binding titres were 5- and 2.5- fold higher respectively. Anti-S IgG responses in 90% (28/31) of participants who were seropositive for SARS-CoV-2 at baseline were boosted following a single vaccination with 1 μg of LNP-nCoVsaRNA.

**Implications of all the available evidence**

Seroconversion rates were significantly higher than those we previously reported. Further work will determine if this is related to a longer interval (14 vs. 4 weeks) or dosing with 1 μg followed by 10 μg. The response to a single 1 μg dose in SARS-CoV-2 seropositive individuals offers promise that saRNA may provide a low dose and low-cost booster vaccine for long-term management of COVID-19.

## Introduction

Lipid nanoparticle (LNP) encapsulated self-amplifying RNA (saRNA) is a novel technology for vaccine development with the potential to be immunogenic at low dose levels.^1^ The platform employs a synthetic RNA molecule which includes the antigen of interest, in this case the stabilised Spike (S) glycoprotein from SARS-CoV-2, in combination with the non-structural amplicon derived from an alphavirus, Venezuelan equine encephalitis virus.^2^ We reported data from the dose-ranging cohort component of COVAC1 (ISRCTN17072692, EudraCT 2020-001646-20) in 2021, the first published study of a LNP encapsulated saRNA vaccine.^3^ The saRNA vaccine administered in two intramuscular doses 4 weeks apart was well-tolerated but failed to induce seroconversion in 100% of participants.^3^

To respond to the need for COVID-19 vaccine development and prepare for transition to efficacy testing, Phase 2a of COVAC1 was initiated in parallel with the dose finding study. The Phase 2a trial enrolled a larger population to obtain a more precise estimate of safety and immunogenicity following administration of the highest dose levels studied in the dose-ranging cohort. This expanded safety cohort included individuals with a wider age range, stable co-morbidities, and a sub-set with a prior history of laboratory confirmed SARS-CoV-2 infection.

There was uncertainty about the optimum dose level of encapsulated saRNA required for IM injection at the start of COVAC1 as the technology was novel in humans and there were no clinical data to guide dose level selection. Pre-clinical data from small animal models supported the likelihood that the ideal dose would lie between 0.1 and 1 μg, but 1 μg proved insufficient to induce 100% seroconversion in the dose escalation phase.^3^ COVAC1 was therefore adapted to evaluate dose levels up to 10 μg. The expanded safety cohort received an initial prime with a 1 μg dose but were invited to delay their second vaccine to receive the highest tolerated dose (10 μg). Here we report the results from the expanded safety cohort who received 1 μg following by 10 μg of the candidate saRNA COVID-19 vaccine, LNP-nCoVsaRNA, approximately 14 weeks apart.

## Methods

### Study design and participants

We report the results of the open label expanded safety component of the protocol (see protocol version 8.0, Appendix 1). Healthy participants aged 18–75 years were recruited through local advertisements. Participants with no history of COVID-19 were eligible to take part, but participant sera were not prospectively screened for antibodies against SARS-CoV-2, except for the St Mary's Hospital site (which preferentially enrolled participants prospectively known to have infection-induced SARS-CoV-2 antibodies prior to enrolment). All participants underwent a screening visit where a full medical history and examination was performed in addition to blood and urine tests. Participant sera were screened for the presence of blood borne viruses using a fourth generation HIV test and for IgG against Hepatitis C. Those with reactive responses in either of these tests were ineligible for the study. Full details of the eligibility criteria are described in Appendix 2.

Written informed consent was obtained from all participants, and the trial conducted in accordance with the principles of the Declaration of Helsinki and Good Clinical Practice. Participants were offered reimbursement for their time, inconvenience, and travel expenses of £50 per visit paid as a lump sum at the end of participation. This study was approved in the UK by the Medicines and Healthcare products Regulatory Agency and the North East–York Research Ethics Committee (reference 20/SC/0145) (ISRCTN17072692, EudraCT 2020-001646-20).

### Procedures

LNP-nCoVsaRNA is a self-amplifying ribonucleic acid (saRNA) vaccine, encapsulated within lipid nanoparticles (LNPs). It encodes two major components; the non-structural replicase proteins from VEEV and the spike (S) glycoprotein of SARS-CoV-2 stabilised in the prefusion conformation with two proline substitutions.^2^

#### Conduct of the trial

The expanded safety cohort component of COVAC1 evaluated LNP-nCoVsaRNA as two intramuscular (IM) injections into the deltoid muscle of the non-dominant arm. The first injection was of 1.0 μg; the second, given 14 weeks later, was 10.0 μg (one participant chose to and received two injections of 1.0 μg, four weeks apart). The vaccine was formulated as a suspension for injection in multi-dose vials stored at −70 °C. On the day of injection, it was thawed and diluted in phosphate buffered saline (PBS) to give a final volume of 0.5 mL for injection. Stability of both frozen and diluted product was supported by an extensive stability database as required by the regulators. Participants were observed for up to 1 h following each injection.

Solicited adverse events (AEs) were self-reported by participants in electronic diary records captured the evening after injection and for six further evenings. Study staff checked the diary record approximately 48 h post-injection and at the day seven visit. All these events were considered related to vaccination. AEs, including any following receipt of an authorised/licensed COVID-19 vaccine as part of NHS roll-out in England, were captured by study staff at every visit. Causality was determined by the site investigators. Blood samples (haemoglobin, white cell count, platelets, lymphocytes, neutrophils, ALT/AST, alkaline phosphatase, total bilirubin, serum creatinine and non-fasting glucose) were collected at 1, 2 and 4 weeks after the first vaccine; on the day of the second vaccine (pre-vaccination); 1, 2, 4 and 8 weeks after the second vaccine; and at 52 weeks. Grade was determined according to the FDA toxicity table for healthy volunteers, adapted to site laboratory normal reference ranges (see Appendix 2).

#### Immunological assessments

Binding antibody concentrations were assessed using a sensitive anti-S IgG ELISA as previously described.^3^ For individuals who tested positive for anti-S IgG antibodies at baseline, subsequent visits were tested for binding antibody using an in-house conventional ELISA platform to avoid the need for large dilutions. In brief, 96-well high-binding plates (Greiner, Kremsmünster, Austria) were coated with anti-human kappa and lambda light chain specific mouse antibodies (Southern Biotech, Birmingham, AL) at 1:1 ratio diluted 1:500 in PBS or antigen (1 μg/mL recombinant SARS-CoV-2 spike protein for 1 h at 37 °C. The rest of the protocol is as described in^4^. Irrespective of the ELISA platform used, the first WHO international standard anti-SARS-CoV-2 immunoglobulin was added at a concentration of 2 BAU/mL, equivalent of approximately 20,000 ng/mL, as a control. SARS-CoV-2 neutralisation assays were conducted using pseudotyped (PSV) viruses as described.^3^ The first WHO International Standard for anti-SARS-CoV-2 immunoglobulin included as a positive control, determined to have an IC50 neutralisation titre of approximately 1:3000.

### Outcomes

The safety outcome measures were solicited local injection site and systemic reactions that started within seven days of administration of the vaccine, and any of the following that occurred throughout the study period: unsolicited adverse reactions (ARs), serious adverse events (SAEs) and unsolicited AEs. Immunogenicity was assessed by the titre of neutralising antibodies at baseline, two and four weeks after the second injection, and the titre of IgG raised against the SARS-CoV-2 S glycoprotein two weeks after the first injection, on the day of the second injection (pre-injection) and two and four weeks after the second injection.

### Statistical analysis

Sample size was based on achieving adequate statistical power to detect an adverse reaction with a true frequency of between 1/100 and 1/50 or higher. To assess the safety of the vaccine candidate in people with pre-existing immunity to SARS-CoV-2, a group was enrolled that had known anti-S IgG-in serum. 31 participants were prospectively known to have infection-induced SARS-CoV-2 antibodies prior to enrolment, or anti-Spike IgG detectable retrospectively at baseline prior to vaccination. Data from these participants are included in safety analyses but have undergone separate immune analysis. For participants who acquired SARS-CoV-2 infection (laboratory-confirmed) during follow-up or who received an authorised/licensed COVID-19 vaccine, subsequent immunogenicity data are excluded. Further exclusions from the immunogenicity analyses include the participant that received two injections of 1.0 μg, and the week 2 and week 4 post-second vaccine samples for the five participants who only received their first injection. Binding and neutralisation antibody titres were analysed on a logarithmic scale and back transformed for presentation. All analyses were carried out using Stata 16.0 (StataCorp, College Station, TX, USA).

### Role of the funding source

The funders had no role in the study design, the collection, analysis, and interpretation of data, writing of the report and the decision to submit for publication.

Trial statisticians AS, HB and DTD had access to and verified all raw data sets. AS, KMP, HB, HMC, DTD, SM and RJS made the decision to submit the manuscript.

## Results

### Recruitment and compliance

**Fig. 1:**
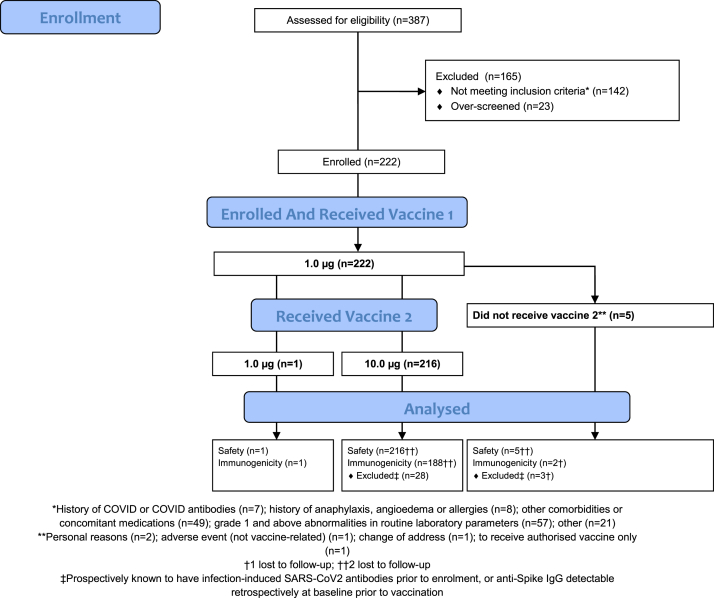
Consort diagram demonstrating the eligibility assessment, enrolment, group allocation and follow-up of the n = 222 participants in the phase 2a expanded safety and immunogenicity study.

A total of 222 participants were enrolled between 10 August and 20 August 2020 (Fig. 1). Approximately half were male (114/222; 51%) with median age 51 years (Table 1). Twelve participants were known to have had laboratory-confirmed symptomatic SARS-CoV-2 infection prior to enrolment (all had detectable anti-Spike IgG at baseline); in an additional 19 participants, anti-Spike IgG was detected retrospectively at baseline prior to vaccination (indicative of an asymptomatic infection). 216 (97%) participants received 1.0 μg followed by 10.0 μg (median 14.1 weeks apart (inter-quartile range (IQR) 14.0, 14.7); range 13.0–21.9); one participant had 1.0 μg followed by 1.0 μg (4.0 weeks apart); and five participants received only a single injection (1.0 μg). Only one participant was out of the window period for the second injection. By the end of follow-up (52 weeks), 219/222 (99%) were known to have received an authorised/licensed COVID-19 vaccine (median 28.0 weeks post-enrolment (IQR 24.4, 36.6); range 18.0–45.4) (see Appendix 2, Table 5). Overall, 92% (2451/2663) of visits were carried out in the protocol window, including 91% (202/222) of vaccine 2 + 2 weeks and 90% (199/222) of vaccine 2 + 4 weeks visits (or, for participants who did not receive vaccine 2, the corresponding visits).

**Table 1:** Demographics of participants enrolled.

	Total N = 222
**Sex**	
Male	114 (51.4%)
Female	108 (48.6%)
**Age at last birthday (years)**	
Median (IQR)	51 (31, 62)
Range	20–73
**Ethnicity**	
White	198 (89.2%)
Mixed	5 (2.3%)
Asian or Asian British	8 (3.6%)
Black or Black British	3 (1.4%)
Other	6 (3.6%)
**BMI**	
Mean (SD)	25.3 (4.03)
Median (IQR)	24.7 (22.2, 27.5)
Range	18.3–40.4
**History of SARS-CoV-2 infection**^a^	
No	191 (86.0%)
Yes	31 (14.0%)
**Centre**	
Chelsea and Westminster	29 (13.1%)
St Georges	37 (16.7%)
St Marys	37 (16.7%)
University Hospital Southampton	40 (18.0%)
Surrey CRF	38 (17.1%)
UCLH	41 (18.5%)

a Prospectively known to have infection-induced SARS-CoV-2 antibodies prior to enrolment; or anti-Spike IgG detectable retrospectively at baseline prior to vaccination.

#### Reactogenicity

Considering the first vaccine (1.0 μg), the proportion of participants reporting a local reaction was 53% (117/222). Common reactions reported were tenderness/discomfort (109/222; 49%) and pain (45; 20%), whilst erythema (6; 3%) and swelling (1; <1%) were uncommon (Table 2 and Table 3). No participant reported a grade 3 (severe) local reaction. The proportion of participants reporting a systemic reaction was 58% (128/222). Common reactions were fatigue (65/222; 29%) and headache (65; 29%). Seven days after vaccination, laboratory safety parameters remained largely within normal limits (Appendix 2 Table 6.5).

**Table 2:** Selected local injection site and systemic clinical reactions starting within 7 days of the first vaccine by age at enrolment for participants who received 1.0 μg (dose-ranging cohort/expanded safety cohort).

	18–39 N = 117	40–59 N = 69	60–75 N = 78	Total N = 264	p-value
**Pain**					**0.0001**
Normal	79 (67.5%)	56 (81.2%)	71 (91.0%)	206 (78.0%)	
Grade 1	33 (28.2%)	13 (18.8%)	7 (9.0%)	53 (20.1%)	
Grade 2	5 (4.3%)	0 (0.0%)	0 (0.0%)	5 (1.9%)	
**Tenderness/discomfort**					**<0.0001**
Normal	38 (32.5%)	36 (52.2%)	50 (64.1%)	124 (47.0%)	
Grade 1	73 (62.4%)	33 (47.8%)	28 (35.9%)	134 (50.8%)	
Grade 2	6 (5.1%)	0 (0.0%)	0 (0.0%)	6 (2.3%)	
**Chills/shivering**					**0.84**
Normal	114 (97.4%)	67 (97.1%)	77 (98.7%)	258 (97.7%)	
Grade 1	3 (2.6%)	1 (1.4%)	0 (0.0%)	4 (1.5%)	
Grade 2	0 (0.0%)	1 (1.4%)	1 (1.3%)	2 (0.8%)	
**Myalgia (flu-like general muscle aches)**					**0.12**
Normal	103 (88.0%)	65 (94.2%)	74 (94.9%)	242 (91.7%)	
Grade 1	10 (8.5%)	4 (5.8%)	3 (3.8%)	17 (6.4%)	
Grade 2	4 (3.4%)	0 (0.0%)	1 (1.3%)	5 (1.9%)	
**Arthralgia**					**0.44**
Normal	108 (92.3%)	66 (95.7%)	71 (91.0%)	245 (92.8%)	
Grade 1	9 (7.7%)	3 (4.3%)	6 (7.7%)	18 (6.8%)	
Grade 2	0 (0.0%)	0 (0.0%)	1 (1.3%)	1 (0.4%)	
**Fatigue**					**0.0008**
Normal	71 (60.7%)	54 (78.3%)	64 (82.1%)	189 (71.6%)	
Grade 1	34 (29.1%)	13 (18.8%)	12 (15.4%)	59 (22.3%)	
Grade 2	12 (10.3%)	2 (2.9%)	2 (2.6%)	16 (6.1%)	
**Headache**					**0.07**
Normal	78 (66.7%)	46 (66.7%)	61 (78.2%)	185 (70.1%)	
Grade 1	35 (29.9%)	18 (26.1%)	17 (21.8%)	70 (26.5%)	
Grade 2	4 (3.4%)	5 (7.2%)	0 (0.0%)	9 (3.4%)	

Analysis of variance (ANOVA) for grade (including, “normal,” as grade 0) used to test for difference between age groups *(global test across age groups).*

**Table 3:** Selected local injection site and systemic clinical reactions starting within 7 days of the second vaccine by age at enrolment for participants who received 1.0 μg followed by 10.0 μg (expanded safety cohort).

	18–39 N = 81	40–59 N = 58	60–75 N = 77	Total N = 216	p-value
**Pain**					**0.0022**
Normal	15 (18.5%)	21 (36.2%)	32 (41.6%)	68 (31.5%)	
Grade 1	44 (54.3%)	27 (46.6%)	36 (46.8%)	107 (49.5%)	
Grade 2	22 (27.2%)	9 (15.5%)	9 (11.7%)	40 (18.5%)	
Grade 3	0 (0.0%)	1 (1.7%)	0 (0.0%)	1 (0.5%)	
**Tenderness/discomfort**					**0.006**
Normal	4 (4.9%)	8 (13.8%)	10 (13.0%)	22 (10.2%)	
Grade 1	41 (50.6%)	39 (67.2%)	49 (63.6%)	129 (59.7%)	
Grade 2	35 (43.2%)	11 (19.0%)	18 (23.4%)	64 (29.6%)	
Grade 3	1 (1.2%)	0 (0.0%)	0 (0.0%)	1 (0.5%)	
**Chills/shivering**					**0.0002**
Normal	18 (22.2%)	28 (48.3%)	40 (51.9%)	86 (39.8%)	
Grade 1	22 (27.2%)	15 (25.9%)	14 (18.2%)	51 (23.6%)	
Grade 2	38 (46.9%)	12 (20.7%)	23 (29.9%)	73 (33.8%)	
Grade 3	3 (3.7%)	3 (5.2%)	0 (0.0%)	6 (2.8%)	
**Myalgia (flu-like general muscle aches)**					**<0.0001**
Normal	16 (19.8%)	26 (44.8%)	39 (50.6%)	81 (37.5%)	
Grade 1	25 (30.9%)	16 (27.6%)	23 (29.9%)	64 (29.6%)	
Grade 2	37 (45.7%)	13 (22.4%)	15 (19.5%)	65 (30.1%)	
Grade 3	3 (3.7%)	3 (5.2%)	0 (0.0%)	6 (2.8%)	
**Arthralgia**					**0.09**
Normal	43 (53.1%)	42 (72.4%)	44 (57.1%)	129 (59.7%)	
Grade 1	19 (23.5%)	8 (13.8%)	25 (32.5%)	52 (24.1%)	
Grade 2	18 (22.2%)	7 (12.1%)	8 (10.4%)	33 (15.3%)	
Grade 3	1 (1.2%)	1 (1.7%)	0 (0.0%)	2 (0.9%)	
**Fatigue**					**0.06**
Normal	14 (17.3%)	18 (31.0%)	25 (32.5%)	57 (26.4%)	
Grade 1	22 (27.2%)	21 (36.2%)	29 (37.7%)	72 (33.3%)	
Grade 2	40 (49.4%)	14 (24.1%)	20 (26.0%)	74 (34.3%)	
Grade 3	5 (6.2%)	5 (8.6%)	3 (3.9%)	13 (6.0%)	
**Headache**					**0.0002**
Normal	12 (14.8%)	26 (44.8%)	34 (44.2%)	72 (33.3%)	
Grade 1	32 (39.5%)	18 (31.0%)	18 (23.4%)	68 (31.5%)	
Grade 2	32 (39.5%)	13 (22.4%)	23 (29.9%)	68 (31.5%)	
Grade 3	5 (6.2%)	1 (1.7%)	2 (2.6%)	8 (3.7%)	

Analysis of variance (ANOVA) for grade (including, “normal,” as grade 0) used to test for difference between age groups *(global test across age groups).*

Following the second vaccination, the proportion of participants receiving 10.0 μg reporting a local reaction (202/216; 94%) was much higher than when receiving 1 μg. Common reactions reported were tenderness/discomfort (194/216; 90%) and pain (148; 69%) whilst swelling (5; 2%) and erythema (4; 2%) remained uncommon (Table 2 and Table 3). Two participants reported a grade 3 local reaction (tenderness/discomfort, or pain). The proportion of participants reporting a systemic reaction was 88% (191/216). Common reactions were fatigue (159/216; 74%), headache (144; 67%), myalgia (135; 63%), chills/shivering (130; 60%), arthralgia (87; 40%), nausea (59; 27%) and fever (≥38 °C) (28; 13%). 24 participants (11%) reported a grade 3 systemic reaction. Seven days after vaccination, laboratory safety parameters remained largely within normal limits except for neutrophils: 11% (23/213) had neutropaenia that was not considered clinically significant (all 1.0–1.9 × 10^9^/L) (Appendix 2 Table 6.6).

The frequency of adverse reactions was age-dependent for certain outcomes, becoming less frequent at older ages (Table 2 and Table 3 and Appendix 2, Tables 6.7–10). The effect was evident, after both first and second vaccinations, for pain, tenderness, and fatigue. An association after the second vaccination was also observed for headache, myalgia and chills/shivering.

Local and systemic reactions were generally similar in those with and without a history of COVID-19 (Appendix 2, Table 6.11). Nausea appeared more frequent after the second injection in those with a history of COVID-19 (57.1% vs. 22.9%) (Appendix 2, Table 6.14).

#### Other adverse events

There were no SAEs considered related to the study intervention. Amongst the 201 (91%) participants reporting 956 AEs, 89 had a moderately severe event (including one SAE (foot bunion requiring surgery)), and 12 had a severe or worse event (Appendix 2, Table 6.15). Four of the 12 were SAEs that required hospital admission (myocardial ischaemia, tibia fracture, benign parathyroid tumour, osteoarthritis). The other eight were COVID-19/bacterial pneumonia, syncope, urinary tract infection, tonsillitis (participant separately experienced grade 4 back pain for which they attended the Accident & Emergency department, subsequently), varicocele repair, headache; and two participants with grade 2 neutropaenia at enrolment prior to vaccine had grade 3 neutropenia 105 days after the first and 56 days after the second vaccine.

### Immunogenicity

#### Binding antibody

Binding antibody to anti-S IgG, as measured by ELISA, is reported for samples obtained at 2 weeks post-first vaccine, day of second vaccine (pre-injection), and 2 weeks and 4 weeks post-second vaccine (Table 4, Table 5). In addition to the expanded safety cohort (1.0 μg followed by 10.0 μg group), the 1.0 μg group and 10.0 μg group from the dose-ranging cohort^3^ are included for comparison. It is important to note the comparison between dose levels is confounded with the dosing schedule; the gap between first and second vaccination was much shorter for the 1.0 μg and 10.0 μg dose-ranging groups (median 4 weeks) than for the expanded safety group (median 14.1 weeks).

**Table 4:** Seroconversion rates, anti-S IgG concentrations, and neutralising antibody titres.

		Dose			p-value
		1.0 μg *N* = *42*	10.0 μg *N* = *23*	1.0 μg followed by 10.0 μg *N* = *188*	
**Binding antibody by ELISA (ng/ml)**					
Week 2 post-vaccine 1	Seroconversion, n (%)	3 (7%)	0 (0%)	2 (1%)	–
	GM titre (95% CI)	77 (47–124)	–	655 (20–21469)	–
Vaccine 2	Seroconversion, n (%)	8 (19%)	8 (35%)	17 (9%)	–
	GM titre (95% CI)	164 (83–325)	258 (139–479)	470 (266–830)	–
Week 2 post-vaccine 2	Seroconversion, n (%)	18 (43%)	14 (61%)	147 (80%)	0.057
	GM titre (95% CI)	251 (184–343)	500 (233–1076)	1224 (1008–1486)	**0.010**
Week 4 post-vaccine 2	Seroconversion, n (%)	20 (48%)	13 (57%)	143 (80%)	**0.016**
	GM titre (95% CI)	262 (186–367)	508 (199–1294)	1294 (1065–1573)	**0.010**
**Neutralising antibody (NT**_**50**_**)**					
Week 2 post-vaccine 2	Seroconversion, n (%)	14 (33%)	10 (43%)	102 (56%)	0.277
	GM dilution (95% CI)	46 (31–70)	70 (27–180)	68 (53–86)	0.928
Week 4 post-vaccine 2	Seroconversion, n (%)	7 (17%)	12 (52%)	99 (56%)	0.825
	GM dilution (95% CI)	30 (11–83)	124 (50–306)	75 (56–102)	0.297

1.0 μg and 10.0 μg doses assessed in dose-ranging cohort; 1.0 μg followed by 10.0 μg dose assessed in expanded safety cohort. GM, geometric mean. Calculated among seroconversion samples. Fisher's exact and t-tests used to compare seroconversion rates and geometric means among responders, respectively. P-values detail comparison between 10.0 μg and 1.0 μg followed by 10.0 μg groups. Missing values for binding antibody: 1.0 μg followed by 10.0 μg (week 2 post-vaccine 1, n = 3). Removed samples for binding antibody: 1.0 μg followed by 10.0 μg (vaccine 2, COVID infection n = 2). Missing values for binding and neutralising antibody: 1.0 μg followed by 10.0 μg (week 2 post-vaccine 2, n = 2; week 4 post-vaccine 2, n = 5). Removed samples for binding and neutralising antibody: 1.0 μg followed by 10.0 μg (week 2 post-vaccine 2, COVID infection n = 3; week 4 post-vaccine 2, COVID infection n = 3, authorised vaccine n = 2). Note: Among baseline convalescent samples GM binding titre (95% CI) was 650 (457–925) and GM NT 50 (95% CI) was 85 (56–129). Significant value ≤0.05 is shown in bold.

**Table 5:** Seroconversion rates, anti-S IgG concentrations, and neutralising antibody titres.

		Age group			p-value
		18–39 *N* = *64*	40–59 *N* = *50*	60–75 *N* = *74*	
**Binding antibody by ELISA (ng/ml)**					
Week 2 post-vaccine 1	Seroconversion, n (%)	2 (3%)	0 (0%)	0 (0%)	0.180
	GM titre (95% CI)	655 (20–21469)	–	–	–
Vaccine 2	Seroconversion, n (%)	11 (18%)	3 (6%)	3 (4%)	**0.021**
	GM titre (95% CI)	342 (163–719)	724 (223–2355)	983 (363–2663)	0.335
Week 2 post-vaccine 2	Seroconversion, n (%)	52 (85%)	36 (73%)	59 (81%)	0.333
	GM titre (95% CI)	1473 (1125–1929)	1185 (768–1829)	1060 (764–1472)	0.352
Week 4 post-vaccine 2	Seroconversion, n (%)	51 (88%)	35 (74%)	57 (78%)	0.171
	GM titre (95% CI)	1159 (844–1590)	1132 (730–1754)	1552 (1156–2084)	0.333
**Neutralising antibody (NT**_**50**_**)**					
Week 2 post-vaccine 2	Seroconversion, n (%)	36 (59%)	28 (57%)	38 (52%)	0.717
	GM dilution (95% CI)	69 (46–102)	65 (40–105)	69 (47–102)	0.979
Week 4 post-vaccine 2	Seroconversion, n (%)	39 (67%)	22 (47%)	38 (52%)	0.080
	GM dilution (95% CI)	49 (32–75)	124 (61–249)	88 (53–144)	0.059

GM, geometric mean. Calculated among seroconversion samples. Fisher's exact and one-way analysis of variance tests used to compare seroconversion rates and geometric means among responders, respectively. Missing values for binding antibody: 18–45 (week 2 post-vaccine 1, n = 2), 46–59 (week 2 post-vaccine 1, n = 1). Removed samples for binding antibody: 18–45 (vaccine 2, COVID infection n = 2). Missing values for binding and neutralising antibody: 18–45 (week 4 post-vaccine 2, n = 2), 46–59 (week 2 post-vaccine 2, n = 1; week 4 post-vaccine 2, n = 2), 60–75 (week 2 post-vaccine 2, n = 1; week 4 post-vaccine 2, n = 1). Removed samples for binding and neutralising antibody: 18–45 (week 2 post-vaccine 2, COVID infection n = 3; week 4 post-vaccine 2, COVID infection n = 3, authorised vaccine n = 1), 46–59 (week 4 post-vaccine 2, authorised vaccine n = 1). Significant value ≤0.05 is shown in bold.

**Fig. 2:**
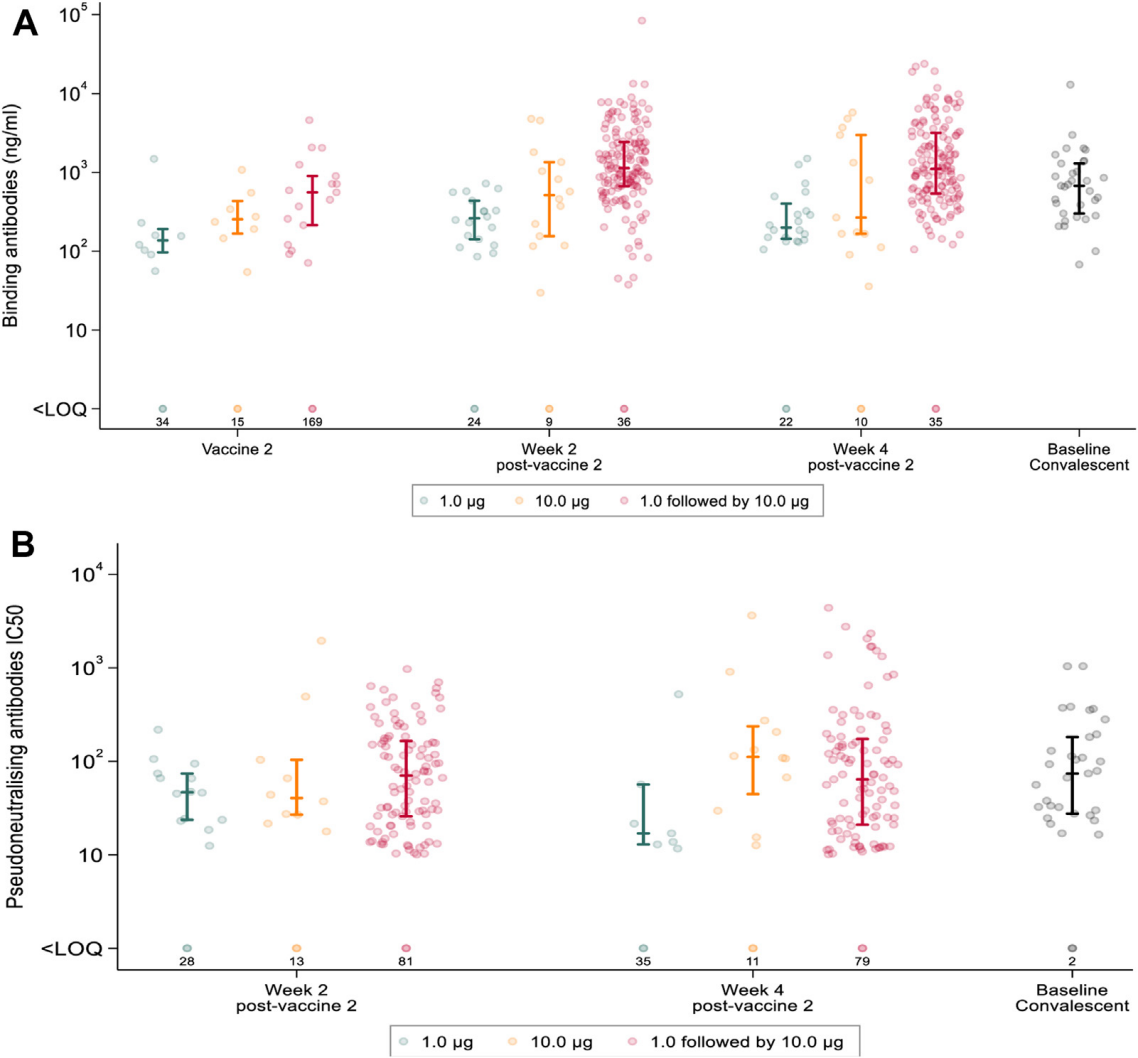
**A***.* Anti-Spike (S) IgG (ng/mL) responses raised in sera from participants receiving two doses of LNP-saRNA in the dose-ranging cohort (1 μg, 1 μg in green and 10 μg, 10 μg in orange) and the expanded safety cohort (1 μg, 10 μg in pink). Responses are shown on the day of second vaccination, and at 2 and 4 weeks after second vaccination. Baseline convalescent sera depicted in black. **B***.* Pseudoneutralising antibodies IC50 from participants receiving two doses of LNP-saRNA in the dose-ranging cohort (1 μg, 1 μg in green and 10 μg, 10 μg in orange) and the expanded safety cohort (1 μg, 10 μg in pink). Responses are shown at 2 and 4 weeks after second vaccination. Baseline convalescent sera depicted in black. Error bars detail the median and interquartile range amongst responders. Responses that did not meet criteria for a positive response are shown on the bottom row with numbers of participants <LOQ (limit of quantification).

Seroconversion to anti-S IgG by ELISA following second vaccination was higher in the expanded safety cohort (receiving 1.0 μg then 10.0 μg) than either the 1.0 μg group (receiving 1.0 μg then 1.0 μg) or the 10.0 μg group (receiving 10.0 μg then 10.0 μg) from the dose-ranging cohort. At 2 weeks post-second vaccine, seroconversion rates were 80% (147/183), 43% (18/42), and 61% (14/23) within the respective groups, with a similar pattern observed at 4 weeks post-second vaccine (Table 4, Table 5). Among those that seroconverted, anti-S IgG titre was approximately 5-fold higher in the expanded safety cohort (GM = 1224 ng/mL) compared to the 1.0 mg dose-ranging group (GM = 251 ng/mL), and 2.5-fold higher compared to the 10.0 mg dose-ranging group (GM = 500 ng/mL). Although the age range was wider for the expanded cohort (18–75 years) than for the dose-ranging groups, these differences persisted in additional analyses which adjusted for age (Appendix 2, Table 7). Anti-S IgG titre amongst responders in the expanded safety cohort was 2-fold higher compared with values derived from convalescent sera (GM = 650 ng/mL) (Fig. 2A).

#### Neutralising antibody

Neutralising antibody, as determined by pseudovirus assay against Wildtype, is reported for samples obtained 2 weeks and 4 weeks post-second vaccine (Table 4, Table 5). Similarly, with the binding antibody results, the 1.0 μg group and 10.0 μg group from the dose-ranging cohort are included for comparison.

There was no evidence of a difference in neutralising response in the expanded safety cohort when compared to the 10.0 μg dose-ranging group either at 2 weeks (102/183 (56%) vs. 10/23 (43%); difference 12% (95% CI [−9%, 34%])) or 4 weeks post-second vaccine (99/178 (56%) vs. 12/23 (52%); difference 3% (95% CI [−18%, 25%])). The NT_50_ geometric mean was similar between these groups at 2 weeks post-second vaccine (NT_50_ 70 vs. 68) and higher in the 10.0 μg group than in the expanded safety cohort at 4 weeks post-second vaccine (NT_50_ 124 versus 75) but this difference was not statistically significant (p = 0.30). Where seroconversion occurred NT_50_ values following the booster dose were broadly consistent with values derived from baseline convalescent sera (Fig. 2B). Responses to Omicron BA.5 were considerably lower and less frequent than that seen with the vaccine matched strain (Supplementary Fig. S14)

**Fig. 3:**
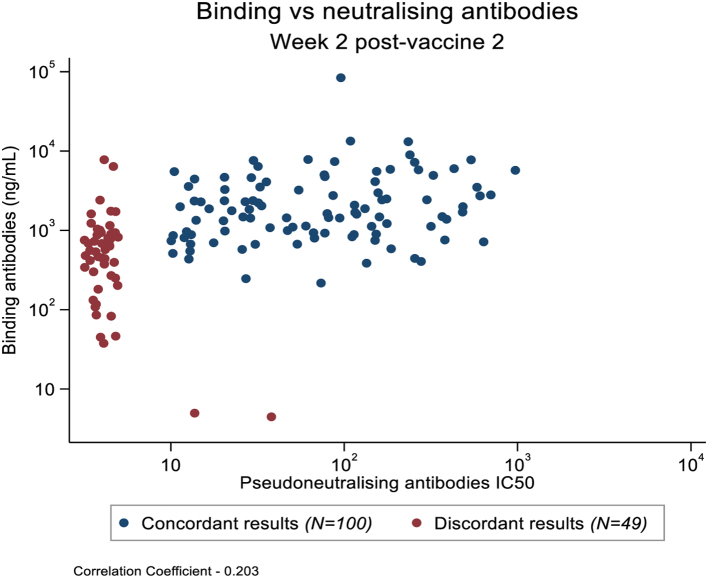
**Association between Pseudoneutralising antibodies (IC50) and anti-Spike (S) IgG.** Association between Pseudoneutralising antibodies (IC50) and anti-Spike (S) IgG (ng/mL) two weeks after second vaccination in the expanded safety cohort showing concordant (blue dots) and discordant (red dots) results.

Of the 147 participants who elicited an anti-S IgG binding response two weeks after the second vaccination, 47 (32%) had no measurable neutralising antibodies; another two participants showed the reverse pattern (Fig. 3). Among the participants with a response to both assays, only a modest correlation was observed between these two variables (r = 0.20). Similar findings were observed at four weeks after the second vaccination (data not shown).

**Fig. 4:**
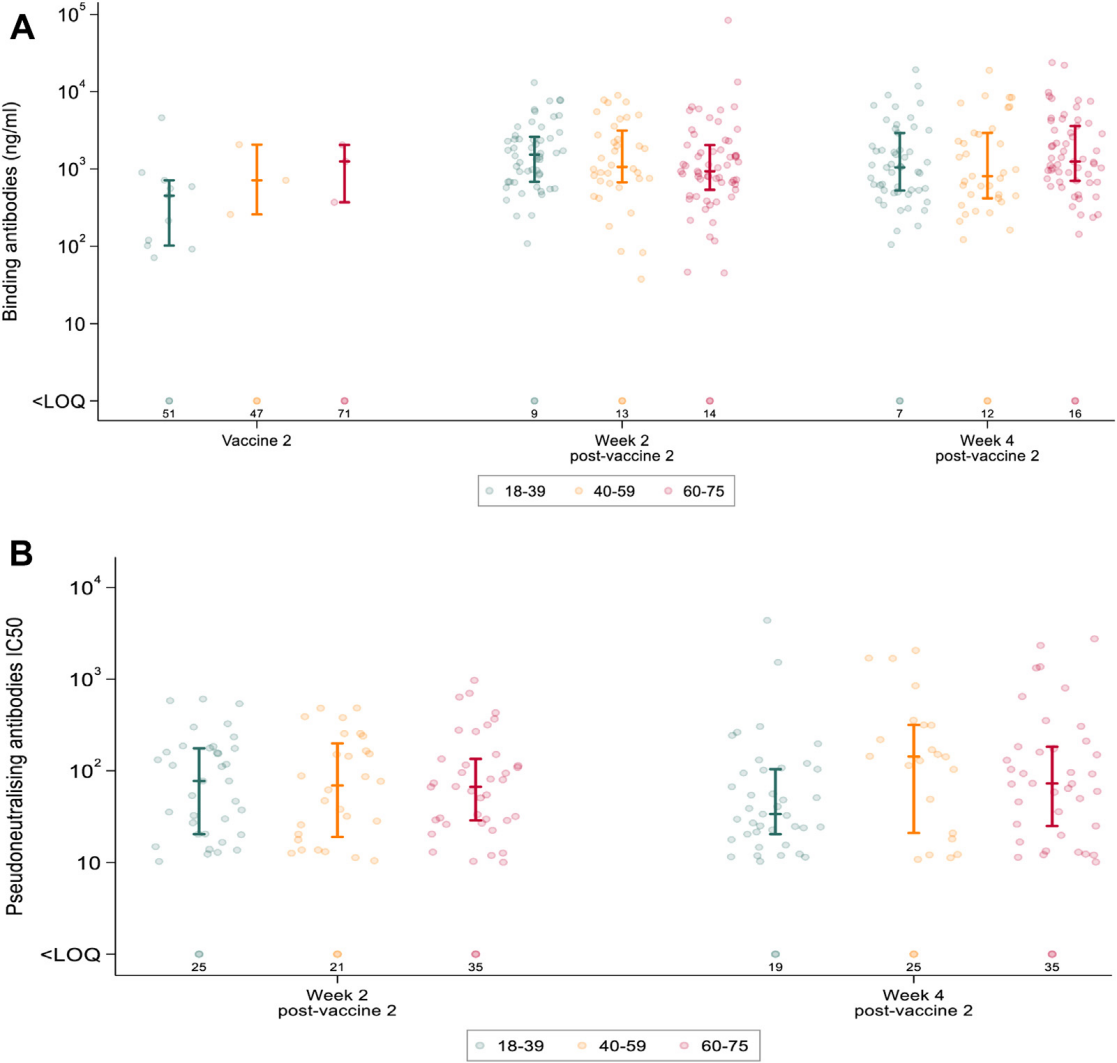
**A***.* Anti-Spike (S) IgG (ng/mL) responses raised in sera from participants receiving two doses of LNP-saRNA in the expanded safety cohort by age (18–39 green, 40–59 orange, 60–75 red). Responses are shown on the day of second vaccination, and at 2 and 4 weeks after second vaccination. **B***.* Pseudoneutralising antibodies IC50 from participants receiving two doses of LNP-saRNA responses in the expanded safety cohort by age (18–39 green, 40–59 orange, 60–75 red). Responses are shown 2 and 4 weeks after second vaccination. Error bars detail the median and interquartile range amongst responders. Responses that did not meet criteria for a positive response are shown on the bottom row with numbers of participants <LOQ (limit of quantification).

Binding and neutralising antibody responses were also analysed according to age group (18–39, 40–59 and 60–75). In contrast to the associations observed with reactogenicity, no significant differences were found in seroconversion rates nor the geometric means (anti-S IgG titre or NT_50_ for binding and neutralising assay respectively) across these age categories (Fig. 4A and B).

### Immune response in baseline seropositive participants

**Fig. 5:**
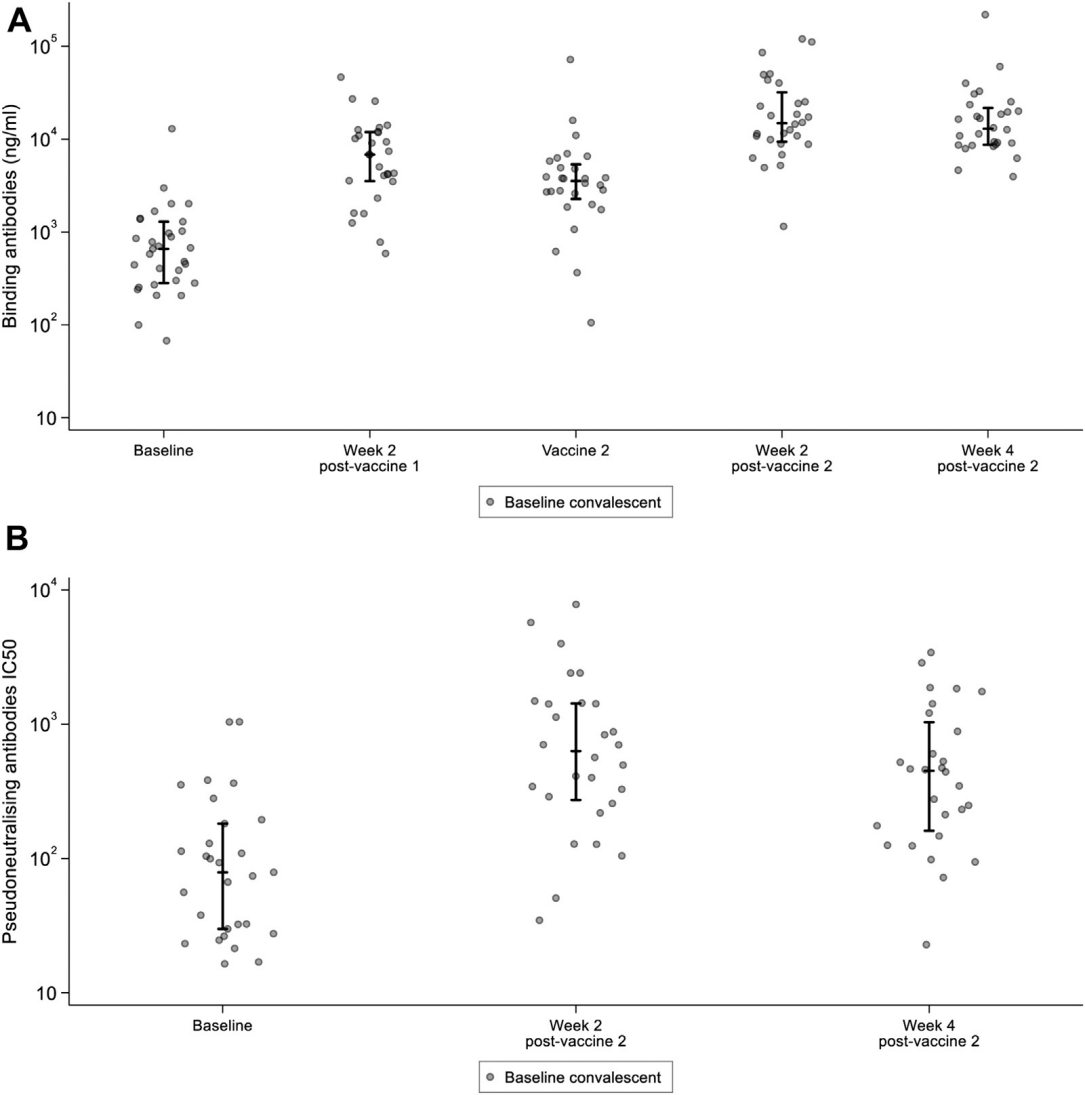
**A***.* Anti-Spike (S) IgG (ng/mL) responses raised in sera from seropositive participants receiving two doses of LNP-saRNA (1 μg at week 0 followed by 10 μg at week 12). Responses are shown at baseline (day of vaccine 1), 2 weeks post vaccine 1, on the day of second vaccination, and at 2 and 4 weeks after second vaccination. Error bars detail the median and interquartile range amongst responders. **B***.* Pseudoneutralising antibodies IC50 from seropositive participants receiving two doses of LNP-saRNA (1 μg at week 0 followed by 10 μg at week 12). Responses are shown at baseline (day of vaccine 1), and at 2 and 4 weeks after second vaccination. Error bars detail the median and interquartile range amongst responders.

As described above, 31 participants had detectable anti-spike IgG at baseline. Note that three participants did not receive their second vaccine and one participant had no immunology results after the second vaccination. In terms of binding antibody response, almost all participants showed an increase in serum concentration (28/31), with 71% (22/31) rising by 0.5 log_10_ or more following their first vaccination with 1 μg of saRNA (Fig. 5A). Binding antibody titres also increased in all participants after the second vaccine, 74% (20/27) rising by a further 0.5 log_10_ or more when receiving a 10 μg dose. Neutralising antibody responses, which were only measured after the second vaccination, showed a similar trend (Fig. 5B). Longitudinal immune responses for these participants are shown in Supplementary Fig. S15.

## Discussion

Lipid encapsulated saRNA is a novel vaccine platform technology where there is limited clinical experience. We previously reported the proof-of-concept and dose finding for this formulation in younger adults aged 18–45 years.^3^ Here we report data supporting its use in a wider cohort including older people, people with co-morbidities and with previous immunity to SARS-CoV-2, raising no safety concerns. Tolerability was dose related however, as the frequency and severity of adverse reactions was dose-dependent, as seen in the dose-ranging cohort, with more than 1 in 10 participants experiencing a severe reaction following the 10 μg dose. The frequency of adverse reactions was age-dependent with lower frequencies in older age groups for pain, tenderness, and fatigue after the first and second vaccines, and for headache, myalgia and chills/shivering after the second vaccine.

Participants in the expanded safety cohort were more likely to seroconvert than those in the 1 μg or 10 μg groups in the dose-ranging cohort. The magnitude of response was also significantly higher in the expanded safety cohort than the dose-ranging cohort, and 2-fold higher compared to convalescent sera. This finding is interesting, given that the total dose received by the safety cohort (1 μg then 10 μg) was 55% of the total dose given in the 10 μg group (10 μg then 10 μg). This could be explained by the longer interval (a median of 14 weeks compared to 4 weeks) between doses, a more efficient priming by the lower dose (1 μg vs. 10 μg), or larger number of subjects providing a more precise estimate. Indeed, a longer dosing interval has been associated with efficacy of an adenoviral vectored vaccine.^5^ However, neutralising antibody response was not improved, with just over half the participants producing a response and at similar magnitudes to the dose-ranging participants who received two doses of 10 μg. In contrast to the age-dependent reactogenicity, age was not predictive of seroconversion or induction of neutralising antibodies.

In the 31 participants with prior COVID-19, the baseline binding antibody response was boosted in the majority of these participants after receiving only 1 μg. This was by a similar amount (≥0.5 log 10) to that observed following a third booster in the COV-Boost trial which evaluated seven different COVID-19 vaccines given as a third dose in those who had received two doses of ChAdOx1 or BNT162b2 as their primary schedule.^6^ Although there were some differences in reactogenicity reporting, there was no unexpected reactogenicity in the SARS-CoV2 seropositive group. Given that a long-term cost-effective vaccination strategy is needed to secure public health against the evolving COVID-19 pandemic in coming years, a low dose booster vaccine is of strategic interest.^1^ The potential to deliver an effective and well-tolerated boost with a single 1 μg dose of a next generation saRNA vaccine could provide advantages in relation to acceptability and cost effectiveness.

The local, systemic and laboratory reactions observed were similar in nature to the dose-ranging cohort and authorised vaccines. Of note, the proportion with neutropaenia was higher than expected due to natural variation, and this phenomenon has been observed with authorised COVID-19 vaccines^7^ and widely described in the typical response to vaccines against other unrelated infections.^8^ Following reports of a possible association between mRNA vaccines with myocarditis and/or pericarditis, particular attention was paid to cardiac events, but the only significant adverse event (exacerbation of myocardial ischaemia 30 weeks after the second vaccine) was in line with the participant's age and past medical history so not considered related to vaccine. More than 1 in 10 participants in the expanded safety cohort experienced a severe reaction following 10 μg but reactions to the 1.0 μg dose were largely mild, indicating a dose-related relationship with tolerability. The effect of age on tolerability, has also been reported following both authorised mRNA vaccines,^9^^,^^10^ and the Oxford-Astra Zeneca COVID-19 vaccine.^11^ Those at lowest risk of a life-threatening illness were most likely to experience a severe reaction which may deter individuals from completing the regimen at a population level. There was no association between age and immune responses which were similar in all age groups. For this reason, evaluation of a second-generation vaccine (LNP-nCoV saRNA-02) is proceeding with a maximum dose level of 5 μg (COVAC1 second-generation and COVAC Uganda ClinicalTrials.gov Identifier: NCT04934111).

The delay between the first and second vaccine may be the explanation for the higher rate of seroconversion in the expanded safety cohort as a longer gap facilitates the evolution of an anamnestic response and could also affect the adjuvant properties of the vaccine, but the lower first dose may also play an important role. It should also be noted that the dose level groups in dose-ranging cohort were small with a wide range of responses. Greater clinical protection against disease was observed in participants in the ChAdOx trial who received a low dose followed by the standard dose 8–12 weeks apart.^5^ However, this was not part of the randomised evaluation and the subset that received the low/standard dose regimen was younger compared to the overall trial population. Analyses of national vaccine programme data confirm the immune benefit and clinical protection of the longer gap between the Pfizer-BioNtech mRNA vaccines,^12^^,^^13^ supporting the decision to delay the second dose so that a first dose could be administered to a larger proportion of the population. This decision was taken in the context of authorised vaccines that provided substantial clinical benefit following a first dose^12^^,^^13^ and may not apply to future pandemic vaccines. Pharmaceutical companies are rightly cautious about mixed dose regimens due to the operational challenges, likely increased costs, and room for error in implementation, but a randomised evaluation of a low dose followed by a high dose and a variety of schedules would be of scientific value in future efficacy trials to determine the minimum dose and optimal schedule required. We observed a predominant response to the boosting dose, this contrasts to a recent pre-print of the Arcturus saRNA COVID-19 Phase 1/2 vaccine trial where the predominant response was elicited by the priming dose with limited benefit of a boosting dose reaching binding antibody levels overlapping but not above those of convalescent subjects.^14^

Binding IgG antibody responses against S were more frequent in the expanded safety cohort, although seroconversion rates were still not 100%. Binding antibody responses against S correlate with vaccine efficacy against symptomatic COVID-19.^15^ The magnitude of the responses observed with this first-generation formulation using a fractional priming dose and prolonged prime-boost schedule independent of age suggest that this technology could be further developed to produce an effective vaccine against COVID-19. Given that individuals with pre-existing baseline responses received a boost equivalent to authorised COVID-19 vaccines after only one dose of 1 μg, a next-generation saRNA vaccine, with competitive immunogenicity and updated to account for newer variants, could be placed to deliver boosting for an antigen-experienced global population in the future.

## Contributors

COVAC1 was designed by DTD, SMc, KMP and RS. KMP was the Chief Investigator and SMc provided clinical oversight. HMC coordinated the laboratory data collection and oversight. TC oversaw the pharmacy procedures. AS and HB conducted the analyses with advice from HMC, DTD, SMc, KMP and RS. MB, CC, SF, HH, VL, KMP and AW were site Principal Investigators. SMc, KMP and RS were study members of the Trial Steering Committee; HB, AS, DTD attended as observers. AS, DTD and SMc wrote the first draft of the paper and HB, KMP, HMC and RS contributed. All other authors contributed to the implementation of the study, analysis, and data collection. All authors critically reviewed and approved the final version.

## Data sharing statement

Data will be made available when the trial is complete, upon requests directed to the corresponding author and after approval of a proposal.

## Declaration of interests

R.J.S. is a co-inventor on a patent application covering this SARS-CoV-2 saRNA vaccine. All the other authors have nothing to report.

## Appendix A Supplementary data

Caption for supplementary material

COVAC1_supplementary_appendix 2v2

Appendix 1 Protocol v8
