# Supplementary material for: COVAC1 phase 2a expanded safety and immunogenicity study of a self-amplifying RNA vaccine against SARS-CoV-2
Source: eClinicalMedicine. 2023 Jan 13;56:101823. doi: 10.1016/j.eclinm.2022.101823 (PMC9837478; doi:10.1016/j.eclinm.2022.101823)
Supplement: Caption for supplementary material [file mmc1.docx]

**Caption for supplementary material**

Appendix 1: Study protocol

Appendix 2: Supplementary data
