# Supplementary material for: COVAC1 phase 2a expanded safety and immunogenicity study of a self-amplifying RNA vaccine against SARS-CoV-2
Source: eClinicalMedicine. 2023 Jan 13;56:101823. doi: 10.1016/j.eclinm.2022.101823 (PMC9837478; doi:10.1016/j.eclinm.2022.101823)
Supplement: COVAC1_supplementary_appendix 2v2 [file mmc2.docx]

**Supplementary information:**

**Safety and immunogenicity of a self-amplifying RNA vaccine against COVID-19: the expanded safety cohort of the COVAC1 trial**

Alex J. Szubert^a*^, Katrina M. Pollock^b, h*^, Hannah M. Cheeseman^b^, Jasmini Alagaratnam^b^, Henry Bern^a^, Olivia Bird^c^, Marta Boffito^d^, Ruth Byrne^d^, Tom Cole^h^, Catherine A. Cosgrove^c^, Saul N. Faust^e^, Sarah Fidler^b^, Eva Galiza^c^, Hana Hassanin^f^, Mohini Kalyan^b^, Vincenzo Libri^g^, Leon R. McFarlane^b^, Ana Milinkovic^d^, Jessica O’Hara^b^, David R. Owen^h, 1^, Daniel Owens^e^, Mihaela Pacurar^e^, Tommy Rampling^g^, Simon Skene^f^, Alan Winston^b^, James Woolley^f^, Yee Ting N. Yim^g^, David T. Dunn^a^, Sheena McCormack^a^, Robin J Shattock^b^ on behalf of the COVAC 1 Study Team

**The COVAC1 study Group:**

Kirsty Adams^g^, Fahimah Amini^b^, Nafisah B Atako^a^, Wendy Barclay^b^, Lisa Berry^e^, Caroline Bodinham^f^, Thomas Bower^e^, Elizabeth Brodnicki^a^, Alvin Daramola-Rose^a^, Suzanne Day^b^, Monica Desai^a^, Max Dinc^g^, Tamara Elliott^b^, Katie Flight^b^, Paul Heath^c^, Cecilia Hultin^c^, Sabina Ikram^c^, Lester G Macabodbod^d^, Lindsey Masters^a^, Ian McGuiness^b^, Maya Moshe^b^, Alasdair Munro^e^, Vincent Osabutey-Anikon^b^, Jennifer Pearce^c^, Claire Petersen^h^, Mary Rauchenberger^a^, Marivic Ricamara^g^, Susie Slater^a^, Ben Spittle^a^, Aminata Sy^a^, Nicola Turner^g^, Denise Ward^a^, Martin Wilkins^h^

^a^ cMRC Clinical Trials Unit at UCL, London, UK

^b^Department of Infectious Disease, Imperial College London

^c^St George’s Vaccine Institute, Institute for Infection and Immunity, St George’s University of London

^d^Chelsea & Westminster Hospital, London

^e^NIHR Southampton Clinical Research Facility and Biomedical Research Centre, University Hospital Southampton NHS Foundation Trust, Southampton, UK; Faculty of Medicine and Institute for Life Sciences, University of Southampton, Southampton, UK

^f^Surrey Clinical Research Facility, Faculty of Health and Medical Sciences, University of Surrey, Guildford, UK

^g^dNIHR UCLH Clinical Research Facility and NIHR UCLH Biomedical Research Centre, London, UK

^h^NIHR Imperial Clinical Research Facility and NIHR Imperial Biomedical Research Centre, London, UK

^i^Department of Brain Sciences, Imperial College London, London, UK

*Contributed equally, †Listed in the Supplementary, ^ø^corresponding authors: [r.shattock@ic.ac.uk](mailto:r.shattock@ic.ac.uk), s.mccormack@ucl.ac.uk

**Table of Contents**

[Description of trial, inclusion and exclusion criteria 4](#_Toc122443109)

[Table 1 Trial Schema. 4](#_Toc122443110)

[Table 2 Screening and enrolment by centre 4](#_Toc122443111)

[Table 3 Grade criteria for adverse events (AE), adapted from the FDA toxicity table for healthy volunteers 6](#_Toc122443112)

[Table 4 Reasons for ineligibility 7](#_Toc122443113)

[Table 5 Authorised/licensed COVID vaccines by the end of follow-up (52 weeks per participant) 7](#_Toc122443114)

[Supplementary Safety Tables and Figures 8](#_Toc122443115)

[Table 6.1 Solicited local injection site reactions starting within 7 days of administration of the vaccine, regardless of relationship to IMP for first and second vaccine 9](#_Toc122443116)

[Table 6.2 Local injection site reactions starting within 7 days of the first vaccine 10](#_Toc122443117)

[Table 6.3 Local injection site reactions starting within 7 days of the second vaccine 11](#_Toc122443118)

[Table 6.4 Solicited systemic reactions starting within 7 days of administration of the vaccine and including routine laboratory parameters, regardless of relationship to IMP, for first and second vaccine 12](#_Toc122443119)

[Table 6.5 Systemic reactions starting within 7 days of the first vaccine 15](#_Toc122443120)

[Table 6.6 Systemic reactions starting within 7 days of the second vaccine 18](#_Toc122443121)

[Figure 1 Solicited local injection site reactions starting within 7 days of the second vaccine for participants who received 1.0 µg followed by 10.0 µg 21](#_Toc122443122)

[Figure 2 Solicited systemic reactions that started within 7 days of second vaccine for participants who received 1.0 µg followed by 10.0 µg 22](#_Toc122443123)

[Table 6.7 Local injection site reactions starting within 7 days of the first vaccine by age at enrolment for participants who received 1.0 µg (dose-ranging cohort and expanded safety cohort) 23](#_Toc122443124)

[Table 6.8 Systemic reactions starting within 7 days of the first vaccine by age at enrolment for participants who received 1.0 µg (dose-ranging cohort and expanded safety cohort) 24](#_Toc122443125)

[Table 6.9 Local injection site reactions starting within 7 days of the second vaccine by age at enrolment for participants who received 1.0 µg followed by 10.0 µg (expanded safety cohort only) 27](#_Toc122443126)

[Table 6.10 Systemic reactions starting within 7 days of the second vaccine by age at enrolment for participants who received 1.0 µg followed by 10.0 µg (expanded safety cohort only) 28](#_Toc122443127)

[Table 6.11 Local injection site reactions starting within 7 days of the first vaccine by history of COVID-19 infection for participants who received 1.0 µg (dose-ranging cohort/expanded safety cohort) 31](#_Toc122443128)

[Table 6.12 Systemic reactions starting within 7 days of the first vaccine by history of COVID-19 infection for participants who received 1.0 µg (dose-ranging cohort/expanded safety cohort) 32](#_Toc122443129)

[Table 6.13 Local injection site reactions starting within 7 days of the second vaccine by history of COVID-19 infection for participants who received 1.0 µg followed by 10.0 µg (expanded safety cohort) 34](#_Toc122443130)

[Table 6.14 Systemic reactions starting within 7 days of the second vaccine by history of COVID-19 infection for participants who received 1.0 µg followed by 10.0 µg (expanded safety cohort) 35](#_Toc122443131)

[Table 6.15 Other adverse events (AEs), regardless of relationship to IMP 37](#_Toc122443132)

[Table 6.16 Other adverse reactions (ARs) 55](#_Toc122443133)

[Figures 3-13 Change in laboratory safety parameters 61](#_Toc122443134)

[Figure 14. Pseudoneutralising antibodies (IC50) against and Omicron BA.5 67](#_Toc122443135)

[Table 7. Adjusted analysis for comparison for 1/10 ug versus 10/19 ug cohorts 68](#_Toc122443136)

[Figure 15 Individual immune responses in baseline seropositive participants in the expanded safety cohort 69](#_Toc122443137)

# Description of trial, inclusion and exclusion criteria

## Table 1 Trial Schema.

| **Group** | **Description** | **Allocation** | **Dose prime (μg)** | **Dose boost (μg)** | **N** |
| --- | --- | --- | --- | --- | --- |
| C | Expanded safety | Open-label | 1 | - | 5 |
|  |  |  | 1 | 1 | 1 |
|  |  |  | 1 | 10 | 216 |

Inclusion and exclusion criteria are listed on p4-5 and reasons for ineligibility in Table 4, p7.

## Table 2 Screening and enrolment by centre

| **Centre** | **Screened** | **Enrolled** | **Date of first enrolment** | **Date of latest enrolment** |
| --- | --- | --- | --- | --- |
| Chelsea and Westminster | 37 | 29 | 10Aug2020 | 20Aug2020 |
| St Georges | 83 | 37 | 11Aug2020 | 13Aug2020 |
| St Marys | 57 | 37 | 10Aug2020 | 17Aug2020 |
| University Hospital Southampton | 56 | 40 | 11Aug2020 | 17Aug2020 |
| Surrey CRF | 55 | 38 | 11Aug2020 | 14Aug2020 |
| UCLH | 99 | 41 | 10Aug2020 | 14Aug2020 |
| Total | 387 | 222 | 10Aug2020 | 20Aug2020 |

**Inclusion criteria**

1. Healthy adults from the following age ranges:
   1. For the dose escalation and evaluation, aged 18-45 years on the day of screening
   2. For the expanded safety evaluation, aged 18-75 years on the day of screening
2. At similar risk of acquiring SARS-CoV-2 infection to the general population
3. Willing and able to provide written informed consent
4. If female and of childbearing^i^ potential, willing to use a highly effective method^ii^ of contraception from screening until 18 weeks^iv^ after last injection
5. If male and not sterilised, willing to avoid impregnating female partners^iii^ from screening until 18 weeks^iv^ after last injection
6. Willing to avoid all other vaccines from within 4 weeks before the first injection through to 4 weeks after the second injection^v^
7. Willing and able to comply with visit schedule, complete online diaries and provide samples
8. Willing to grant authorised persons access to his/her trial-related medical record and GP records either directly or indirectly

i A woman will be considered of childbearing potential following menarche and until becoming post-menopausal unless permanently sterile. Permanent sterilisation methods include hysterectomy, bilateral salpingectomy and bilateral oophorectomy. A post-menopausal state is defined as no menses for 12 months without an alternative medical cause.

ii The following methods are considered highly effective:

- combined (estrogen and progestogen containing) hormonal contraception associated with inhibition of ovulation – oral, intravaginal or transdermal;
- progestogen-only hormonal contraception associated with inhibition of ovulation – oral, injectable or implantable
- intrauterine device (IUD);
- intrauterine hormone-releasing system (IUS);
- bilateral tubal occlusion;
- vasectomised partner, where the vasectomised partner has received medical assessment of the surgical success; and
- sexual abstinence, defined as refraining from heterosexual intercourse – must be the preferred and usual lifestyle of the participant.

iii Through the use of condoms or sexual abstinence (see definition in footnote ii above)

iv Nonclinical studies of saRNAs [48] showed maximal expression of the vaccine immunogen at 7 days post-immunisation, approaching baseline by 3 weeks post-immunisation, with some residual very low expression seen out to 9 weeks. Biodistribution studies with LNP-nCoVsaRNA are planned, but in the absence of data we wish to take a conservative approach to the contraception period, and require an 18-week washout period.

v The exception is the flu vaccine, which participants may have – provided they have it at least 7 days before or after a study vaccination. It is recommended that participants have an up to date vaccination status for any required immunisations.

**Exclusion criteria**

1. Pregnant or lactating
2. Has a significant clinical history, physical finding on clinical examination during screening, or presence of a disease that is active or requires treatment to control it, including cardiac, respiratory, endocrine, metabolic, autoimmune, liver, neurological, oncological, psychiatric, immunosuppresive/immunodeficient or other disorders which in the opinion of the investigator is not compatible with healthy status, increases the risk of severe COVID-19, may compromise the volunteer’s safety, preclude vaccination or compromise interpretation of the immune response to vaccine. Individuals with mild/moderate, well-controlled comorbidities are allowed.
3. History of COVID-19 infection^i^
4. History of anaphylaxis or angioedema
5. History of severe or multiple allergies to drugs or pharmaceutical agents
6. History of severe local or general reaction to vaccination defined as:
   1. local: extensive, indurated redness and swelling involving most of the arm, not resolving within 72 hours
   2. general: fever ≥39.5 °C within 48 hours; bronchospasm; laryngeal oedema; collapse; convulsions or encephalopathy within 72 hours
7. Ever received an experimental or authorised vaccine against COVID-19
8. Receipt of any immunosuppressive agents within 18 weeks of screening by any route other than topical
9. Detection of antibodies to hepatitis C
10. Detection of antibodies to HIV
11. Grade 1^ii^ and above abnormalities in routine laboratory parameters (see Table 3) using the FDA toxicity table Toxicity Grading Scale for Healthy Adult and Adolescent Volunteers Enrolled in Preventive Vaccine Clinical Trials, taking account of local laboratory reference ranges. <https://www.fda.gov/media/73679/download>
12. Participating in another clinical trial with an investigational drug or device, or treated with an investigational drug within 28 days of screening.
13. Has received an immunisation within 28 days of screening

i This exclusion criterion applies at all sites with the exception of the St Mary’s Hospital Site, which will preferentially enrol participants known to have infection-induced SARS-CoV-2 antibodies prior to enrolment
ii Trace of protein and/or blood on dipstick urinalysis and ALT/AST ≤1.2 x ULN are not exclusion

## Table 3 Grade criteria for adverse events (AE), adapted from the FDA toxicity table for healthy volunteers

| **Solicited AE** | **Grade 1** | **Grade 2** | **Grade 3** | **Grade 4** |
| --- | --- | --- | --- | --- |
| **Local to injection site** | | | | |
| Pain | Does not interfere with activity | Repeated use of non-narcotic pain reliever > 24 hours/interferes with activity | Any use of narcotic pain reliever or prevents daily activity | Emergency room  (ER) visit or  hospitalization |
| Tenderness/ discomfort | Mild discomfort to touch | Discomfort with movement | Significant discomfort at rest | ER visit or  hospitalization |
| Redness | 2.5 – 5 cm | 5.1 – 10 cm | > 10 cm | Necrosis or  exfoliative dermatitis |
| Swelling | 2.5 – 5 cm and does not interfere with activity | 5.1 – 10 cm or  interferes with activity | > 10 cm or  prevents daily activity | Necrosis |
| **Systemic** | | | | |
| Temperature | 38.0 – 38.4 | 38.5 – 38.9 | 39.0 – 40 | > 40 |
| Chills/ Shivering | No interference with activity | Some interference  with activity | Significant;  prevents daily  activity | ER visit or  hospitalization |
| Myalgia |  |  |  |  |
| Arthralgia |  |  |  |  |
| Fatigue |  |  |  |  |
| Headache | No interference with activity | Repeated use of non-narcotic pain reliever > 24 hours/some interference with activity | Significant; any use of narcotic pain reliever/prevents daily activity | ER visit or  hospitalization |
| Nausea | No interference with activity or 1 – 2 episodes/24 hours | Some interference with activity or > 2 episodes/24 hours | Prevents daily activity, requires outpatient IV hydration | ER visit or  hospitalization for hypotensive shock |
| Vomiting | No interference  with activity or 1 – 2 episodes/24 hours | Some interference with activity or > 2 episodes/24 hours | Prevents daily activity, requires outpatient IV hydration | ER visit or  hospitalization for hypotensive shock |
| **Laboratory values** | | | | |
| Creatinine | 133 – 150 (M)  116 – 150 (F) | 151 – 177 | 178 – 221 | > 221 or requires dialysis |
| Aspartate transaminase | 1.2 - 2.5 x ULN | > 2.5 - 5.0 x ULN | > 5 - 10 x ULN | > 10 x ULN |
| Alanine transaminase | 1.2 - 2.5 x ULN | > 2.5 - 5.0 x ULN | > 5 - 10 x ULN | > 10 x ULN |
| Alkaline phosphatase | 1.1 - 2.0 x ULN | > 2.0 - 3.0 x ULN | > 3.0 - 10 x ULN | > 10 x ULN |
| Total bilirubin | 1.1 - 1.5 x ULN | >1.5 - 2.0 x ULN | >2.0 - 3.0 x ULN | > 3.0 x ULN |
| Non-fasting glucose | (high) 7.9 - 9.0;  (low) 2.7 - 2.9 | (high) > 9.0 - 14.4;  (low) 2.4 - < 2.7 | (high) > 14.4;  (low) 1.9 - < 2.4 | (high) insulin/coma;  (low) < 1.9 |
| Haemoglobin | 125 – 129 (M)  110 – 113 (F) | 105 - < 125 (M)  95 - < 110 (F) | 85 - < 105 (M)  80 - < 95 (F) | < 85 (M)  < 80 (F) |
| Total white cell count | (M high) 10.8 - 15;  (F high) 11.3 - 15;  (low) 2.5 - 3.5 | (high) > 15 - 20;  (low) 1.5 - < 2.5 | (high) > 20 - 25;  (low) 1.0 - < 1.5 | (high) > 25;  (low) < 1.0 |
| Lymphocytes | .75 - 1.0 | .5 - <.75 | .25 - < .5 | < .25 |
| Neutrophils | 1.5 - 1.9 | 1.0 - < 1.5 | .5 - < 1.0 | < .5 |
| Platelets | 125 – 129 (M)  125 – 134 (F) | 100 - < 125 | 25 - < 100 | < 25 |

Table 4 Reasons for ineligibility*****

| **Reason** | **Number of subjects** |
| --- | --- |
| Not willing to use a highly effective method of contraception | 5 |
| Not willing to comply with visit schedule | 16 |
| Not willing to grant access to medical record | 1 |
| Comorbidities | 42 |
| History of COVID | 6 |
| History of anaphylaxis or angioedema | 5 |
| History of allergies | 2 |
| History of severe reaction to vaccination | 2 |
| Immunosuppressive agents | 8 |
| COVID antibodies | 2 |
| HIV | 1 |
| Grade 1 and above abnormalities in routine laboratory parameters | 59 |
| Over-screened | 23 |
| **Total** | 165 |
| *Reasons are not mutually exclusive therefore total to more than the total number of non-enrolments | |

## Table 5 Authorised/licensed COVID vaccines by the end of follow-up (52 weeks per participant)

|  | **Expanded safety cohort N=222** |
| --- | --- |
| **Known to have received an authorised/licensed COVID-19 vaccine injection?** | |
| Yes | 219 (98.6%) |
| No | 3 (1.4%) |
|  |  |
| **Vaccine** | |
| ChAdOx Injection 1 and Injection 2 | 94 (42.3%) |
| ChAdOx Injection 1 | 5 (2.3%) |
| BioNTech/Pfizer BNT162b2 LNP-RNA Injection 1 and Injection 2 | 86 (38.7%) |
| BioNTech/Pfizer BNT162b2 LNP-RNA Injection 1 | 25 (11.3%) |
| Moderna mRNA-1273 SARS-CoV-2 Injection 1 and Injection 2 | 4 (1.8%) |
| Moderna mRNA-1273 SARS-CoV-2 Injection 1 | 5 (2.3%) |
| Not known to have received an authorised/licensed COVID-19 vaccine injection | 3 (1.4%) |
|  |  |
| **Day post-enrolment** | |
| Mean (SD) | 208.8 (53.72) |
| Median (IQR) | 196.0 (171.0, 256.0) |
| Range | 126.0-318.0 |
| Not known to have received an authorised/licensed COVID-19 vaccine injection | 3 |
|  |  |
| **Day post-vaccine 2** | |
| Mean (SD) | 108.5 (53.03) |
| Median (IQR) | 100.0 (68.0, 153.0) |
| Range | 21.0-214.0 |
| Received vaccine 2 and not known to have received an authorised/licensed COVID-19 vaccine injection | 2 |
|  |  |
| **Day post-vaccine 2** | |
| 14-27 | 2 (0.9%) |
| 28-55 | 36 (16.6%) |
| >= 56 | 177 (81.6%) |
| Received vaccine 2 and not known to have received an authorised/licensed COVID-19 vaccine injection | 2 (0.9%) |

# Supplementary Safety Tables and Figures

Participants were sent a reminder to complete an electronic diary from the evening following vaccination and for six further evenings, recording the presence and severity of the local and systemic solicited adverse events listed in [**Table 3**](#_Table_3_Grade). Including first and second vaccines, 84.1% of expected diary cards were completed in full. Each card includes 12 questions. Including all expected cards (including any not completed, or completed but not in full), 92.8% of questions were answered.

The study team checked the electronic diaries and recorded solicited adverse events in the electronic data capture system within an hour after vaccination, and at the visit scheduled 7 days following each vaccine when laboratory adverse events were also checked. Note that 99.7% of expected solicited events CRFs were completed for first and second vaccines. Each CRF includes 12, 'key,' questions (corresponding to 12 solicited events). Including all expected CRFs (including any not completed), 99.4% of, 'key,' questions were answered.

Solicited adverse events local to the site of injection following first and second vaccine are summarised by grade in [**Table 6.1**](#_Table_5.1_Solicited), following the first vaccine in [**Table 6.2**](#_Table_5.2_Local) and following the second vaccine in [**Table 6.3**](#_Table_5.3_Local). Solicited systemic events including laboratory events are similarly summarised in [**Tables 6.4**](#_Table_5.4_Solicited)**,** [**6.5**](#_Table_5.5_Systemic) **and** [**6.6**](#_Table_5.6_Systemic).

[**Figure 1**](#_Figure_1_Solicited) shows the pattern of solicited adverse events local to the site of injection following the second vaccine for participants who received 1.0 µg followed by 10.0 µg, and [**Figure 2**](#_Figure_2_Solicited) shows the pattern of solicited systemic events other than laboratory events after the second vaccine for participants who received 1.0 µg followed by 10.0 µg.

Solicited adverse events local to the site of injection following first vaccine for participants who received 1.0 µg (dose-ranging cohort/expanded safety cohort) are summarised by grade and by age at enrolment in [**Table 6.**](#_Table_5.1_Solicited)**7** and solicited systemic events including laboratory events in [**Table 6.**](#_Table_5.3_Local)**8**. Events following second vaccine for participants who received 1.0 µg followed by 10.0 µg (expanded safety cohort) are similarly summarised in [**Tables 6.**](#_Table_5.4_Solicited)**9 and** [**6.**](#_Table_5.6_Systemic)**10**.

Solicited adverse events local to the site of injection following first vaccine for participants who received 1.0 µg (dose-ranging cohort/expanded safety cohort) are summarised by grade and by history of COVID-19 infection in [**Table 6.**](#_Table_5.1_Solicited)**11** and solicited systemic events including laboratory events in [**Table 6.**](#_Table_5.3_Local)**12**. Events following second vaccine for participants who received 1.0 µg followed by 10.0 µg (expanded safety cohort) are similarly summarised in [**Tables 6.**](#_Table_5.4_Solicited)**13 and** [**6.**](#_Table_5.6_Systemic)**14**.

Participants were asked about adverse events at every visit and study staff recorded these in the eDC Adverse Event Log noting severity and relationship to vaccine. Laboratory parameters were scheduled for 7, 14 and 28 days after the first vaccine; on the day of the second vaccine (pre-vaccination); 7, 14, 28 and 56 days after the second vaccine; and at day 364. Severity was judged according to the FDA Toxicity Grading Scale for Healthy Adult and Adolescent Volunteers Enrolled in Preventive Vaccine Clinical Trials, taking account of local laboratory reference ranges.

All adverse events captured on the Adverse Event log and laboratory events (excluding solicited adverse reactions starting within 7 days of administration of the vaccine), regardless of relationship to vaccine are presented in [**Table 6.**](#_Table_5.8_Headache)**15.** [**Table 6.**](#_Table_5.9_Any)**16** is limited to adverse events and laboratory abnormalities where a relationship to vaccine was recorded as possible, probable or definite on the log. The events are coded using the MedDRA coding system preferred term and presented by system organ classification and grade.

The actual change in the 11 laboratory parameters from baseline values 7, 14 and 28 days after the first vaccine; on the day of second vaccine; 7, 14, 28 and 56 days after the second vaccine; and at day 364 are shown in [**Figures 3-13**](#_Figures_3-13_Change).

Differences in pseudo-neutralization titres against Wuhan and Omicron BA.5 are shown in Figure 14

## Table 6.1 Solicited local injection site reactions starting within 7 days of administration of the vaccine, regardless of relationship to IMP for first and second vaccine

|  | **Expanded safety cohort N=222** |
| --- | --- |
| **Any** | |
| Normal | 13 (5.9%) |
| Grade 1 | 134 (60.4%) |
| Grade 2 | 73 (32.9%) |
| Grade 3 | 2 (0.9%) |
|  |  |
| **Pain** | |
| Normal | 67 (30.2%) |
| Grade 1 | 111 (50.0%) |
| Grade 2 | 43 (19.4%) |
| Grade 3 | 1 (0.5%) |
|  |  |
| **Tenderness/discomfort** | |
| Normal | 19 (8.6%) |
| Grade 1 | 135 (60.8%) |
| Grade 2 | 67 (30.2%) |
| Grade 3 | 1 (0.5%) |
|  |  |
| **Erythema/redness** | |
| Normal | 213 (95.9%) |
| Grade 1 | 8 (3.6%) |
| Grade 2 | 1 (0.5%) |
|  |  |
| **Induration/swelling** | |
| Normal | 216 (97.3%) |
| Grade 1 | 6 (2.7%) |

## Table 6.2 Local injection site reactions starting within 7 days of the first vaccine

|  | **Dose-ranging cohort: 1.0 µg N=42*** | **Expanded safety cohort: 1.0 µg N=222** |
| --- | --- | --- |
| **Any** | | |
| Normal | 9 (21.4%) | 105 (47.3%) |
| Grade 1 | 32 (76.2%) | 111 (50.0%) |
| Grade 2 | 1 (2.4%) | 6 (2.7%) |
|  |  |  |
| **Pain** | | |
| Normal | 29 (69.0%) | 177 (79.7%) |
| Grade 1 | 12 (28.6%) | 41 (18.5%) |
| Grade 2 | 1 (2.4%) | 4 (1.8%) |
|  |  |  |
| **Tenderness/discomfort** | | |
| Normal | 11 (26.2%) | 113 (50.9%) |
| Grade 1 | 30 (71.4%) | 104 (46.8%) |
| Grade 2 | 1 (2.4%) | 5 (2.3%) |
|  |  |  |
| **Erythema/redness** | | |
| Normal | 42 (100.0%) | 216 (97.3%) |
| Grade 1 | 0 (0.0%) | 6 (2.7%) |
|  |  |  |
| **Induration/swelling** | | |
| Normal | 42 (100.0%) | 221 (99.5%) |
| Grade 1 | 0 (0.0%) | 1 (0.5%) |

*Pollock et al, eClinicalMedicine, 2022. N.B. Dose-ranging cohort aged 18-45 years; expanded safety cohort 18-75 years

## Table 6.3 Local injection site reactions starting within 7 days of the second vaccine

|  | **Dose-ranging cohort: 10.0 µg followed by 10.0 µg N=24*** | **Expanded safety cohort: 1.0 µg followed by 10.0 µg N=216**** |
| --- | --- | --- |
| **Any** | | |
| Normal | 1 (4.2%) | 14 (6.5%) |
| Grade 1 | 19 (79.2%) | 130 (60.2%) |
| Grade 2 | 4 (16.7%) | 70 (32.4%) |
| Grade 3 | 0 (0.0%) | 2 (0.9%) |
|  |  |  |
| **Pain** | | |
| Normal | 9 (37.5%) | 68 (31.5%) |
| Grade 1 | 13 (54.2%) | 107 (49.5%) |
| Grade 2 | 2 (8.3%) | 40 (18.5%) |
| Grade 3 | 0 (0.0%) | 1 (0.5%) |
|  |  |  |
| **Tenderness/discomfort** | | |
| Normal | 1 (4.2%) | 22 (10.2%) |
| Grade 1 | 20 (83.3%) | 129 (59.7%) |
| Grade 2 | 3 (12.5%) | 64 (29.6%) |
| Grade 3 | 0 (0.0%) | 1 (0.5%) |
|  |  |  |
| **Erythema/redness** | | |
| Normal | 24 (100.0%) | 212 (98.1%) |
| Grade 1 | 0 (0.0%) | 3 (1.4%) |
| Grade 2 | 0 (0.0%) | 1 (0.5%) |
|  |  |  |
| **Induration/swelling** | | |
| Normal | 24 (100.0%) | 211 (97.7%) |
| Grade 1 | 0 (0.0%) | 5 (2.3%) |

*Pollock et al, eClinicalMedicine, 2022. N.B. Dose-ranging cohort aged 18-45 years; expanded safety cohort 18-75 years

**One further participant received 1.0 µg followed by 1.0 µg, and did not experience any local injection site reactions starting within 7 days of the second vaccine

## Table 6.4 Solicited systemic reactions starting within 7 days of administration of the vaccine and including routine laboratory parameters, regardless of relationship to IMP, for first and second vaccine

|  | **Expanded safety cohort N=222** |
| --- | --- |
| **Any** | |
| Normal | 20 (9.0%) |
| Grade 1 | 69 (31.1%) |
| Grade 2 | 109 (49.1%) |
| Grade 3 | 24 (10.8%) |
|  |  |
| **Temperature** | |
| Normal | 193 (86.9%) |
| Grade 1 | 19 (8.6%) |
| Grade 2 | 4 (1.8%) |
| Grade 3 | 6 (2.7%) |
|  |  |
| **Chills/shivering** | |
| Normal | 92 (41.4%) |
| Grade 1 | 50 (22.5%) |
| Grade 2 | 74 (33.3%) |
| Grade 3 | 6 (2.7%) |
|  |  |
| **Myalgia (flu-like general muscle aches)** | |
| Normal | 84 (37.8%) |
| Grade 1 | 64 (28.8%) |
| Grade 2 | 68 (30.6%) |
| Grade 3 | 6 (2.7%) |
|  |  |
| **Arthralgia** | |
| Normal | 129 (58.1%) |
| Grade 1 | 57 (25.7%) |
| Grade 2 | 34 (15.3%) |
| Grade 3 | 2 (0.9%) |
|  |  |
| **Fatigue** | |
| Normal | 54 (24.3%) |
| Grade 1 | 80 (36.0%) |
| Grade 2 | 75 (33.8%) |
| Grade 3 | 13 (5.9%) |
|  |  |
| **Headache** | |
| Normal | 61 (27.5%) |
| Grade 1 | 79 (35.6%) |
| Grade 2 | 74 (33.3%) |
| Grade 3 | 8 (3.6%) |
|  |  |
| **Nausea** | |
| Normal | 154 (69.4%) |
| Grade 1 | 48 (21.6%) |
| Grade 2 | 18 (8.1%) |
| Grade 3 | 2 (0.9%) |
|  |  |
| **Vomiting** | |
| Normal | 211 (95.0%) |
| Grade 1 | 7 (3.2%) |
| Grade 2 | 4 (1.8%) |
|  |  |
| **Creatinine** | |
| Normal | 220 (99.1%) |
| Grade 1 | 2 (0.9%) |
|  |  |
| **ALT** | |
| Normal | 209 (94.1%) |
| Grade 1 | 13 (5.9%) |
|  |  |
| **AST** | |
| Normal | 180 (81.1%) |
| Grade 1 | 5 (2.3%) |
| Not known | 37 (16.7%) |
|  |  |
| **Alkaline phosphate** | |
| Normal | 222 (100.0%) |
|  |  |
| **Bilirubin** | |
| Normal | 217 (97.7%) |
| Grade 1 | 5 (2.3%) |
|  |  |
| **Glucose (non-fasting) (high or low)** | |
| Normal | 220 (99.1%) |
| Grade 2 | 2 (0.9%) |
|  |  |
| **Haemoglobin** | |
| Normal | 217 (97.7%) |
| Grade 1 | 4 (1.8%) |
| Grade 2 | 1 (0.5%) |
|  |  |
| **White blood cell count (high or low)** | |
| Normal | 204 (91.9%) |
| Grade 1 | 18 (8.1%) |
|  |  |
| **Neutrophils** | |
| Normal | 189 (85.1%) |
| Grade 1 | 27 (12.2%) |
| Grade 2 | 5 (2.3%) |
| Grade 3 | 1 (0.5%) |
|  |  |
| **Lymphoctyes** | |
| Normal | 211 (95.0%) |
| Grade 1 | 10 (4.5%) |
| Grade 2 | 1 (0.5%) |
|  |  |
| **Platelets** | |
| Normal | 221 (99.5%) |
| Grade 2 | 1 (0.5%) |

## Table 6.5 Systemic reactions starting within 7 days of the first vaccine

|  | **Dose-ranging cohort: 1.0 µg N=42*** | **Expanded safety cohort: 1.0 µg N=222** |
| --- | --- | --- |
| **Any** | | |
| Normal | 18 (42.9%) | 94 (42.3%) |
| Grade 1 | 20 (47.6%) | 102 (45.9%) |
| Grade 2 | 4 (9.5%) | 25 (11.3%) |
| Grade 3 | 0 (0.0%) | 1 (0.5%) |
|  |  |  |
| **Temperature** | | |
| Normal | 42 (100.0%) | 221 (99.5%) |
| Grade 1 | 0 (0.0%) | 1 (0.5%) |
|  |  |  |
| **Chills/shivering** | | |
| Normal | 42 (100.0%) | 216 (97.3%) |
| Grade 1 | 0 (0.0%) | 4 (1.8%) |
| Grade 2 | 0 (0.0%) | 2 (0.9%) |
|  |  |  |
| **Myalgia (flu-like general muscle aches)** | | |
| Normal | 39 (92.9%) | 203 (91.4%) |
| Grade 1 | 2 (4.8%) | 15 (6.8%) |
| Grade 2 | 1 (2.4%) | 4 (1.8%) |
|  |  |  |
| **Arthralgia** | | |
| Normal | 40 (95.2%) | 205 (92.3%) |
| Grade 1 | 2 (4.8%) | 16 (7.2%) |
| Grade 2 | 0 (0.0%) | 1 (0.5%) |
|  |  |  |
| **Fatigue** | | |
| Normal | 32 (76.2%) | 157 (70.7%) |
| Grade 1 | 7 (16.7%) | 52 (23.4%) |
| Grade 2 | 3 (7.1%) | 13 (5.9%) |
|  |  |  |
| **Headache** | | |
| Normal | 28 (66.7%) | 157 (70.7%) |
| Grade 1 | 13 (31.0%) | 57 (25.7%) |
| Grade 2 | 1 (2.4%) | 8 (3.6%) |
|  |  |  |
| **Nausea** | | |
| Normal | 38 (90.5%) | 206 (92.8%) |
| Grade 1 | 4 (9.5%) | 13 (5.9%) |
| Grade 2 | 0 (0.0%) | 3 (1.4%) |
|  |  |  |
| **Vomiting** | | |
| Normal | 42 (100.0%) | 221 (99.5%) |
| Grade 1 | 0 (0.0%) | 1 (0.5%) |
|  |  |  |
| **Creatinine** | | |
| Normal | 42 (100.0%) | 215 (96.8%) |
| Grade 1 | 0 (0.0%) | 1 (0.5%) |
| Not known | 0 (0.0%) | 6 (2.7%) |
|  |  |  |
| **ALT** | | |
| Normal | 41 (97.6%) | 213 (95.9%) |
| Grade 1 | 1 (2.4%) | 2 (0.9%) |
| Not known | 0 (0.0%) | 7 (3.2%) |
|  |  |  |
| **AST** | | |
| Normal | 40 (95.2%) | 179 (80.6%) |
| Grade 1 | 2 (4.8%) | 2 (0.9%) |
| Not known | 0 (0.0%) | 41 (18.5%) |
|  |  |  |
| **Alkaline phosphate** | | |
| Normal | 42 (100.0%) | 215 (96.8%) |
| Not known | 0 (0.0%) | 7 (3.2%) |
|  |  |  |
| **Bilirubin** | | |
| Normal | 42 (100.0%) | 212 (95.5%) |
| Grade 1 | 0 (0.0%) | 3 (1.4%) |
| Not known | 0 (0.0%) | 7 (3.2%) |
|  |  |  |
| **Glucose (non-fasting) (high or low)** | | |
| Normal | 41 (97.6%) | 215 (96.8%) |
| Grade 1 | 1 (2.4%) | 0 (0.0%) |
| Grade 2 | 0 (0.0%) | 1 (0.5%) |
| Not known | 0 (0.0%) | 6 (2.7%) |
|  |  |  |
| **Haemoglobin** | | |
| Normal | 42 (100.0%) | 212 (95.5%) |
| Grade 1 | 0 (0.0%) | 4 (1.8%) |
| Not known | 0 (0.0%) | 6 (2.7%) |
|  |  |  |
| **White blood cell count (high or low)** | | |
| Normal | 41 (97.6%) | 207 (93.2%) |
| Grade 1 | 1 (2.4%) | 9 (4.1%) |
| Not known | 0 (0.0%) | 6 (2.7%) |
|  |  |  |
| **Neutrophils** | | |
| Normal | 39 (92.9%) | 202 (91.0%) |
| Grade 1 | 3 (7.1%) | 11 (5.0%) |
| Grade 2 | 0 (0.0%) | 2 (0.9%) |
| Grade 3 | 0 (0.0%) | 1 (0.5%) |
| Not known | 0 (0.0%) | 6 (2.7%) |
|  |  |  |
| **Lymphoctyes** | | |
| Normal | 41 (97.6%) | 209 (94.1%) |
| Grade 1 | 1 (2.4%) | 6 (2.7%) |
| Grade 2 | 0 (0.0%) | 1 (0.5%) |
| Not known | 0 (0.0%) | 6 (2.7%) |
|  |  |  |
| **Platelets** | | |
| Normal | 42 (100.0%) | 215 (96.8%) |
| Grade 2 | 0 (0.0%) | 1 (0.5%) |
| Not known | 0 (0.0%) | 6 (2.7%) |

*Pollock et al, eClinicalMedicine, 2022. N.B. Dose-ranging cohort aged 18-45 years; expanded safety cohort 18-75 years

## Table 6.6 Systemic reactions starting within 7 days of the second vaccine

|  | **Dose-ranging cohort: 10.0 µg followed by 10.0 µg N=24*** | **Expanded safety cohort: 1.0 µg followed by 10.0 µg N=216**** |
| --- | --- | --- |
| **Any** | | |
| Normal | 0 (0.0%) | 25 (11.6%) |
| Grade 1 | 8 (33.3%) | 61 (28.2%) |
| Grade 2 | 13 (54.2%) | 106 (49.1%) |
| Grade 3 | 3 (12.5%) | 24 (11.1%) |
|  |  |  |
| **Temperature** | | |
| Normal | 20 (83.3%) | 188 (87.0%) |
| Grade 1 | 2 (8.3%) | 18 (8.3%) |
| Grade 2 | 0 (0.0%) | 4 (1.9%) |
| Grade 3 | 2 (8.3%) | 6 (2.8%) |
|  |  |  |
| **Chills/shivering** | | |
| Normal | 7 (29.2%) | 86 (39.8%) |
| Grade 1 | 6 (25.0%) | 51 (23.6%) |
| Grade 2 | 11 (45.8%) | 73 (33.8%) |
| Grade 3 | 0 (0.0%) | 6 (2.8%) |
|  |  |  |
| **Myalgia (flu-like general muscle aches)** | | |
| Normal | 6 (25.0%) | 81 (37.5%) |
| Grade 1 | 8 (33.3%) | 64 (29.6%) |
| Grade 2 | 10 (41.7%) | 65 (30.1%) |
| Grade 3 | 0 (0.0%) | 6 (2.8%) |
|  |  |  |
| **Arthralgia** | | |
| Normal | 14 (58.3%) | 129 (59.7%) |
| Grade 1 | 6 (25.0%) | 52 (24.1%) |
| Grade 2 | 3 (12.5%) | 33 (15.3%) |
| Grade 3 | 1 (4.2%) | 2 (0.9%) |
|  |  |  |
| **Fatigue** | | |
| Normal | 6 (25.0%) | 57 (26.4%) |
| Grade 1 | 9 (37.5%) | 72 (33.3%) |
| Grade 2 | 8 (33.3%) | 74 (34.3%) |
| Grade 3 | 1 (4.2%) | 13 (6.0%) |
|  |  |  |
| **Headache** | | |
| Normal | 6 (25.0%) | 72 (33.3%) |
| Grade 1 | 10 (41.7%) | 68 (31.5%) |
| Grade 2 | 8 (33.3%) | 68 (31.5%) |
| Grade 3 | 0 (0.0%) | 8 (3.7%) |
|  |  |  |
| **Nausea** | | |
| Normal | 17 (70.8%) | 157 (72.7%) |
| Grade 1 | 6 (25.0%) | 42 (19.4%) |
| Grade 2 | 1 (4.2%) | 15 (6.9%) |
| Grade 3 | 0 (0.0%) | 2 (0.9%) |
|  |  |  |
| **Vomiting** | | |
| Normal | 24 (100.0%) | 206 (95.4%) |
| Grade 1 | 0 (0.0%) | 6 (2.8%) |
| Grade 2 | 0 (0.0%) | 4 (1.9%) |
|  |  |  |
| **Creatinine** | | |
| Normal | 24 (100.0%) | 212 (98.1%) |
| Grade 1 | 0 (0.0%) | 1 (0.5%) |
| Not known | 0 (0.0%) | 3 (1.4%) |
|  |  |  |
| **ALT** | | |
| Normal | 24 (100.0%) | 201 (93.1%) |
| Grade 1 | 0 (0.0%) | 12 (5.6%) |
| Not known | 0 (0.0%) | 3 (1.4%) |
|  |  |  |
| **AST** | | |
| Normal | 24 (100.0%) | 173 (80.1%) |
| Grade 1 | 0 (0.0%) | 4 (1.9%) |
| Not known | 0 (0.0%) | 39 (18.1%) |
|  |  |  |
| **Alkaline phosphate** | | |
| Normal | 24 (100.0%) | 213 (98.6%) |
| Not known | 0 (0.0%) | 3 (1.4%) |
|  |  |  |
| **Bilirubin** | | |
| Normal | 24 (100.0%) | 209 (96.8%) |
| Grade 1 | 0 (0.0%) | 4 (1.9%) |
| Not known | 0 (0.0%) | 3 (1.4%) |
|  |  |  |
| **Glucose (non-fasting) (high or low)** | | |
| Normal | 24 (100.0%) | 212 (98.1%) |
| Grade 2 | 0 (0.0%) | 1 (0.5%) |
| Not known | 0 (0.0%) | 3 (1.4%) |
|  |  |  |
| **Haemoglobin** | | |
| Normal | 24 (100.0%) | 212 (98.1%) |
| Grade 2 | 0 (0.0%) | 1 (0.5%) |
| Not known | 0 (0.0%) | 3 (1.4%) |
|  |  |  |
| **White blood cell count (high or low)** | | |
| Normal | 24 (100.0%) | 202 (93.5%) |
| Grade 1 | 0 (0.0%) | 11 (5.1%) |
| Not known | 0 (0.0%) | 3 (1.4%) |
|  |  |  |
| **Neutrophils** | | |
| Normal | 19 (79.2%) | 190 (88.0%) |
| Grade 1 | 4 (16.7%) | 20 (9.3%) |
| Grade 2 | 1 (4.2%) | 3 (1.4%) |
| Not known | 0 (0.0%) | 3 (1.4%) |
|  |  |  |
| **Lymphoctyes** | | |
| Normal | 24 (100.0%) | 207 (95.8%) |
| Grade 1 | 0 (0.0%) | 6 (2.8%) |
| Not known | 0 (0.0%) | 3 (1.4%) |
|  |  |  |
| **Platelets** | | |
| Normal | 24 (100.0%) | 212 (98.1%) |
| Grade 1 | 0 (0.0%) | 1 (0.5%) |
| Not known | 0 (0.0%) | 3 (1.4%) |

*Pollock et al, eClinicalMedicine, 2022. N.B. Dose-ranging cohort aged 18-45 years; expanded safety cohort 18-75 years

**One further participant received 1.0 µg followed by 1.0 µg, and did not experience any systemic reactions starting within 7 days of the second vaccine (AST not known; ALT normal)

## Figure 1 Solicited local injection site reactions starting within 7 days of the second vaccine for participants who received 1.0 µg followed by 10.0 µg

## Figure 2 Solicited systemic reactions that started within 7 days of second vaccine for participants who received 1.0 µg followed by 10.0 µg

## Table 6.7 Local injection site reactions starting within 7 days of the first vaccine by age at enrolment for participants who received 1.0 µg (dose-ranging cohort and expanded safety cohort)

|  | **18-39 N=117** | **40-59 N=69** | **60-75 N=78** | **Total N=264** |
| --- | --- | --- | --- | --- |
| **Any** | | | | |
| Normal | 32 (27.4%) | 34 (49.3%) | 48 (61.5%) | 114 (43.2%) |
| Grade 1 | 78 (66.7%) | 35 (50.7%) | 30 (38.5%) | 143 (54.2%) |
| Grade 2 | 7 (6.0%) | 0 (0.0%) | 0 (0.0%) | 7 (2.7%) |
|  |  |  |  |  |
| **Pain** | | | | |
| Normal | 79 (67.5%) | 56 (81.2%) | 71 (91.0%) | 206 (78.0%) |
| Grade 1 | 33 (28.2%) | 13 (18.8%) | 7 (9.0%) | 53 (20.1%) |
| Grade 2 | 5 (4.3%) | 0 (0.0%) | 0 (0.0%) | 5 (1.9%) |
|  |  |  |  |  |
| **Tenderness/discomfort** | | | | |
| Normal | 38 (32.5%) | 36 (52.2%) | 50 (64.1%) | 124 (47.0%) |
| Grade 1 | 73 (62.4%) | 33 (47.8%) | 28 (35.9%) | 134 (50.8%) |
| Grade 2 | 6 (5.1%) | 0 (0.0%) | 0 (0.0%) | 6 (2.3%) |
|  |  |  |  |  |
| **Erythema/redness** | | | | |
| Normal | 115 (98.3%) | 66 (95.7%) | 77 (98.7%) | 258 (97.7%) |
| Grade 1 | 2 (1.7%) | 3 (4.3%) | 1 (1.3%) | 6 (2.3%) |
|  |  |  |  |  |
| **Induration/swelling** | | | | |
| Normal | 117 (100.0%) | 68 (98.6%) | 78 (100.0%) | 263 (99.6%) |
| Grade 1 | 0 (0.0%) | 1 (1.4%) | 0 (0.0%) | 1 (0.4%) |

## Table 6.8 Systemic reactions starting within 7 days of the first vaccine by age at enrolment for participants who received 1.0 µg (dose-ranging cohort and expanded safety cohort)

|  | **18-39 N=117** | **40-59 N=69** | **60-75 N=78** | **Total N=264** |
| --- | --- | --- | --- | --- |
| **Any** | | | | |
| Normal | 41 (35.0%) | 29 (42.0%) | 42 (53.8%) | 112 (42.4%) |
| Grade 1 | 59 (50.4%) | 32 (46.4%) | 31 (39.7%) | 122 (46.2%) |
| Grade 2 | 17 (14.5%) | 7 (10.1%) | 5 (6.4%) | 29 (11.0%) |
| Grade 3 | 0 (0.0%) | 1 (1.4%) | 0 (0.0%) | 1 (0.4%) |
|  |  |  |  |  |
| **Temperature** | | | | |
| Normal | 116 (99.1%) | 69 (100.0%) | 78 (100.0%) | 263 (99.6%) |
| Grade 1 | 1 (0.9%) | 0 (0.0%) | 0 (0.0%) | 1 (0.4%) |
|  |  |  |  |  |
| **Chills/shivering** | | | | |
| Normal | 114 (97.4%) | 67 (97.1%) | 77 (98.7%) | 258 (97.7%) |
| Grade 1 | 3 (2.6%) | 1 (1.4%) | 0 (0.0%) | 4 (1.5%) |
| Grade 2 | 0 (0.0%) | 1 (1.4%) | 1 (1.3%) | 2 (0.8%) |
|  |  |  |  |  |
| **Myalgia (flu-like general muscle aches)** | | | | |
| Normal | 103 (88.0%) | 65 (94.2%) | 74 (94.9%) | 242 (91.7%) |
| Grade 1 | 10 (8.5%) | 4 (5.8%) | 3 (3.8%) | 17 (6.4%) |
| Grade 2 | 4 (3.4%) | 0 (0.0%) | 1 (1.3%) | 5 (1.9%) |
|  |  |  |  |  |
| **Arthralgia** | | | | |
| Normal | 108 (92.3%) | 66 (95.7%) | 71 (91.0%) | 245 (92.8%) |
| Grade 1 | 9 (7.7%) | 3 (4.3%) | 6 (7.7%) | 18 (6.8%) |
| Grade 2 | 0 (0.0%) | 0 (0.0%) | 1 (1.3%) | 1 (0.4%) |
|  |  |  |  |  |
| **Fatigue** | | | | |
| Normal | 71 (60.7%) | 54 (78.3%) | 64 (82.1%) | 189 (71.6%) |
| Grade 1 | 34 (29.1%) | 13 (18.8%) | 12 (15.4%) | 59 (22.3%) |
| Grade 2 | 12 (10.3%) | 2 (2.9%) | 2 (2.6%) | 16 (6.1%) |
|  |  |  |  |  |
| **Headache** | | | | |
| Normal | 78 (66.7%) | 46 (66.7%) | 61 (78.2%) | 185 (70.1%) |
| Grade 1 | 35 (29.9%) | 18 (26.1%) | 17 (21.8%) | 70 (26.5%) |
| Grade 2 | 4 (3.4%) | 5 (7.2%) | 0 (0.0%) | 9 (3.4%) |
|  |  |  |  |  |
| **Nausea** | | | | |
| Normal | 104 (88.9%) | 63 (91.3%) | 77 (98.7%) | 244 (92.4%) |
| Grade 1 | 11 (9.4%) | 5 (7.2%) | 1 (1.3%) | 17 (6.4%) |
| Grade 2 | 2 (1.7%) | 1 (1.4%) | 0 (0.0%) | 3 (1.1%) |
|  |  |  |  |  |
| **Vomiting** | | | | |
| Normal | 116 (99.1%) | 69 (100.0%) | 78 (100.0%) | 263 (99.6%) |
| Grade 1 | 1 (0.9%) | 0 (0.0%) | 0 (0.0%) | 1 (0.4%) |
|  |  |  |  |  |
| **Creatinine** | | | | |
| Normal | 115 (98.3%) | 64 (92.8%) | 78 (100.0%) | 257 (97.3%) |
| Grade 1 | 0 (0.0%) | 1 (1.4%) | 0 (0.0%) | 1 (0.4%) |
| Not known | 2 (1.7%) | 4 (5.8%) | 0 (0.0%) | 6 (2.3%) |
|  |  |  |  |  |
| **ALT** | | | | |
| Normal | 112 (95.7%) | 65 (94.2%) | 77 (98.7%) | 254 (96.2%) |
| Grade 1 | 2 (1.7%) | 0 (0.0%) | 1 (1.3%) | 3 (1.1%) |
| Not known | 3 (2.6%) | 4 (5.8%) | 0 (0.0%) | 7 (2.7%) |
|  |  |  |  |  |
| **AST** | | | | |
| Normal | 92 (78.6%) | 53 (76.8%) | 74 (94.9%) | 219 (83.0%) |
| Grade 1 | 1 (0.9%) | 1 (1.4%) | 2 (2.6%) | 4 (1.5%) |
| Not known | 24 (20.5%) | 15 (21.7%) | 2 (2.6%) | 41 (15.5%) |
|  |  |  |  |  |
| **Alkaline phosphate** | | | | |
| Normal | 114 (97.4%) | 65 (94.2%) | 78 (100.0%) | 257 (97.3%) |
| Not known | 3 (2.6%) | 4 (5.8%) | 0 (0.0%) | 7 (2.7%) |
|  |  |  |  |  |
| **Bilirubin** | | | | |
| Normal | 112 (95.7%) | 65 (94.2%) | 77 (98.7%) | 254 (96.2%) |
| Grade 1 | 2 (1.7%) | 0 (0.0%) | 1 (1.3%) | 3 (1.1%) |
| Not known | 3 (2.6%) | 4 (5.8%) | 0 (0.0%) | 7 (2.7%) |
|  |  |  |  |  |
| **Glucose (non-fasting) (high or low)** | | | | |
| Normal | 114 (97.4%) | 64 (92.8%) | 78 (100.0%) | 256 (97.0%) |
| Grade 1 | 1 (0.9%) | 0 (0.0%) | 0 (0.0%) | 1 (0.4%) |
| Grade 2 | 0 (0.0%) | 1 (1.4%) | 0 (0.0%) | 1 (0.4%) |
| Not known | 2 (1.7%) | 4 (5.8%) | 0 (0.0%) | 6 (2.3%) |
|  |  |  |  |  |
| **Haemoglobin** | | | | |
| Normal | 113 (96.6%) | 64 (92.8%) | 77 (98.7%) | 254 (96.2%) |
| Grade 1 | 2 (1.7%) | 1 (1.4%) | 1 (1.3%) | 4 (1.5%) |
| Not known | 2 (1.7%) | 4 (5.8%) | 0 (0.0%) | 6 (2.3%) |
|  |  |  |  |  |
| **White blood cell count (high or low)** | | | | |
| Normal | 112 (95.7%) | 61 (88.4%) | 75 (96.2%) | 248 (93.9%) |
| Grade 1 | 3 (2.6%) | 4 (5.8%) | 3 (3.8%) | 10 (3.8%) |
| Not known | 2 (1.7%) | 4 (5.8%) | 0 (0.0%) | 6 (2.3%) |
|  |  |  |  |  |
| **Neutrophils** | | | | |
| Normal | 107 (91.5%) | 60 (87.0%) | 74 (94.9%) | 241 (91.3%) |
| Grade 1 | 8 (6.8%) | 4 (5.8%) | 2 (2.6%) | 14 (5.3%) |
| Grade 2 | 0 (0.0%) | 0 (0.0%) | 2 (2.6%) | 2 (0.8%) |
| Grade 3 | 0 (0.0%) | 1 (1.4%) | 0 (0.0%) | 1 (0.4%) |
| Not known | 2 (1.7%) | 4 (5.8%) | 0 (0.0%) | 6 (2.3%) |
|  |  |  |  |  |
| **Lymphoctyes** | | | | |
| Normal | 113 (96.6%) | 65 (94.2%) | 72 (92.3%) | 250 (94.7%) |
| Grade 1 | 2 (1.7%) | 0 (0.0%) | 5 (6.4%) | 7 (2.7%) |
| Grade 2 | 0 (0.0%) | 0 (0.0%) | 1 (1.3%) | 1 (0.4%) |
| Not known | 2 (1.7%) | 4 (5.8%) | 0 (0.0%) | 6 (2.3%) |
|  |  |  |  |  |
| **Platelets** | | | | |
| Normal | 114 (97.4%) | 65 (94.2%) | 78 (100.0%) | 257 (97.3%) |
| Grade 2 | 1 (0.9%) | 0 (0.0%) | 0 (0.0%) | 1 (0.4%) |
| Not known | 2 (1.7%) | 4 (5.8%) | 0 (0.0%) | 6 (2.3%) |

## Table 6.9 Local injection site reactions starting within 7 days of the second vaccine by age at enrolment for participants who received 1.0 µg followed by 10.0 µg (expanded safety cohort only)

|  | **18-39 N=81** | **40-59 N=58** | **60-75 N=77** | **Total N=216** |
| --- | --- | --- | --- | --- |
| **Any** | | | | |
| Normal | 3 (3.7%) | 5 (8.6%) | 6 (7.8%) | 14 (6.5%) |
| Grade 1 | 39 (48.1%) | 39 (67.2%) | 52 (67.5%) | 130 (60.2%) |
| Grade 2 | 38 (46.9%) | 13 (22.4%) | 19 (24.7%) | 70 (32.4%) |
| Grade 3 | 1 (1.2%) | 1 (1.7%) | 0 (0.0%) | 2 (0.9%) |
|  |  |  |  |  |
| **Pain** | | | | |
| Normal | 15 (18.5%) | 21 (36.2%) | 32 (41.6%) | 68 (31.5%) |
| Grade 1 | 44 (54.3%) | 27 (46.6%) | 36 (46.8%) | 107 (49.5%) |
| Grade 2 | 22 (27.2%) | 9 (15.5%) | 9 (11.7%) | 40 (18.5%) |
| Grade 3 | 0 (0.0%) | 1 (1.7%) | 0 (0.0%) | 1 (0.5%) |
|  |  |  |  |  |
| **Tenderness/discomfort** | | | | |
| Normal | 4 (4.9%) | 8 (13.8%) | 10 (13.0%) | 22 (10.2%) |
| Grade 1 | 41 (50.6%) | 39 (67.2%) | 49 (63.6%) | 129 (59.7%) |
| Grade 2 | 35 (43.2%) | 11 (19.0%) | 18 (23.4%) | 64 (29.6%) |
| Grade 3 | 1 (1.2%) | 0 (0.0%) | 0 (0.0%) | 1 (0.5%) |
|  |  |  |  |  |
| **Erythema/redness** | | | | |
| Normal | 80 (98.8%) | 57 (98.3%) | 75 (97.4%) | 212 (98.1%) |
| Grade 1 | 1 (1.2%) | 1 (1.7%) | 1 (1.3%) | 3 (1.4%) |
| Grade 2 | 0 (0.0%) | 0 (0.0%) | 1 (1.3%) | 1 (0.5%) |
|  |  |  |  |  |
| **Induration/swelling** | | | | |
| Normal | 80 (98.8%) | 57 (98.3%) | 74 (96.1%) | 211 (97.7%) |
| Grade 1 | 1 (1.2%) | 1 (1.7%) | 3 (3.9%) | 5 (2.3%) |

## Table 6.10 Systemic reactions starting within 7 days of the second vaccine by age at enrolment for participants who received 1.0 µg followed by 10.0 µg (expanded safety cohort only)

|  | **18-39 N=81** | **40-59 N=58** | **60-75 N=77** | **Total N=216** |
| --- | --- | --- | --- | --- |
| **Any** | | | | |
| Normal | 2 (2.5%) | 11 (19.0%) | 12 (15.6%) | 25 (11.6%) |
| Grade 1 | 18 (22.2%) | 17 (29.3%) | 26 (33.8%) | 61 (28.2%) |
| Grade 2 | 49 (60.5%) | 23 (39.7%) | 34 (44.2%) | 106 (49.1%) |
| Grade 3 | 12 (14.8%) | 7 (12.1%) | 5 (6.5%) | 24 (11.1%) |
|  |  |  |  |  |
| **Temperature** | | | | |
| Normal | 67 (82.7%) | 51 (87.9%) | 70 (90.9%) | 188 (87.0%) |
| Grade 1 | 8 (9.9%) | 5 (8.6%) | 5 (6.5%) | 18 (8.3%) |
| Grade 2 | 2 (2.5%) | 1 (1.7%) | 1 (1.3%) | 4 (1.9%) |
| Grade 3 | 4 (4.9%) | 1 (1.7%) | 1 (1.3%) | 6 (2.8%) |
|  |  |  |  |  |
| **Chills/shivering** | | | | |
| Normal | 18 (22.2%) | 28 (48.3%) | 40 (51.9%) | 86 (39.8%) |
| Grade 1 | 22 (27.2%) | 15 (25.9%) | 14 (18.2%) | 51 (23.6%) |
| Grade 2 | 38 (46.9%) | 12 (20.7%) | 23 (29.9%) | 73 (33.8%) |
| Grade 3 | 3 (3.7%) | 3 (5.2%) | 0 (0.0%) | 6 (2.8%) |
|  |  |  |  |  |
| **Myalgia (flu-like general muscle aches)** | | | | |
| Normal | 16 (19.8%) | 26 (44.8%) | 39 (50.6%) | 81 (37.5%) |
| Grade 1 | 25 (30.9%) | 16 (27.6%) | 23 (29.9%) | 64 (29.6%) |
| Grade 2 | 37 (45.7%) | 13 (22.4%) | 15 (19.5%) | 65 (30.1%) |
| Grade 3 | 3 (3.7%) | 3 (5.2%) | 0 (0.0%) | 6 (2.8%) |
|  |  |  |  |  |
| **Arthralgia** | | | | |
| Normal | 43 (53.1%) | 42 (72.4%) | 44 (57.1%) | 129 (59.7%) |
| Grade 1 | 19 (23.5%) | 8 (13.8%) | 25 (32.5%) | 52 (24.1%) |
| Grade 2 | 18 (22.2%) | 7 (12.1%) | 8 (10.4%) | 33 (15.3%) |
| Grade 3 | 1 (1.2%) | 1 (1.7%) | 0 (0.0%) | 2 (0.9%) |
|  |  |  |  |  |
| **Fatigue** | | | | |
| Normal | 14 (17.3%) | 18 (31.0%) | 25 (32.5%) | 57 (26.4%) |
| Grade 1 | 22 (27.2%) | 21 (36.2%) | 29 (37.7%) | 72 (33.3%) |
| Grade 2 | 40 (49.4%) | 14 (24.1%) | 20 (26.0%) | 74 (34.3%) |
| Grade 3 | 5 (6.2%) | 5 (8.6%) | 3 (3.9%) | 13 (6.0%) |
|  |  |  |  |  |
| **Headache** | | | | |
| Normal | 12 (14.8%) | 26 (44.8%) | 34 (44.2%) | 72 (33.3%) |
| Grade 1 | 32 (39.5%) | 18 (31.0%) | 18 (23.4%) | 68 (31.5%) |
| Grade 2 | 32 (39.5%) | 13 (22.4%) | 23 (29.9%) | 68 (31.5%) |
| Grade 3 | 5 (6.2%) | 1 (1.7%) | 2 (2.6%) | 8 (3.7%) |
|  |  |  |  |  |
| **Nausea** | | | | |
| Normal | 48 (59.3%) | 44 (75.9%) | 65 (84.4%) | 157 (72.7%) |
| Grade 1 | 25 (30.9%) | 10 (17.2%) | 7 (9.1%) | 42 (19.4%) |
| Grade 2 | 8 (9.9%) | 3 (5.2%) | 4 (5.2%) | 15 (6.9%) |
| Grade 3 | 0 (0.0%) | 1 (1.7%) | 1 (1.3%) | 2 (0.9%) |
|  |  |  |  |  |
| **Vomiting** | | | | |
| Normal | 75 (92.6%) | 55 (94.8%) | 76 (98.7%) | 206 (95.4%) |
| Grade 1 | 4 (4.9%) | 1 (1.7%) | 1 (1.3%) | 6 (2.8%) |
| Grade 2 | 2 (2.5%) | 2 (3.4%) | 0 (0.0%) | 4 (1.9%) |
|  |  |  |  |  |
| **Creatinine** | | | | |
| Normal | 80 (98.8%) | 57 (98.3%) | 75 (97.4%) | 212 (98.1%) |
| Grade 1 | 1 (1.2%) | 0 (0.0%) | 0 (0.0%) | 1 (0.5%) |
| Not known | 0 (0.0%) | 1 (1.7%) | 2 (2.6%) | 3 (1.4%) |
|  |  |  |  |  |
| **ALT** | | | | |
| Normal | 78 (96.3%) | 54 (93.1%) | 69 (89.6%) | 201 (93.1%) |
| Grade 1 | 3 (3.7%) | 3 (5.2%) | 6 (7.8%) | 12 (5.6%) |
| Not known | 0 (0.0%) | 1 (1.7%) | 2 (2.6%) | 3 (1.4%) |
|  |  |  |  |  |
| **AST** | | | | |
| Normal | 57 (70.4%) | 45 (77.6%) | 71 (92.2%) | 173 (80.1%) |
| Grade 1 | 2 (2.5%) | 0 (0.0%) | 2 (2.6%) | 4 (1.9%) |
| Not known | 22 (27.2%) | 13 (22.4%) | 4 (5.2%) | 39 (18.1%) |
|  |  |  |  |  |
| **Alkaline phosphate** | | | | |
| Normal | 81 (100.0%) | 57 (98.3%) | 75 (97.4%) | 213 (98.6%) |
| Not known | 0 (0.0%) | 1 (1.7%) | 2 (2.6%) | 3 (1.4%) |
|  |  |  |  |  |
| **Bilirubin** | | | | |
| Normal | 78 (96.3%) | 57 (98.3%) | 74 (96.1%) | 209 (96.8%) |
| Grade 1 | 3 (3.7%) | 0 (0.0%) | 1 (1.3%) | 4 (1.9%) |
| Not known | 0 (0.0%) | 1 (1.7%) | 2 (2.6%) | 3 (1.4%) |
|  |  |  |  |  |
| **Glucose (non-fasting) (high or low)** | | | | |
| Normal | 81 (100.0%) | 56 (96.6%) | 75 (97.4%) | 212 (98.1%) |
| Grade 2 | 0 (0.0%) | 1 (1.7%) | 0 (0.0%) | 1 (0.5%) |
| Not known | 0 (0.0%) | 1 (1.7%) | 2 (2.6%) | 3 (1.4%) |
|  |  |  |  |  |
| **Haemoglobin** | | | | |
| Normal | 81 (100.0%) | 57 (98.3%) | 74 (96.1%) | 212 (98.1%) |
| Grade 2 | 0 (0.0%) | 0 (0.0%) | 1 (1.3%) | 1 (0.5%) |
| Not known | 0 (0.0%) | 1 (1.7%) | 2 (2.6%) | 3 (1.4%) |
|  |  |  |  |  |
| **White blood cell count (high or low)** | | | | |
| Normal | 76 (93.8%) | 53 (91.4%) | 73 (94.8%) | 202 (93.5%) |
| Grade 1 | 5 (6.2%) | 4 (6.9%) | 2 (2.6%) | 11 (5.1%) |
| Not known | 0 (0.0%) | 1 (1.7%) | 2 (2.6%) | 3 (1.4%) |
|  |  |  |  |  |
| **Neutrophils** | | | | |
| Normal | 70 (86.4%) | 50 (86.2%) | 70 (90.9%) | 190 (88.0%) |
| Grade 1 | 8 (9.9%) | 7 (12.1%) | 5 (6.5%) | 20 (9.3%) |
| Grade 2 | 3 (3.7%) | 0 (0.0%) | 0 (0.0%) | 3 (1.4%) |
| Not known | 0 (0.0%) | 1 (1.7%) | 2 (2.6%) | 3 (1.4%) |
|  |  |  |  |  |
| **Lymphoctyes** | | | | |
| Normal | 81 (100.0%) | 55 (94.8%) | 71 (92.2%) | 207 (95.8%) |
| Grade 1 | 0 (0.0%) | 2 (3.4%) | 4 (5.2%) | 6 (2.8%) |
| Not known | 0 (0.0%) | 1 (1.7%) | 2 (2.6%) | 3 (1.4%) |
|  |  |  |  |  |
| **Platelets** | | | | |
| Normal | 80 (98.8%) | 57 (98.3%) | 75 (97.4%) | 212 (98.1%) |
| Grade 1 | 1 (1.2%) | 0 (0.0%) | 0 (0.0%) | 1 (0.5%) |
| Not known | 0 (0.0%) | 1 (1.7%) | 2 (2.6%) | 3 (1.4%) |

## Table 6.11 Local injection site reactions starting within 7 days of the first vaccine by history of COVID-19 infection for participants who received 1.0 µg (dose-ranging cohort/expanded safety cohort)

|  | **No history of COVID-19 infection N=233** | **History of COVID-19 infection N=31** | **Total N=264** |
| --- | --- | --- | --- |
| **Any** |  |  |  |
| Normal | 102 (43.8%) | 12 (38.7%) | 114 (43.2%) |
| Grade 1 | 126 (54.1%) | 17 (54.8%) | 143 (54.2%) |
| Grade 2 | 5 (2.1%) | 2 (6.5%) | 7 (2.7%) |
|  |  |  |  |
| **Pain** |  |  |  |
| Normal | 186 (79.8%) | 20 (64.5%) | 206 (78.0%) |
| Grade 1 | 44 (18.9%) | 9 (29.0%) | 53 (20.1%) |
| Grade 2 | 3 (1.3%) | 2 (6.5%) | 5 (1.9%) |
|  |  |  |  |
| **Tenderness/discomfort** |  |  |  |
| Normal | 112 (48.1%) | 12 (38.7%) | 124 (47.0%) |
| Grade 1 | 116 (49.8%) | 18 (58.1%) | 134 (50.8%) |
| Grade 2 | 5 (2.1%) | 1 (3.2%) | 6 (2.3%) |
|  |  |  |  |
| **Erythema/redness** |  |  |  |
| Normal | 228 (97.9%) | 30 (96.8%) | 258 (97.7%) |
| Grade 1 | 5 (2.1%) | 1 (3.2%) | 6 (2.3%) |
|  |  |  |  |
| **Induration/swelling** |  |  |  |
| Normal | 232 (99.6%) | 31 (100.0%) | 263 (99.6%) |
| Grade 1 | 1 (0.4%) | 0 (0.0%) | 1 (0.4%) |

## Table 6.12 Systemic reactions starting within 7 days of the first vaccine by history of COVID-19 infection for participants who received 1.0 µg (dose-ranging cohort/expanded safety cohort)

|  | **No history of COVID-19 infection N=233** | **History of COVID-19 infection N=31** | **Total N=264** |
| --- | --- | --- | --- |
| **Any** | | | |
| Normal | 103 (44.2%) | 9 (29.0%) | 112 (42.4%) |
| Grade 1 | 103 (44.2%) | 19 (61.3%) | 122 (46.2%) |
| Grade 2 | 26 (11.2%) | 3 (9.7%) | 29 (11.0%) |
| Grade 3 | 1 (0.4%) | 0 (0.0%) | 1 (0.4%) |
|  |  |  |  |
| **Temperature** | | | |
| Normal | 232 (99.6%) | 31 (100.0%) | 263 (99.6%) |
| Grade 1 | 1 (0.4%) | 0 (0.0%) | 1 (0.4%) |
|  |  |  |  |
| **Chills/shivering** | | | |
| Normal | 229 (98.3%) | 29 (93.5%) | 258 (97.7%) |
| Grade 1 | 2 (0.9%) | 2 (6.5%) | 4 (1.5%) |
| Grade 2 | 2 (0.9%) | 0 (0.0%) | 2 (0.8%) |
|  |  |  |  |
| **Myalgia (flu-like general muscle aches)** | | | |
| Normal | 215 (92.3%) | 27 (87.1%) | 242 (91.7%) |
| Grade 1 | 14 (6.0%) | 3 (9.7%) | 17 (6.4%) |
| Grade 2 | 4 (1.7%) | 1 (3.2%) | 5 (1.9%) |
|  |  |  |  |
| **Arthralgia** | | | |
| Normal | 217 (93.1%) | 28 (90.3%) | 245 (92.8%) |
| Grade 1 | 15 (6.4%) | 3 (9.7%) | 18 (6.8%) |
| Grade 2 | 1 (0.4%) | 0 (0.0%) | 1 (0.4%) |
|  |  |  |  |
| **Fatigue** | | | |
| Normal | 169 (72.5%) | 20 (64.5%) | 189 (71.6%) |
| Grade 1 | 51 (21.9%) | 8 (25.8%) | 59 (22.3%) |
| Grade 2 | 13 (5.6%) | 3 (9.7%) | 16 (6.1%) |
|  |  |  |  |
| **Headache** | | | |
| Normal | 167 (71.7%) | 18 (58.1%) | 185 (70.1%) |
| Grade 1 | 59 (25.3%) | 11 (35.5%) | 70 (26.5%) |
| Grade 2 | 7 (3.0%) | 2 (6.5%) | 9 (3.4%) |
|  |  |  |  |
| **Nausea** | | | |
| Normal | 216 (92.7%) | 28 (90.3%) | 244 (92.4%) |
| Grade 1 | 15 (6.4%) | 2 (6.5%) | 17 (6.4%) |
| Grade 2 | 2 (0.9%) | 1 (3.2%) | 3 (1.1%) |
|  |  |  |  |
| **Vomiting** | | | |
| Normal | 232 (99.6%) | 31 (100.0%) | 263 (99.6%) |
| Grade 1 | 1 (0.4%) | 0 (0.0%) | 1 (0.4%) |
|  |  |  |  |
| **Creatinine** | | | |
| Normal | 228 (97.9%) | 29 (93.5%) | 257 (97.3%) |
| Grade 1 | 1 (0.4%) | 0 (0.0%) | 1 (0.4%) |
| Not known | 4 (1.7%) | 2 (6.5%) | 6 (2.3%) |
|  |  |  |  |
| **ALT** | | | |
| Normal | 226 (97.0%) | 28 (90.3%) | 254 (96.2%) |
| Grade 1 | 2 (0.9%) | 1 (3.2%) | 3 (1.1%) |
| Not known | 5 (2.1%) | 2 (6.5%) | 7 (2.7%) |
|  |  |  |  |
| **AST** | | | |
| Normal | 191 (82.0%) | 28 (90.3%) | 219 (83.0%) |
| Grade 1 | 4 (1.7%) | 0 (0.0%) | 4 (1.5%) |
| Not known | 38 (16.3%) | 3 (9.7%) | 41 (15.5%) |
|  |  |  |  |
| **Alkaline phosphate** | | | |
| Normal | 228 (97.9%) | 29 (93.5%) | 257 (97.3%) |
| Not known | 5 (2.1%) | 2 (6.5%) | 7 (2.7%) |
|  |  |  |  |
| **Bilirubin** | | | |
| Normal | 226 (97.0%) | 28 (90.3%) | 254 (96.2%) |
| Grade 1 | 2 (0.9%) | 1 (3.2%) | 3 (1.1%) |
| Not known | 5 (2.1%) | 2 (6.5%) | 7 (2.7%) |
|  |  |  |  |
| **Glucose (non-fasting) (high or low)** | | | |
| Normal | 227 (97.4%) | 29 (93.5%) | 256 (97.0%) |
| Grade 1 | 1 (0.4%) | 0 (0.0%) | 1 (0.4%) |
| Grade 2 | 1 (0.4%) | 0 (0.0%) | 1 (0.4%) |
| Not known | 4 (1.7%) | 2 (6.5%) | 6 (2.3%) |
|  |  |  |  |
| **Haemoglobin** | | | |
| Normal | 226 (97.0%) | 28 (90.3%) | 254 (96.2%) |
| Grade 1 | 3 (1.3%) | 1 (3.2%) | 4 (1.5%) |
| Not known | 4 (1.7%) | 2 (6.5%) | 6 (2.3%) |
|  |  |  |  |
| **White blood cell count (high or low)** | | | |
| Normal | 221 (94.8%) | 27 (87.1%) | 248 (93.9%) |
| Grade 1 | 8 (3.4%) | 2 (6.5%) | 10 (3.8%) |
| Not known | 4 (1.7%) | 2 (6.5%) | 6 (2.3%) |
|  |  |  |  |
| **Neutrophils** | | | |
| Normal | 214 (91.8%) | 27 (87.1%) | 241 (91.3%) |
| Grade 1 | 12 (5.2%) | 2 (6.5%) | 14 (5.3%) |
| Grade 2 | 2 (0.9%) | 0 (0.0%) | 2 (0.8%) |
| Grade 3 | 1 (0.4%) | 0 (0.0%) | 1 (0.4%) |
| Not known | 4 (1.7%) | 2 (6.5%) | 6 (2.3%) |
|  |  |  |  |
| **Lymphoctyes** | | | |
| Normal | 221 (94.8%) | 29 (93.5%) | 250 (94.7%) |
| Grade 1 | 7 (3.0%) | 0 (0.0%) | 7 (2.7%) |
| Grade 2 | 1 (0.4%) | 0 (0.0%) | 1 (0.4%) |
| Not known | 4 (1.7%) | 2 (6.5%) | 6 (2.3%) |
|  |  |  |  |
| **Platelets** | | | |
| Normal | 228 (97.9%) | 29 (93.5%) | 257 (97.3%) |
| Grade 2 | 1 (0.4%) | 0 (0.0%) | 1 (0.4%) |
| Not known | 4 (1.7%) | 2 (6.5%) | 6 (2.3%) |

## Table 6.13 Local injection site reactions starting within 7 days of the second vaccine by history of COVID-19 infection for participants who received 1.0 µg followed by 10.0 µg (expanded safety cohort)

|  | **No history of COVID-19 infection N=188** | **History of COVID-19 infection N=28** | **Total N=216** |
| --- | --- | --- | --- |
| **Any** | | | |
| Normal | 11 (5.9%) | 3 (10.7%) | 14 (6.5%) |
| Grade 1 | 116 (61.7%) | 14 (50.0%) | 130 (60.2%) |
| Grade 2 | 60 (31.9%) | 10 (35.7%) | 70 (32.4%) |
| Grade 3 | 1 (0.5%) | 1 (3.6%) | 2 (0.9%) |
|  |  |  |  |
| **Pain** | | | |
| Normal | 62 (33.0%) | 6 (21.4%) | 68 (31.5%) |
| Grade 1 | 93 (49.5%) | 14 (50.0%) | 107 (49.5%) |
| Grade 2 | 33 (17.6%) | 7 (25.0%) | 40 (18.5%) |
| Grade 3 | 0 (0.0%) | 1 (3.6%) | 1 (0.5%) |
|  |  |  |  |
| **Tenderness/discomfort** | | | |
| Normal | 18 (9.6%) | 4 (14.3%) | 22 (10.2%) |
| Grade 1 | 115 (61.2%) | 14 (50.0%) | 129 (59.7%) |
| Grade 2 | 54 (28.7%) | 10 (35.7%) | 64 (29.6%) |
| Grade 3 | 1 (0.5%) | 0 (0.0%) | 1 (0.5%) |
|  |  |  |  |
| **Erythema/redness** | | | |
| Normal | 184 (97.9%) | 28 (100.0%) | 212 (98.1%) |
| Grade 1 | 3 (1.6%) | 0 (0.0%) | 3 (1.4%) |
| Grade 2 | 1 (0.5%) | 0 (0.0%) | 1 (0.5%) |
|  |  |  |  |
| **Induration/swelling** | | | |
| Normal | 183 (97.3%) | 28 (100.0%) | 211 (97.7%) |
| Grade 1 | 5 (2.7%) | 0 (0.0%) | 5 (2.3%) |

## Table 6.14 Systemic reactions starting within 7 days of the second vaccine by history of COVID-19 infection for participants who received 1.0 µg followed by 10.0 µg (expanded safety cohort)

|  | **No history of COVID-19 infection N=188** | **History of COVID-19 infection N=28** | **Total N=216** |
| --- | --- | --- | --- |
| **Any** | | | |
| Normal | 21 (11.2%) | 4 (14.3%) | 25 (11.6%) |
| Grade 1 | 57 (30.3%) | 4 (14.3%) | 61 (28.2%) |
| Grade 2 | 91 (48.4%) | 15 (53.6%) | 106 (49.1%) |
| Grade 3 | 19 (10.1%) | 5 (17.9%) | 24 (11.1%) |
|  |  |  |  |
| **Temperature** | | | |
| Normal | 165 (87.8%) | 23 (82.1%) | 188 (87.0%) |
| Grade 1 | 15 (8.0%) | 3 (10.7%) | 18 (8.3%) |
| Grade 2 | 3 (1.6%) | 1 (3.6%) | 4 (1.9%) |
| Grade 3 | 5 (2.7%) | 1 (3.6%) | 6 (2.8%) |
|  |  |  |  |
| **Chills/shivering** | | | |
| Normal | 77 (41.0%) | 9 (32.1%) | 86 (39.8%) |
| Grade 1 | 45 (23.9%) | 6 (21.4%) | 51 (23.6%) |
| Grade 2 | 62 (33.0%) | 11 (39.3%) | 73 (33.8%) |
| Grade 3 | 4 (2.1%) | 2 (7.1%) | 6 (2.8%) |
|  |  |  |  |
| **Myalgia (flu-like general muscle aches)** | | | |
| Normal | 72 (38.3%) | 9 (32.1%) | 81 (37.5%) |
| Grade 1 | 55 (29.3%) | 9 (32.1%) | 64 (29.6%) |
| Grade 2 | 57 (30.3%) | 8 (28.6%) | 65 (30.1%) |
| Grade 3 | 4 (2.1%) | 2 (7.1%) | 6 (2.8%) |
|  |  |  |  |
| **Arthralgia** | | | |
| Normal | 112 (59.6%) | 17 (60.7%) | 129 (59.7%) |
| Grade 1 | 45 (23.9%) | 7 (25.0%) | 52 (24.1%) |
| Grade 2 | 30 (16.0%) | 3 (10.7%) | 33 (15.3%) |
| Grade 3 | 1 (0.5%) | 1 (3.6%) | 2 (0.9%) |
|  |  |  |  |
| **Fatigue** | | | |
| Normal | 48 (25.5%) | 9 (32.1%) | 57 (26.4%) |
| Grade 1 | 69 (36.7%) | 3 (10.7%) | 72 (33.3%) |
| Grade 2 | 62 (33.0%) | 12 (42.9%) | 74 (34.3%) |
| Grade 3 | 9 (4.8%) | 4 (14.3%) | 13 (6.0%) |
|  |  |  |  |
| **Headache** | | | |
| Normal | 64 (34.0%) | 8 (28.6%) | 72 (33.3%) |
| Grade 1 | 57 (30.3%) | 11 (39.3%) | 68 (31.5%) |
| Grade 2 | 62 (33.0%) | 6 (21.4%) | 68 (31.5%) |
| Grade 3 | 5 (2.7%) | 3 (10.7%) | 8 (3.7%) |
|  |  |  |  |
| **Nausea** | | | |
| Normal | 145 (77.1%) | 12 (42.9%) | 157 (72.7%) |
| Grade 1 | 31 (16.5%) | 11 (39.3%) | 42 (19.4%) |
| Grade 2 | 12 (6.4%) | 3 (10.7%) | 15 (6.9%) |
| Grade 3 | 0 (0.0%) | 2 (7.1%) | 2 (0.9%) |
|  |  |  |  |
| **Vomiting** | | | |
| Normal | 182 (96.8%) | 24 (85.7%) | 206 (95.4%) |
| Grade 1 | 2 (1.1%) | 4 (14.3%) | 6 (2.8%) |
| Grade 2 | 4 (2.1%) | 0 (0.0%) | 4 (1.9%) |
|  |  |  |  |
| **Creatinine** | | | |
| Normal | 184 (97.9%) | 28 (100.0%) | 212 (98.1%) |
| Grade 1 | 1 (0.5%) | 0 (0.0%) | 1 (0.5%) |
| Not known | 3 (1.6%) | 0 (0.0%) | 3 (1.4%) |
|  |  |  |  |
| **ALT** | | | |
| Normal | 175 (93.1%) | 26 (92.9%) | 201 (93.1%) |
| Grade 1 | 10 (5.3%) | 2 (7.1%) | 12 (5.6%) |
| Not known | 3 (1.6%) | 0 (0.0%) | 3 (1.4%) |
|  |  |  |  |
| **AST** | | | |
| Normal | 146 (77.7%) | 27 (96.4%) | 173 (80.1%) |
| Grade 1 | 4 (2.1%) | 0 (0.0%) | 4 (1.9%) |
| Not known | 38 (20.2%) | 1 (3.6%) | 39 (18.1%) |
|  |  |  |  |
| **Alkaline phosphate** | | | |
| Normal | 185 (98.4%) | 28 (100.0%) | 213 (98.6%) |
| Not known | 3 (1.6%) | 0 (0.0%) | 3 (1.4%) |
|  |  |  |  |
| **Bilirubin** | | | |
| Normal | 182 (96.8%) | 27 (96.4%) | 209 (96.8%) |
| Grade 1 | 3 (1.6%) | 1 (3.6%) | 4 (1.9%) |
| Not known | 3 (1.6%) | 0 (0.0%) | 3 (1.4%) |
|  |  |  |  |
| **Glucose (non-fasting) (high or low)** | | | |
| Normal | 184 (97.9%) | 28 (100.0%) | 212 (98.1%) |
| Grade 2 | 1 (0.5%) | 0 (0.0%) | 1 (0.5%) |
| Not known | 3 (1.6%) | 0 (0.0%) | 3 (1.4%) |
|  |  |  |  |
| **Haemoglobin** | | | |
| Normal | 184 (97.9%) | 28 (100.0%) | 212 (98.1%) |
| Grade 2 | 1 (0.5%) | 0 (0.0%) | 1 (0.5%) |
| Not known | 3 (1.6%) | 0 (0.0%) | 3 (1.4%) |
|  |  |  |  |
| **White blood cell count (high or low)** | | | |
| Normal | 177 (94.1%) | 25 (89.3%) | 202 (93.5%) |
| Grade 1 | 8 (4.3%) | 3 (10.7%) | 11 (5.1%) |
| Not known | 3 (1.6%) | 0 (0.0%) | 3 (1.4%) |
|  |  |  |  |
| **Neutrophils** | | | |
| Normal | 167 (88.8%) | 23 (82.1%) | 190 (88.0%) |
| Grade 1 | 17 (9.0%) | 3 (10.7%) | 20 (9.3%) |
| Grade 2 | 1 (0.5%) | 2 (7.1%) | 3 (1.4%) |
| Not known | 3 (1.6%) | 0 (0.0%) | 3 (1.4%) |
|  |  |  |  |
| **Lymphoctyes** | | | |
| Normal | 179 (95.2%) | 28 (100.0%) | 207 (95.8%) |
| Grade 1 | 6 (3.2%) | 0 (0.0%) | 6 (2.8%) |
| Not known | 3 (1.6%) | 0 (0.0%) | 3 (1.4%) |
|  |  |  |  |
| **Platelets** | | | |
| Normal | 184 (97.9%) | 28 (100.0%) | 212 (98.1%) |
| Grade 1 | 1 (0.5%) | 0 (0.0%) | 1 (0.5%) |
| Not known | 3 (1.6%) | 0 (0.0%) | 3 (1.4%) |

## Table 6.15 Other adverse events (AEs), regardless of relationship to IMP

|  | **Expanded safety cohort N=222** |
| --- | --- |
| **ANY** | |
| Not reported | 21 (9.5%) |
| Grade 1 | 100 (45.0%) |
| Grade 2 | 89 (40.1%) |
| Grade 3 | 9 (4.1%) |
| Grade 4 | 3 (1.4%) |
|  |  |
| **INFECTIONS AND INFESTATIONS** | |
| **Any** | |
| Not reported | 172 (77.5%) |
| Grade 1 | 26 (11.7%) |
| Grade 2 | 21 (9.5%) |
| Grade 3 | 3 (1.4%) |
|  |  |
| **Cellulitis** | |
| Not reported | 221 (99.5%) |
| Grade 2 | 1 (0.5%) |
|  |  |
| **Conjunctivitis** | |
| Not reported | 221 (99.5%) |
| Grade 1 | 1 (0.5%) |
|  |  |
| **Ear infection** | |
| Not reported | 220 (99.1%) |
| Grade 1 | 2 (0.9%) |
|  |  |
| **Eczema herpeticum** | |
| Not reported | 221 (99.5%) |
| Grade 2 | 1 (0.5%) |
|  |  |
| **Eye infection** | |
| Not reported | 221 (99.5%) |
| Grade 1 | 1 (0.5%) |
|  |  |
| **Gastroenteritis** | |
| Not reported | 221 (99.5%) |
| Grade 2 | 1 (0.5%) |
|  |  |
| **Genitourinary chlamydia infection** | |
| Not reported | 221 (99.5%) |
| Grade 1 | 1 (0.5%) |
|  |  |
| **Hordeolum** | |
| Not reported | 221 (99.5%) |
| Grade 1 | 1 (0.5%) |
|  |  |
| **Localised infection** | |
| Not reported | 221 (99.5%) |
| Grade 2 | 1 (0.5%) |
|  |  |
| **Nasopharyngitis** | |
| Not reported | 210 (94.6%) |
| Grade 1 | 12 (5.4%) |
|  |  |
| **Otitis externa** | |
| Not reported | 221 (99.5%) |
| Grade 2 | 1 (0.5%) |
|  |  |
| **Pyelonephritis** | |
| Not reported | 221 (99.5%) |
| Grade 1 | 1 (0.5%) |
|  |  |
| **Rhinitis** | |
| Not reported | 221 (99.5%) |
| Grade 1 | 1 (0.5%) |
|  |  |
| **Sinusitis** | |
| Not reported | 221 (99.5%) |
| Grade 2 | 1 (0.5%) |
|  |  |
| **Tonsillitis** | |
| Not reported | 221 (99.5%) |
| Grade 3 | 1 (0.5%) |
|  |  |
| **Upper respiratory tract infection** | |
| Not reported | 220 (99.1%) |
| Grade 1 | 1 (0.5%) |
| Grade 2 | 1 (0.5%) |
|  |  |
| **Urinary tract infection** | |
| Not reported | 217 (97.7%) |
| Grade 1 | 2 (0.9%) |
| Grade 2 | 2 (0.9%) |
| Grade 3 | 1 (0.5%) |
|  |  |
| **Viral tonsillitis** | |
| Not reported | 220 (99.1%) |
| Grade 2 | 2 (0.9%) |
|  |  |
| **Vulvovaginal candidiasis** | |
| Not reported | 221 (99.5%) |
| Grade 1 | 1 (0.5%) |
|  |  |
| **Pneumonia bacterial** | |
| Not reported | 221 (99.5%) |
| Grade 3 | 1 (0.5%) |
|  |  |
| **Arthritis infective** | |
| Not reported | 221 (99.5%) |
| Grade 2 | 1 (0.5%) |
|  |  |
| **Oral herpes** | |
| Not reported | 219 (98.6%) |
| Grade 1 | 2 (0.9%) |
| Grade 2 | 1 (0.5%) |
|  |  |
| **Post procedural infection** | |
| Not reported | 221 (99.5%) |
| Grade 2 | 1 (0.5%) |
|  |  |
| **Root canal infection** | |
| Not reported | 221 (99.5%) |
| Grade 2 | 1 (0.5%) |
|  |  |
| **COVID-19** | |
| Not reported | 209 (94.1%) |
| Grade 1 | 3 (1.4%) |
| Grade 2 | 9 (4.1%) |
| Grade 3 | 1 (0.5%) |
|  |  |
| **Suspected COVID-19** | |
| Not reported | 221 (99.5%) |
| Grade 1 | 1 (0.5%) |
|  |  |
| **NEOPLASMS BENIGN, MALIGNANT AND UNSPECIFIED (INCL CYSTS AND POLYPS)** | |
| **Any** | |
| Not reported | 221 (99.5%) |
| Grade 3 | 1 (0.5%) |
|  |  |
| **Parathyroid tumour benign** | |
| Not reported | 221 (99.5%) |
| Grade 3 | 1 (0.5%) |
|  |  |
| **BLOOD AND LYMPHATIC SYSTEM DISORDERS** | |
| **Any** | |
| Not reported | 205 (92.3%) |
| Grade 1 | 14 (6.3%) |
| Grade 2 | 3 (1.4%) |
|  |  |
| **Anaemia** | |
| Not reported | 218 (98.2%) |
| Grade 1 | 4 (1.8%) |
|  |  |
| **Eosinophilia** | |
| Not reported | 221 (99.5%) |
| Grade 1 | 1 (0.5%) |
|  |  |
| **Leukocytosis** | |
| Not reported | 221 (99.5%) |
| Grade 1 | 1 (0.5%) |
|  |  |
| **Lymphadenopathy** | |
| Not reported | 217 (97.7%) |
| Grade 1 | 5 (2.3%) |
|  |  |
| **Lymphopenia** | |
| Not reported | 221 (99.5%) |
| Grade 1 | 1 (0.5%) |
|  |  |
| **Neutropenia** | |
| Not reported | 218 (98.2%) |
| Grade 1 | 2 (0.9%) |
| Grade 2 | 2 (0.9%) |
|  |  |
| **Thrombocytosis** | |
| Not reported | 221 (99.5%) |
| Grade 1 | 1 (0.5%) |
|  |  |
| **Monoclonal B-cell lymphocytosis** | |
| Not reported | 221 (99.5%) |
| Grade 2 | 1 (0.5%) |
|  |  |
| **IMMUNE SYSTEM DISORDERS** | |
| **Any** | |
| Not reported | 221 (99.5%) |
| Grade 1 | 1 (0.5%) |
|  |  |
| **Seasonal allergy** | |
| Not reported | 221 (99.5%) |
| Grade 1 | 1 (0.5%) |
|  |  |
| **METABOLISM AND NUTRITION DISORDERS** | |
| **Any** | |
| Not reported | 213 (95.9%) |
| Grade 1 | 4 (1.8%) |
| Grade 2 | 5 (2.3%) |
|  |  |
| **Hyperglycaemia** | |
| Not reported | 216 (97.3%) |
| Grade 1 | 3 (1.4%) |
| Grade 2 | 3 (1.4%) |
|  |  |
| **Hypoglycaemia** | |
| Not reported | 221 (99.5%) |
| Grade 2 | 1 (0.5%) |
|  |  |
| **Decreased appetite** | |
| Not reported | 220 (99.1%) |
| Grade 1 | 1 (0.5%) |
| Grade 2 | 1 (0.5%) |
|  |  |
| **PSYCHIATRIC DISORDERS** | |
| **Any** | |
| Not reported | 219 (98.6%) |
| Grade 1 | 2 (0.9%) |
| Grade 2 | 1 (0.5%) |
|  |  |
| **Anxiety** | |
| Not reported | 221 (99.5%) |
| Grade 1 | 1 (0.5%) |
|  |  |
| **Panic attack** | |
| Not reported | 221 (99.5%) |
| Grade 2 | 1 (0.5%) |
|  |  |
| **Mental fatigue** | |
| Not reported | 221 (99.5%) |
| Grade 1 | 1 (0.5%) |
|  |  |
| **NERVOUS SYSTEM DISORDERS** | |
| **Any** | |
| Not reported | 174 (78.4%) |
| Grade 1 | 34 (15.3%) |
| Grade 2 | 12 (5.4%) |
| Grade 3 | 2 (0.9%) |
|  |  |
| **Anosmia** | |
| Not reported | 220 (99.1%) |
| Grade 1 | 1 (0.5%) |
| Grade 2 | 1 (0.5%) |
|  |  |
| **Clumsiness** | |
| Not reported | 221 (99.5%) |
| Grade 1 | 1 (0.5%) |
|  |  |
| **Dizziness** | |
| Not reported | 217 (97.7%) |
| Grade 1 | 3 (1.4%) |
| Grade 2 | 2 (0.9%) |
|  |  |
| **Headache** | |
| Not reported | 187 (84.2%) |
| Grade 1 | 26 (11.7%) |
| Grade 2 | 8 (3.6%) |
| Grade 3 | 1 (0.5%) |
|  |  |
| **Hypoaesthesia** | |
| Not reported | 221 (99.5%) |
| Grade 1 | 1 (0.5%) |
|  |  |
| **Lethargy** | |
| Not reported | 221 (99.5%) |
| Grade 1 | 1 (0.5%) |
|  |  |
| **Loss of consciousness** | |
| Not reported | 221 (99.5%) |
| Grade 1 | 1 (0.5%) |
|  |  |
| **Migraine** | |
| Not reported | 220 (99.1%) |
| Grade 1 | 2 (0.9%) |
|  |  |
| **Migraine with aura** | |
| Not reported | 221 (99.5%) |
| Grade 2 | 1 (0.5%) |
|  |  |
| **Parosmia** | |
| Not reported | 221 (99.5%) |
| Grade 1 | 1 (0.5%) |
|  |  |
| **Sinus headache** | |
| Not reported | 221 (99.5%) |
| Grade 2 | 1 (0.5%) |
|  |  |
| **Syncope** | |
| Not reported | 221 (99.5%) |
| Grade 3 | 1 (0.5%) |
|  |  |
| **Tension headache** | |
| Not reported | 221 (99.5%) |
| Grade 1 | 1 (0.5%) |
|  |  |
| **EYE DISORDERS** | |
| **Any** | |
| Not reported | 218 (98.2%) |
| Grade 1 | 2 (0.9%) |
| Grade 2 | 2 (0.9%) |
|  |  |
| **Eye swelling** | |
| Not reported | 221 (99.5%) |
| Grade 2 | 1 (0.5%) |
|  |  |
| **Glaucoma** | |
| Not reported | 221 (99.5%) |
| Grade 1 | 1 (0.5%) |
|  |  |
| **Iritis** | |
| Not reported | 221 (99.5%) |
| Grade 2 | 1 (0.5%) |
|  |  |
| **Eye pruritus** | |
| Not reported | 221 (99.5%) |
| Grade 1 | 1 (0.5%) |
|  |  |
| **EAR AND LABYRINTH DISORDERS** | |
| **Any** | |
| Not reported | 218 (98.2%) |
| Grade 1 | 2 (0.9%) |
| Grade 2 | 2 (0.9%) |
|  |  |
| **Ear pain** | |
| Not reported | 220 (99.1%) |
| Grade 1 | 1 (0.5%) |
| Grade 2 | 1 (0.5%) |
|  |  |
| **Tinnitus** | |
| Not reported | 221 (99.5%) |
| Grade 1 | 1 (0.5%) |
|  |  |
| **Vertigo positional** | |
| Not reported | 221 (99.5%) |
| Grade 2 | 1 (0.5%) |
|  |  |
| **CARDIAC DISORDERS** | |
| **Any** | |
| Not reported | 219 (98.6%) |
| Grade 1 | 1 (0.5%) |
| Grade 2 | 1 (0.5%) |
| Grade 4 | 1 (0.5%) |
|  |  |
| **Myocardial ischaemia** | |
| Not reported | 221 (99.5%) |
| Grade 4 | 1 (0.5%) |
|  |  |
| **Palpitations** | |
| Not reported | 220 (99.1%) |
| Grade 1 | 1 (0.5%) |
| Grade 2 | 1 (0.5%) |
|  |  |
| **VASCULAR DISORDERS** | |
| **Any** | |
| Not reported | 216 (97.3%) |
| Grade 1 | 3 (1.4%) |
| Grade 2 | 3 (1.4%) |
|  |  |
| **Diastolic hypertension** | |
| Not reported | 221 (99.5%) |
| Grade 2 | 1 (0.5%) |
|  |  |
| **Hypertension** | |
| Not reported | 220 (99.1%) |
| Grade 1 | 2 (0.9%) |
|  |  |
| **Peripheral coldness** | |
| Not reported | 221 (99.5%) |
| Grade 2 | 1 (0.5%) |
|  |  |
| **Systolic hypertension** | |
| Not reported | 221 (99.5%) |
| Grade 1 | 1 (0.5%) |
|  |  |
| **Vascular pain** | |
| Not reported | 221 (99.5%) |
| Grade 2 | 1 (0.5%) |
|  |  |
| **RESPIRATORY, THORACIC AND MEDIASTINAL DISORDERS** | |
| **Any** | |
| Not reported | 203 (91.4%) |
| Grade 1 | 16 (7.2%) |
| Grade 2 | 3 (1.4%) |
|  |  |
| **Asthma** | |
| Not reported | 221 (99.5%) |
| Grade 2 | 1 (0.5%) |
|  |  |
| **Cough** | |
| Not reported | 218 (98.2%) |
| Grade 1 | 2 (0.9%) |
| Grade 2 | 2 (0.9%) |
|  |  |
| **Dry throat** | |
| Not reported | 220 (99.1%) |
| Grade 1 | 2 (0.9%) |
|  |  |
| **Nasal congestion** | |
| Not reported | 219 (98.6%) |
| Grade 1 | 2 (0.9%) |
| Grade 2 | 1 (0.5%) |
|  |  |
| **Productive cough** | |
| Not reported | 220 (99.1%) |
| Grade 1 | 2 (0.9%) |
|  |  |
| **Rhinorrhoea** | |
| Not reported | 220 (99.1%) |
| Grade 1 | 2 (0.9%) |
|  |  |
| **Allergic cough** | |
| Not reported | 220 (99.1%) |
| Grade 1 | 2 (0.9%) |
|  |  |
| **Oropharyngeal pain** | |
| Not reported | 213 (95.9%) |
| Grade 1 | 8 (3.6%) |
| Grade 2 | 1 (0.5%) |
|  |  |
| **GASTROINTESTINAL DISORDERS** | |
| **Any** | |
| Not reported | 188 (84.7%) |
| Grade 1 | 28 (12.6%) |
| Grade 2 | 6 (2.7%) |
|  |  |
| **Abdominal discomfort** | |
| Not reported | 220 (99.1%) |
| Grade 1 | 2 (0.9%) |
|  |  |
| **Abdominal distension** | |
| Not reported | 221 (99.5%) |
| Grade 1 | 1 (0.5%) |
|  |  |
| **Abdominal pain** | |
| Not reported | 218 (98.2%) |
| Grade 1 | 3 (1.4%) |
| Grade 2 | 1 (0.5%) |
|  |  |
| **Abdominal pain lower** | |
| Not reported | 221 (99.5%) |
| Grade 2 | 1 (0.5%) |
|  |  |
| **Diarrhoea** | |
| Not reported | 211 (95.0%) |
| Grade 1 | 8 (3.6%) |
| Grade 2 | 3 (1.4%) |
|  |  |
| **Diarrhoea haemorrhagic** | |
| Not reported | 221 (99.5%) |
| Grade 2 | 1 (0.5%) |
|  |  |
| **Dyspepsia** | |
| Not reported | 220 (99.1%) |
| Grade 1 | 2 (0.9%) |
|  |  |
| **Gastrooesophageal reflux disease** | |
| Not reported | 220 (99.1%) |
| Grade 1 | 2 (0.9%) |
|  |  |
| **Haematochezia** | |
| Not reported | 221 (99.5%) |
| Grade 1 | 1 (0.5%) |
|  |  |
| **Hiatus hernia** | |
| Not reported | 221 (99.5%) |
| Grade 1 | 1 (0.5%) |
|  |  |
| **Lip oedema** | |
| Not reported | 221 (99.5%) |
| Grade 1 | 1 (0.5%) |
|  |  |
| **Mouth ulceration** | |
| Not reported | 218 (98.2%) |
| Grade 1 | 4 (1.8%) |
|  |  |
| **Nausea** | |
| Not reported | 220 (99.1%) |
| Grade 1 | 2 (0.9%) |
|  |  |
| **Oral pain** | |
| Not reported | 221 (99.5%) |
| Grade 1 | 1 (0.5%) |
|  |  |
| **Rectal haemorrhage** | |
| Not reported | 221 (99.5%) |
| Grade 1 | 1 (0.5%) |
|  |  |
| **Toothache** | |
| Not reported | 218 (98.2%) |
| Grade 1 | 4 (1.8%) |
|  |  |
| **Vomiting** | |
| Not reported | 220 (99.1%) |
| Grade 2 | 2 (0.9%) |
|  |  |
| **Oral dysaesthesia** | |
| Not reported | 221 (99.5%) |
| Grade 1 | 1 (0.5%) |
|  |  |
| **Hypoaesthesia oral** | |
| Not reported | 221 (99.5%) |
| Grade 1 | 1 (0.5%) |
|  |  |
| **HEPATOBILIARY DISORDERS** | |
| **Any** | |
| Not reported | 221 (99.5%) |
| Grade 1 | 1 (0.5%) |
|  |  |
| **Cholelithiasis** | |
| Not reported | 221 (99.5%) |
| Grade 1 | 1 (0.5%) |
|  |  |
| **SKIN AND SUBCUTANEOUS TISSUE DISORDERS** | |
| **Any** | |
| Not reported | 205 (92.3%) |
| Grade 1 | 12 (5.4%) |
| Grade 2 | 5 (2.3%) |
|  |  |
| **Blister** | |
| Not reported | 221 (99.5%) |
| Grade 1 | 1 (0.5%) |
|  |  |
| **Dermatitis contact** | |
| Not reported | 221 (99.5%) |
| Grade 2 | 1 (0.5%) |
|  |  |
| **Dry skin** | |
| Not reported | 221 (99.5%) |
| Grade 1 | 1 (0.5%) |
|  |  |
| **Eczema** | |
| Not reported | 220 (99.1%) |
| Grade 1 | 1 (0.5%) |
| Grade 2 | 1 (0.5%) |
|  |  |
| **Erythema** | |
| Not reported | 221 (99.5%) |
| Grade 1 | 1 (0.5%) |
|  |  |
| **Night sweats** | |
| Not reported | 220 (99.1%) |
| Grade 1 | 2 (0.9%) |
|  |  |
| **Pruritus** | |
| Not reported | 219 (98.6%) |
| Grade 1 | 2 (0.9%) |
| Grade 2 | 1 (0.5%) |
|  |  |
| **Psoriasis** | |
| Not reported | 221 (99.5%) |
| Grade 1 | 1 (0.5%) |
|  |  |
| **Rash** | |
| Not reported | 219 (98.6%) |
| Grade 1 | 2 (0.9%) |
| Grade 2 | 1 (0.5%) |
|  |  |
| **Rash macular** | |
| Not reported | 221 (99.5%) |
| Grade 1 | 1 (0.5%) |
|  |  |
| **Skin ulcer** | |
| Not reported | 221 (99.5%) |
| Grade 2 | 1 (0.5%) |
|  |  |
| **MUSCULOSKELETAL AND CONNECTIVE TISSUE DISORDERS** | |
| **Any** | |
| Not reported | 183 (82.4%) |
| Grade 1 | 21 (9.5%) |
| Grade 2 | 16 (7.2%) |
| Grade 3 | 1 (0.5%) |
| Grade 4 | 1 (0.5%) |
|  |  |
| **Arthralgia** | |
| Not reported | 217 (97.7%) |
| Grade 1 | 4 (1.8%) |
| Grade 2 | 1 (0.5%) |
|  |  |
| **Arthritis** | |
| Not reported | 221 (99.5%) |
| Grade 2 | 1 (0.5%) |
|  |  |
| **Back pain** | |
| Not reported | 210 (94.6%) |
| Grade 1 | 7 (3.2%) |
| Grade 2 | 4 (1.8%) |
| Grade 4 | 1 (0.5%) |
|  |  |
| **Costochondritis** | |
| Not reported | 221 (99.5%) |
| Grade 2 | 1 (0.5%) |
|  |  |
| **Musculoskeletal pain** | |
| Not reported | 221 (99.5%) |
| Grade 1 | 1 (0.5%) |
|  |  |
| **Myalgia** | |
| Not reported | 209 (94.1%) |
| Grade 1 | 7 (3.2%) |
| Grade 2 | 6 (2.7%) |
|  |  |
| **Osteoarthritis** | |
| Not reported | 220 (99.1%) |
| Grade 1 | 1 (0.5%) |
| Grade 3 | 1 (0.5%) |
|  |  |
| **Osteoporosis** | |
| Not reported | 221 (99.5%) |
| Grade 2 | 1 (0.5%) |
|  |  |
| **Pain in extremity** | |
| Not reported | 218 (98.2%) |
| Grade 1 | 4 (1.8%) |
|  |  |
| **Periarthritis** | |
| Not reported | 220 (99.1%) |
| Grade 1 | 1 (0.5%) |
| Grade 2 | 1 (0.5%) |
|  |  |
| **Intervertebral disc protrusion** | |
| Not reported | 221 (99.5%) |
| Grade 2 | 1 (0.5%) |
|  |  |
| **Musculoskeletal chest pain** | |
| Not reported | 221 (99.5%) |
| Grade 1 | 1 (0.5%) |
|  |  |
| **Foot deformity** | |
| Not reported | 221 (99.5%) |
| Grade 2 | 1 (0.5%) |
|  |  |
| **RENAL AND URINARY DISORDERS** | |
| **Any** | |
| Not reported | 219 (98.6%) |
| Grade 1 | 2 (0.9%) |
| Grade 2 | 1 (0.5%) |
|  |  |
| **Nephrolithiasis** | |
| Not reported | 220 (99.1%) |
| Grade 1 | 1 (0.5%) |
| Grade 2 | 1 (0.5%) |
|  |  |
| **Urethral haemorrhage** | |
| Not reported | 221 (99.5%) |
| Grade 1 | 1 (0.5%) |
|  |  |
| **REPRODUCTIVE SYSTEM AND BREAST DISORDERS** | |
| **Any** | |
| Not reported | 218 (98.2%) |
| Grade 1 | 3 (1.4%) |
| Grade 2 | 1 (0.5%) |
|  |  |
| **Breast cyst** | |
| Not reported | 221 (99.5%) |
| Grade 2 | 1 (0.5%) |
|  |  |
| **Dysmenorrhoea** | |
| Not reported | 220 (99.1%) |
| Grade 1 | 2 (0.9%) |
|  |  |
| **Adenomyosis** | |
| Not reported | 221 (99.5%) |
| Grade 1 | 1 (0.5%) |
|  |  |
| **GENERAL DISORDERS AND ADMINISTRATION SITE CONDITIONS** | |
| **Any** | |
| Not reported | 133 (59.9%) |
| Grade 1 | 70 (31.5%) |
| Grade 2 | 19 (8.6%) |
|  |  |
| **Chest pain** | |
| Not reported | 219 (98.6%) |
| Grade 1 | 3 (1.4%) |
|  |  |
| **Chills** | |
| Not reported | 216 (97.3%) |
| Grade 1 | 4 (1.8%) |
| Grade 2 | 2 (0.9%) |
|  |  |
| **Fatigue** | |
| Not reported | 200 (90.1%) |
| Grade 1 | 15 (6.8%) |
| Grade 2 | 7 (3.2%) |
|  |  |
| **Feeling abnormal** | |
| Not reported | 221 (99.5%) |
| Grade 1 | 1 (0.5%) |
|  |  |
| **Feeling hot** | |
| Not reported | 220 (99.1%) |
| Grade 1 | 2 (0.9%) |
|  |  |
| **Hypothermia** | |
| Not reported | 221 (99.5%) |
| Grade 2 | 1 (0.5%) |
|  |  |
| **Influenza like illness** | |
| Not reported | 195 (87.8%) |
| Grade 1 | 21 (9.5%) |
| Grade 2 | 6 (2.7%) |
|  |  |
| **Injection site bruising** | |
| Not reported | 221 (99.5%) |
| Grade 1 | 1 (0.5%) |
|  |  |
| **Injection site pain** | |
| Not reported | 191 (86.0%) |
| Grade 1 | 27 (12.2%) |
| Grade 2 | 4 (1.8%) |
|  |  |
| **Malaise** | |
| Not reported | 221 (99.5%) |
| Grade 1 | 1 (0.5%) |
|  |  |
| **Pain** | |
| Not reported | 221 (99.5%) |
| Grade 1 | 1 (0.5%) |
|  |  |
| **Pyrexia** | |
| Not reported | 216 (97.3%) |
| Grade 1 | 4 (1.8%) |
| Grade 2 | 2 (0.9%) |
|  |  |
| **Tenderness** | |
| Not reported | 220 (99.1%) |
| Grade 1 | 2 (0.9%) |
|  |  |
| **Injection site discomfort** | |
| Not reported | 214 (96.4%) |
| Grade 1 | 8 (3.6%) |
|  |  |
| **Feeling of body temperature change** | |
| Not reported | 221 (99.5%) |
| Grade 1 | 1 (0.5%) |
|  |  |
| **Vaccination site pain** | |
| Not reported | 216 (97.3%) |
| Grade 1 | 4 (1.8%) |
| Grade 2 | 2 (0.9%) |
|  |  |
| **Vaccination site discolouration** | |
| Not reported | 221 (99.5%) |
| Grade 1 | 1 (0.5%) |
|  |  |
| **Vaccination site discomfort** | |
| Not reported | 220 (99.1%) |
| Grade 1 | 2 (0.9%) |
|  |  |
| **Vaccination site bruising** | |
| Not reported | 217 (97.7%) |
| Grade 1 | 4 (1.8%) |
| Grade 2 | 1 (0.5%) |
|  |  |
| **Vaccination site swelling** | |
| Not reported | 221 (99.5%) |
| Grade 1 | 1 (0.5%) |
|  |  |
| **Vaccination site movement impairment** | |
| Not reported | 221 (99.5%) |
| Grade 1 | 1 (0.5%) |
|  |  |
| **Facial discomfort** | |
| Not reported | 221 (99.5%) |
| Grade 1 | 1 (0.5%) |
|  |  |
| **INVESTIGATIONS** | |
| **Any** | |
| Not reported | 107 (48.2%) |
| Grade 1 | 84 (37.8%) |
| Grade 2 | 29 (13.1%) |
| Grade 3 | 2 (0.9%) |
|  |  |
| **Alanine aminotransferase increased** | |
| Not reported | 192 (86.5%) |
| Grade 1 | 28 (12.6%) |
| Grade 2 | 2 (0.9%) |
|  |  |
| **Aspartate aminotransferase increased** | |
| Not reported | 203 (91.4%) |
| Grade 1 | 14 (6.3%) |
| Grade 2 | 5 (2.3%) |
|  |  |
| **Blood bilirubin increased** | |
| Not reported | 209 (94.1%) |
| Grade 1 | 13 (5.9%) |
|  |  |
| **Blood calcium increased** | |
| Not reported | 221 (99.5%) |
| Grade 1 | 1 (0.5%) |
|  |  |
| **Blood creatinine increased** | |
| Not reported | 220 (99.1%) |
| Grade 1 | 2 (0.9%) |
|  |  |
| **Blood glucose decreased** | |
| Not reported | 217 (97.7%) |
| Grade 1 | 2 (0.9%) |
| Grade 2 | 3 (1.4%) |
|  |  |
| **Blood glucose increased** | |
| Not reported | 206 (92.8%) |
| Grade 1 | 11 (5.0%) |
| Grade 2 | 5 (2.3%) |
|  |  |
| **Body temperature increased** | |
| Not reported | 221 (99.5%) |
| Grade 2 | 1 (0.5%) |
|  |  |
| **Haemoglobin decreased** | |
| Not reported | 208 (93.7%) |
| Grade 1 | 13 (5.9%) |
| Grade 2 | 1 (0.5%) |
|  |  |
| **Lymphocyte count decreased** | |
| Not reported | 190 (85.6%) |
| Grade 1 | 30 (13.5%) |
| Grade 2 | 2 (0.9%) |
|  |  |
| **Lymphocyte count increased** | |
| Not reported | 221 (99.5%) |
| Grade 2 | 1 (0.5%) |
|  |  |
| **Neutrophil count decreased** | |
| Not reported | 183 (82.4%) |
| Grade 1 | 28 (12.6%) |
| Grade 2 | 9 (4.1%) |
| Grade 3 | 2 (0.9%) |
|  |  |
| **Platelet count decreased** | |
| Not reported | 219 (98.6%) |
| Grade 2 | 3 (1.4%) |
|  |  |
| **White blood cell count decreased** | |
| Not reported | 200 (90.1%) |
| Grade 1 | 21 (9.5%) |
| Grade 2 | 1 (0.5%) |
|  |  |
| **White blood cell count increased** | |
| Not reported | 213 (95.9%) |
| Grade 1 | 8 (3.6%) |
| Grade 2 | 1 (0.5%) |
|  |  |
| **Transaminases increased** | |
| Not reported | 220 (99.1%) |
| Grade 1 | 2 (0.9%) |
|  |  |
| **Blood alkaline phosphatase increased** | |
| Not reported | 219 (98.6%) |
| Grade 1 | 3 (1.4%) |
|  |  |
| **INJURY, POISONING AND PROCEDURAL COMPLICATIONS** | |
| **Any** | |
| Not reported | 191 (86.0%) |
| Grade 1 | 20 (9.0%) |
| Grade 2 | 10 (4.5%) |
| Grade 4 | 1 (0.5%) |
|  |  |
| **Arthropod bite** | |
| Not reported | 217 (97.7%) |
| Grade 1 | 5 (2.3%) |
|  |  |
| **Arthropod sting** | |
| Not reported | 221 (99.5%) |
| Grade 1 | 1 (0.5%) |
|  |  |
| **Chillblains** | |
| Not reported | 221 (99.5%) |
| Grade 2 | 1 (0.5%) |
|  |  |
| **Fall** | |
| Not reported | 221 (99.5%) |
| Grade 1 | 1 (0.5%) |
|  |  |
| **Foreign body in eye** | |
| Not reported | 221 (99.5%) |
| Grade 1 | 1 (0.5%) |
|  |  |
| **Ligament sprain** | |
| Not reported | 220 (99.1%) |
| Grade 1 | 2 (0.9%) |
|  |  |
| **Rib fracture** | |
| Not reported | 221 (99.5%) |
| Grade 2 | 1 (0.5%) |
|  |  |
| **Soft tissue injury** | |
| Not reported | 221 (99.5%) |
| Grade 2 | 1 (0.5%) |
|  |  |
| **Stress fracture** | |
| Not reported | 221 (99.5%) |
| Grade 2 | 1 (0.5%) |
|  |  |
| **Tibia fracture** | |
| Not reported | 221 (99.5%) |
| Grade 4 | 1 (0.5%) |
|  |  |
| **Muscle strain** | |
| Not reported | 220 (99.1%) |
| Grade 1 | 2 (0.9%) |
|  |  |
| **Contusion** | |
| Not reported | 220 (99.1%) |
| Grade 1 | 2 (0.9%) |
|  |  |
| **Skin laceration** | |
| Not reported | 221 (99.5%) |
| Grade 2 | 1 (0.5%) |
|  |  |
| **Joint injury** | |
| Not reported | 220 (99.1%) |
| Grade 1 | 1 (0.5%) |
| Grade 2 | 1 (0.5%) |
|  |  |
| **Upper limb fracture** | |
| Not reported | 221 (99.5%) |
| Grade 2 | 1 (0.5%) |
|  |  |
| **Skin abrasion** | |
| Not reported | 221 (99.5%) |
| Grade 1 | 1 (0.5%) |
|  |  |
| **Procedural dizziness** | |
| Not reported | 221 (99.5%) |
| Grade 1 | 1 (0.5%) |
|  |  |
| **Injection related reaction** | |
| Not reported | 214 (96.4%) |
| Grade 1 | 5 (2.3%) |
| Grade 2 | 3 (1.4%) |
|  |  |
| **Post procedural hypotension** | |
| Not reported | 221 (99.5%) |
| Grade 1 | 1 (0.5%) |
|  |  |
| **SURGICAL AND MEDICAL PROCEDURES** | |
| **Any** | |
| Not reported | 215 (96.8%) |
| Grade 1 | 3 (1.4%) |
| Grade 2 | 3 (1.4%) |
| Grade 3 | 1 (0.5%) |
|  |  |
| **Varicocele repair** | |
| Not reported | 221 (99.5%) |
| Grade 3 | 1 (0.5%) |
|  |  |
| **Endodontic procedure** | |
| Not reported | 220 (99.1%) |
| Grade 1 | 2 (0.9%) |
|  |  |
| **Prophylaxis against HIV infection** | |
| Not reported | 221 (99.5%) |
| Grade 2 | 1 (0.5%) |
|  |  |
| **Dental care** | |
| Not reported | 221 (99.5%) |
| Grade 2 | 1 (0.5%) |
|  |  |
| **Dental operation** | |
| Not reported | 221 (99.5%) |
| Grade 2 | 1 (0.5%) |
|  |  |
| **Tooth extraction** | |
| Not reported | 221 (99.5%) |
| Grade 1 | 1 (0.5%) |

## Table 6.16 Other adverse reactions (ARs)

|  | **Expanded safety cohort N=222** |
| --- | --- |
| **ANY** | |
| Not reported | 137 (61.7%) |
| Grade 1 | 61 (27.5%) |
| Grade 2 | 24 (10.8%) |
|  |  |
| **INFECTIONS AND INFESTATIONS** | |
| **Any** | |
| Not reported | 218 (98.2%) |
| Grade 1 | 3 (1.4%) |
| Grade 2 | 1 (0.5%) |
|  |  |
| **Eczema herpeticum** | |
| Not reported | 221 (99.5%) |
| Grade 2 | 1 (0.5%) |
|  |  |
| **Nasopharyngitis** | |
| Not reported | 220 (99.1%) |
| Grade 1 | 2 (0.9%) |
|  |  |
| **Rhinitis** | |
| Not reported | 221 (99.5%) |
| Grade 1 | 1 (0.5%) |
|  |  |
| **BLOOD AND LYMPHATIC SYSTEM DISORDERS** | |
| **Any** | |
| Not reported | 215 (96.8%) |
| Grade 1 | 6 (2.7%) |
| Grade 2 | 1 (0.5%) |
|  |  |
| **Lymphadenopathy** | |
| Not reported | 218 (98.2%) |
| Grade 1 | 4 (1.8%) |
|  |  |
| **Lymphopenia** | |
| Not reported | 221 (99.5%) |
| Grade 1 | 1 (0.5%) |
|  |  |
| **Neutropenia** | |
| Not reported | 220 (99.1%) |
| Grade 1 | 1 (0.5%) |
| Grade 2 | 1 (0.5%) |
|  |  |
| **METABOLISM AND NUTRITION DISORDERS** | |
| **Any** | |
| Not reported | 220 (99.1%) |
| Grade 1 | 2 (0.9%) |
|  |  |
| **Decreased appetite** | |
| Not reported | 220 (99.1%) |
| Grade 1 | 2 (0.9%) |
|  |  |
| **PSYCHIATRIC DISORDERS** | |
| **Any** | |
| Not reported | 221 (99.5%) |
| Grade 1 | 1 (0.5%) |
|  |  |
| **Mental fatigue** | |
| Not reported | 221 (99.5%) |
| Grade 1 | 1 (0.5%) |
|  |  |
| **NERVOUS SYSTEM DISORDERS** | |
| **Any** | |
| Not reported | 209 (94.1%) |
| Grade 1 | 9 (4.1%) |
| Grade 2 | 4 (1.8%) |
|  |  |
| **Clumsiness** | |
| Not reported | 221 (99.5%) |
| Grade 1 | 1 (0.5%) |
|  |  |
| **Dizziness** | |
| Not reported | 218 (98.2%) |
| Grade 1 | 2 (0.9%) |
| Grade 2 | 2 (0.9%) |
|  |  |
| **Headache** | |
| Not reported | 216 (97.3%) |
| Grade 1 | 4 (1.8%) |
| Grade 2 | 2 (0.9%) |
|  |  |
| **Hypoaesthesia** | |
| Not reported | 221 (99.5%) |
| Grade 1 | 1 (0.5%) |
|  |  |
| **Lethargy** | |
| Not reported | 221 (99.5%) |
| Grade 1 | 1 (0.5%) |
|  |  |
| **CARDIAC DISORDERS** | |
| **Any** | |
| Not reported | 221 (99.5%) |
| Grade 2 | 1 (0.5%) |
|  |  |
| **Palpitations** | |
| Not reported | 221 (99.5%) |
| Grade 2 | 1 (0.5%) |
|  |  |
| **VASCULAR DISORDERS** | |
| **Any** | |
| Not reported | 221 (99.5%) |
| Grade 2 | 1 (0.5%) |
|  |  |
| **Peripheral coldness** | |
| Not reported | 221 (99.5%) |
| Grade 2 | 1 (0.5%) |
|  |  |
| **RESPIRATORY, THORACIC AND MEDIASTINAL DISORDERS** | |
| **Any** | |
| Not reported | 217 (97.7%) |
| Grade 1 | 5 (2.3%) |
|  |  |
| **Cough** | |
| Not reported | 221 (99.5%) |
| Grade 1 | 1 (0.5%) |
|  |  |
| **Dry throat** | |
| Not reported | 221 (99.5%) |
| Grade 1 | 1 (0.5%) |
|  |  |
| **Rhinorrhoea** | |
| Not reported | 221 (99.5%) |
| Grade 1 | 1 (0.5%) |
|  |  |
| **Oropharyngeal pain** | |
| Not reported | 219 (98.6%) |
| Grade 1 | 3 (1.4%) |
|  |  |
| **GASTROINTESTINAL DISORDERS** | |
| **Any** | |
| Not reported | 210 (94.6%) |
| Grade 1 | 9 (4.1%) |
| Grade 2 | 3 (1.4%) |
|  |  |
| **Abdominal discomfort** | |
| Not reported | 221 (99.5%) |
| Grade 1 | 1 (0.5%) |
|  |  |
| **Abdominal distension** | |
| Not reported | 221 (99.5%) |
| Grade 1 | 1 (0.5%) |
|  |  |
| **Abdominal pain** | |
| Not reported | 221 (99.5%) |
| Grade 2 | 1 (0.5%) |
|  |  |
| **Diarrhoea** | |
| Not reported | 216 (97.3%) |
| Grade 1 | 3 (1.4%) |
| Grade 2 | 3 (1.4%) |
|  |  |
| **Lip oedema** | |
| Not reported | 221 (99.5%) |
| Grade 1 | 1 (0.5%) |
|  |  |
| **Mouth ulceration** | |
| Not reported | 221 (99.5%) |
| Grade 1 | 1 (0.5%) |
|  |  |
| **Oral pain** | |
| Not reported | 221 (99.5%) |
| Grade 1 | 1 (0.5%) |
|  |  |
| **Vomiting** | |
| Not reported | 221 (99.5%) |
| Grade 2 | 1 (0.5%) |
|  |  |
| **Oral dysaesthesia** | |
| Not reported | 221 (99.5%) |
| Grade 1 | 1 (0.5%) |
|  |  |
| **Hypoaesthesia oral** | |
| Not reported | 221 (99.5%) |
| Grade 1 | 1 (0.5%) |
|  |  |
| **SKIN AND SUBCUTANEOUS TISSUE DISORDERS** | |
| **Any** | |
| Not reported | 217 (97.7%) |
| Grade 1 | 3 (1.4%) |
| Grade 2 | 2 (0.9%) |
|  |  |
| **Erythema** | |
| Not reported | 221 (99.5%) |
| Grade 1 | 1 (0.5%) |
|  |  |
| **Night sweats** | |
| Not reported | 221 (99.5%) |
| Grade 1 | 1 (0.5%) |
|  |  |
| **Rash** | |
| Not reported | 221 (99.5%) |
| Grade 2 | 1 (0.5%) |
|  |  |
| **Rash macular** | |
| Not reported | 221 (99.5%) |
| Grade 1 | 1 (0.5%) |
|  |  |
| **Skin ulcer** | |
| Not reported | 221 (99.5%) |
| Grade 2 | 1 (0.5%) |
|  |  |
| **MUSCULOSKELETAL AND CONNECTIVE TISSUE DISORDERS** | |
| **Any** | |
| Not reported | 215 (96.8%) |
| Grade 1 | 4 (1.8%) |
| Grade 2 | 3 (1.4%) |
|  |  |
| **Back pain** | |
| Not reported | 219 (98.6%) |
| Grade 1 | 1 (0.5%) |
| Grade 2 | 2 (0.9%) |
|  |  |
| **Myalgia** | |
| Not reported | 218 (98.2%) |
| Grade 1 | 3 (1.4%) |
| Grade 2 | 1 (0.5%) |
|  |  |
| **GENERAL DISORDERS AND ADMINISTRATION SITE CONDITIONS** | |
| **Any** | |
| Not reported | 178 (80.2%) |
| Grade 1 | 35 (15.8%) |
| Grade 2 | 9 (4.1%) |
|  |  |
| **Fatigue** | |
| Not reported | 216 (97.3%) |
| Grade 1 | 3 (1.4%) |
| Grade 2 | 3 (1.4%) |
|  |  |
| **Feeling abnormal** | |
| Not reported | 221 (99.5%) |
| Grade 1 | 1 (0.5%) |
|  |  |
| **Feeling hot** | |
| Not reported | 221 (99.5%) |
| Grade 1 | 1 (0.5%) |
|  |  |
| **Hypothermia** | |
| Not reported | 221 (99.5%) |
| Grade 2 | 1 (0.5%) |
|  |  |
| **Influenza like illness** | |
| Not reported | 201 (90.5%) |
| Grade 1 | 18 (8.1%) |
| Grade 2 | 3 (1.4%) |
|  |  |
| **Malaise** | |
| Not reported | 221 (99.5%) |
| Grade 1 | 1 (0.5%) |
|  |  |
| **Pyrexia** | |
| Not reported | 220 (99.1%) |
| Grade 1 | 2 (0.9%) |
|  |  |
| **Tenderness** | |
| Not reported | 220 (99.1%) |
| Grade 1 | 2 (0.9%) |
|  |  |
| **Vaccination site pain** | |
| Not reported | 217 (97.7%) |
| Grade 1 | 3 (1.4%) |
| Grade 2 | 2 (0.9%) |
|  |  |
| **Vaccination site discolouration** | |
| Not reported | 221 (99.5%) |
| Grade 1 | 1 (0.5%) |
|  |  |
| **Vaccination site discomfort** | |
| Not reported | 220 (99.1%) |
| Grade 1 | 2 (0.9%) |
|  |  |
| **Vaccination site bruising** | |
| Not reported | 217 (97.7%) |
| Grade 1 | 4 (1.8%) |
| Grade 2 | 1 (0.5%) |
|  |  |
| **Vaccination site swelling** | |
| Not reported | 221 (99.5%) |
| Grade 1 | 1 (0.5%) |
|  |  |
| **Vaccination site movement impairment** | |
| Not reported | 221 (99.5%) |
| Grade 1 | 1 (0.5%) |
|  |  |
| **Facial discomfort** | |
| Not reported | 221 (99.5%) |
| Grade 1 | 1 (0.5%) |
|  |  |
| **INVESTIGATIONS** | |
| **Any** | |
| Not reported | 199 (89.6%) |
| Grade 1 | 19 (8.6%) |
| Grade 2 | 4 (1.8%) |
|  |  |
| **Alanine aminotransferase increased** | |
| Not reported | 217 (97.7%) |
| Grade 1 | 4 (1.8%) |
| Grade 2 | 1 (0.5%) |
|  |  |
| **Aspartate aminotransferase increased** | |
| Not reported | 217 (97.7%) |
| Grade 1 | 4 (1.8%) |
| Grade 2 | 1 (0.5%) |
|  |  |
| **Blood bilirubin increased** | |
| Not reported | 220 (99.1%) |
| Grade 1 | 2 (0.9%) |
|  |  |
| **Lymphocyte count decreased** | |
| Not reported | 218 (98.2%) |
| Grade 1 | 3 (1.4%) |
| Grade 2 | 1 (0.5%) |
|  |  |
| **Neutrophil count decreased** | |
| Not reported | 211 (95.0%) |
| Grade 1 | 9 (4.1%) |
| Grade 2 | 2 (0.9%) |
|  |  |
| **White blood cell count decreased** | |
| Not reported | 216 (97.3%) |
| Grade 1 | 6 (2.7%) |
|  |  |
| **White blood cell count increased** | |
| Not reported | 221 (99.5%) |
| Grade 1 | 1 (0.5%) |

## Figures 3-13 Change in laboratory safety parameters

Actual change from baseline values 7 and 14 days after the first vaccine; on the day of second vaccine (pre-vaccination); 7, 14, 28 and 56 days after the second vaccine; and at day 364.

Figure 3. Changes in creatinine

Figure 4. Changes in ALT

Figure 5. Changes in AST

Figure 6. Changes in alkaline phosphatase

Figure 7. Changes in bilirubin

Figure 8. Changes in glucose

Figure 9. Changes in haemoglobin

Figure 10. Changes in white cell count

Figure 11. Changes in neutrophils

Figure 12. Changes in lymphocytes

Figure 13. Changes in platelets

Figure 14. Pseudoneutralising antibodies (IC50) against and Omicron BA.5 from a subsection of participants receiving two doses of LNP-saRNA in the expanded safety cohort

**

Pseudoneutralisation titres (NT50) at 2 weeks post 2^nd^ vaccination against WT v Omicron BA.5 SARS-CoV-2. The data are from 60 COVAC1 participants who had received two doses of LNP-saRNA in the expanded safety cohort (1µg followed by 10µg). The data are split into individuals who were seronegative to SARS-CoV-2 at baseline (turquoise [WT] and pink [Omicron BA.5] circles) and individuals who were seropositive at baseline (red [WT] and yellow [Omicron BA.5] squares). The values indicate the number of individuals who returned a neutralisation titre above LOQ.

# Table 7. Adjusted analysis for comparison for 1/10 ug versus 10/19 ug cohorts

|  | Odds ratio for seroconversion (1/10 vs 10/10) [95% CI] | Fold-difference in titre  (1/10 vs 10/10) [95% CI] |
| --- | --- | --- |
| Unadjusted | 2.63 (1.05,6.54) | 2.46 (1.12, 4.81) |
| Adjusted for age | 3.05 (1.13,8.26) | 2.75 (1.35, 5.56) |

# Figure 15 Individual immune responses in baseline seropositive participants in the expanded safety cohort

Dotted lines represent the day the second vaccine was administered (or due,but missed).

Participant plots 25-27 show sero-reversion after the first vaccine followed by a return to baseline or higher at the next assessment on the day of second vaccine; participant plots 28-30 did not receive a second vaccine; participant 31 did not attend for the sample collection.
