# Supplementary material for: COVAC1 phase 2a expanded safety and immunogenicity study of a self-amplifying RNA vaccine against SARS-CoV-2
Source: eClinicalMedicine. 2023 Jan 13;56:101823. doi: 10.1016/j.eclinm.2022.101823 (PMC9837478; doi:10.1016/j.eclinm.2022.101823)
Supplement: Appendix 1 Protocol v8 [file mmc3.pdf]

COVAC1

A first-in-human clinical trial to assess the safety and immunogenicity of a self-amplifying ribonucleic acid (saRNA) vaccine encoding the S glycoprotein of SARS-CoV-2, the causative agent of COVID-19

Version: 8.0
Date: 15 Dec 2020

EUDRACT NO.: 2020-001646-20
IRAS ID: 279315

Authorised by:
Name: Kat Pollock
Role: Chief Investigator
Signature:
Date: 15 Dec 2020

Name: David Dunn
Role: Trial Statistician
Signature:
Date: 15 Dec 2020

This protocol has been produced using MRC CTU at UCL Protocol Template version 8.0. The template, but not any study-specific content, is licensed under a Creative Commons Attribution 4.0 International License (https://creativecommons.org/licenses/by/4.0/). Use of the template in production of other protocols is allowed, but MRC CTU at UCL must be credited.

GENERAL INFORMATION

This document was constructed using the MRC CTU at UCL Protocol Template Version 8.0. The CTU endorses the Standard Protocol Items: Recommendations For Interventional Trials (SPIRIT) initiative. It describes the COVAC1 trial, coordinated by the Medical Research Council (MRC) Clinical Trials Unit (CTU) at University College London (UCL), and provides information about procedures for entering participants into it. The protocol should not be used as an aide-memoire or guide for the treatment of other patients. Every care has been taken in drafting this protocol, but corrections or amendments may be necessary. These will be circulated to the registered investigators in the trial, but sites entering participants for the first time are advised to contact the trial team to confirm they have the most up-to-date version.

COMPLIANCE

The trial will be conducted in compliance with the approved protocol, the Declaration of Helsinki 1996, the principles of Good Clinical Practice (GCP) as laid down by the ICH topic E6 (R2), Commission Clinical Trials Directive 2005/28/EC* with the implementation in national legislation in the UK by Statutory Instrument 2004/1031 and subsequent amendments, General Data Protection Regulation and the UK Data Protection Act 2018 (DPA number: Z6364106), and the UK Policy Framework for Health and Social Care Research.

*Until the Clinical Trials Regulation EU No 536/2014 becomes applicable, the trial will be conducted in accordance with the Clinical Trials Directive as implemented in the UK statutory instrument. When the directive is repealed on the day of entry into application of the Clinical Trial Regulation the trial will work towards implementation of the Regulation (536/2014) following any transition period.

SPONSOR

Imperial College London (ICL) is the trial Sponsor and has delegated responsibility for the overall management of the COVAC1 trial to the MRC CTU at UCL. Queries relating to ICL sponsorship of this trial should be addressed to the Head of Regulatory Compliance at:

Joint Research Compliance Office
Imperial College London and Imperial College Healthcare NHS Trust
Room 215, Level 2, Medical School Building
Norfolk Place
London, W2 1PG
Tel: 020 7594 9480
http://www3.imperial.ac.uk/clinicalresearchgovernanceoffice.

FUNDING

The trial is funded by the MRC, via UK Research and Innovation.

AUTHORISATIONS AND APPROVALS

This trial was approved by the NIHR Urgent Public Health Group and is, therefore, part of the NIHR Clinical Research Network portfolio.

TRIAL REGISTRATION

This trial has be registered with the ISRCTN register: ISRCTN17072692.

RANDOMISATIONS
Relevant to the evaluation of 0.1, 0.3 and 1 µg doses at the Imperial CRF site only; via manual list at site

SAE REPORTING
Please report all SAEs and Notable Events via the eDC system within 24 hours of becoming aware of an SAE/NE
If you have any issues with entering the SAE/NE or have any questions please email mrcctu.covac@ucl.ac.uk

TRIAL ADMINISTRATION

Please direct all queries to the MRC CTU Trial Managers at the Coordinating Site in the first instance; clinical queries will be passed to the Chief Investigator via the Coordinating Site.

COORDINATING SITE

MRC Clinical Trials Unit at UCL	Switchboard:	020 7670 4700
Institute of Clinical Trials & Methodology<br>90 High Holborn 2nd Floor<br>London WC1V 6LJ	Fax:	020 7670 4659
	Email:	mrcctu.covac@ucl.ac.uk

MRC CTU AT UCL AND IMPERIAL COLLEGE LONDON COORDINATING STAFF

Trial Managers:	Elizabeth Brodnicki	Tel:	020 7670 4783
	Aminata Sy	Tel:	020 7670 4737
	Denise Ward	Tel:	020 7670 4877
	Susie Slater	Tel:	020 7670 4789
Data Manager:	Selin Yurdakul	Tel:	020 7670 4874
	Kaya Widuch	Tel	0207670 4890
Statisticians:	Alex Szubert	Tel:	020 7670 4746
	David Dunn	Tel:	020 7670 4739
Clinical Project Managers	Nafisah B. Atako	Tel:	020 7670 4896
	Tom Cole (ICL)	Tel:	020 3313 6198
Project Lead:	Sheena McCormack	Tel:	020 7670 4708

Chief Investigator

Dr Katrina Pollock
NIHR Imperial Clinical Research Facility
ICTEM Building, Hammersmith Hospital Campus
Du Cane Road
London W12 0HS
UK

Tel: 020 3313 8070
Fax: N/A
Email: k.pollock@imperial.ac.uk

Scientific Lead for COVAC vacccine Programme

Professor Robin Shattock
Imperial College London

Tel: 020 7594 5206

Laboratory Operations Manager

Dr Hannah Cheeseman
Imperial College London

Tel: 020 7594 2540

Co-Investigators

Saul Faust, Principal Investigator	NIHR Southampton Clinical Research Facility, University Hospital Southampton NHS Foundation Trust
Hana Hassanin, Principal Investigator	University of Surrey Clinical Research Facility, Guildford
Catherine Cosgrove, Principal Investigator	St George's Vaccine Institute, St George's University Hospitals NHS Foundation Trust, London
Katrina Pollock, Principal Investigator	NIHR Imperial Clinical Research Facility, Hammersmith Hospital, Imperial College Healthcare NHS Trust, London
Alan Winston-O'Keefe, Principal Investigator	Imperial College Clinical Trials Centre, St Mary's Hospital, Imperial College Healthcare NHS Trust, London
Marta Boffito, Principal Investigator	Clinical Research Facility, Chelsea and Westminster Hospital NHS Foundation Trust, London
Vincenzo Libri, Principal Investigator	NIHR UCLH Clinical Research Facility, University College London Hospitals NHS Foundation Trust, London

Chair of the Trial Steering Committee

Professor Robert Lechler
King's College London

NB: throughout this document, "MRC CTU at UCL" is generally abbreviated to "CTU".

SUMMARY OF TRIAL

SUMMARY INFORMATION TYPE	SUMMARY DETAILS
**Acronym**	COVAC1
**Long Title of Trial**	A first-in-human clinical trial to assess the safety and immunogenicity of a self-amplifying ribonucleic acid (saRNA) vaccine encoding the S glycoprotein of SARS-CoV-2, the causative agent of COVID-19
**Version**	7.0
**Date**	06 Nov 2020
**EudraCT #**	2020-001646-20
**IRAS ID**	279315
**Study Design**	The study has three components:<br>➢ ***Open-label, non-randomised dose escalation*** in 15 individuals aged 18-45 years enrolled through a single centre<br>➢ ***Randomised dose evaluation*** in 105 individuals aged 18-45 years enrolled through a single centre. Participants and laboratory staff will be blind to allocation.<br>➢ ***Open-label, expanded safety evaluation*** of the highest dose (1 µg) in at least a further 200 individuals aged 18-75 years enrolled through multiple centres<br>As part of an amendment to protocol v5.0 a new cohort will be recruited to assess higher dose levels as follows:<br>➢ ***Open-label, non-randomised dose escalation*** in 12 individuals aged 18-45 years.<br>➢ ***Non-randomised dose evaluation*** in 60 individuals aged 18-45 years. Participants and laboratory staff will be blind to allocation.<br>In addition participants in the ***open-label, expanded safety evaluation*** will be able to delay their second injection until the safety data following the first vaccinations with the higher doses are available. They will be offered the option of the highest dose studied for their second injection, provided this is well tolerated.
**Setting**	Secondary care (NHS), and academic research facilities
**Type of Participants to be Studied and Justification**	Healthy adults aged 18-75 years who do not have active conditions that require investigation or a change in treatment, and whose risk of exposure to SARS-CoV-2 is similar to the general population.<br>The upper age limit will be 45 years for the dose escalation and dose evaluation as this is a first in human study, and there is greater variability of immune responses in those aged over 45 years respectively. In order to accurately assess the immune responses, individuals with no evidence of natural infection will be enrolled. The sample size calculation for the first dose evaluation part of this study is based on testing the slope co-

SUMMARY INFORMATION TYPE	SUMMARY DETAILS
	efficient in a linear regression, using the $\log_{10}$ scale for dose. The study has 97% statistical power ($2\alpha=0.05$) to detect a true difference of 0.7 ($\log_{10} IC_{50}$ scale) between adjacent doses (corresponding to a slope of 1.4), 91% power a difference of 0.6, and 79% power a difference of 0.5.<br>In order to prepare for clinical efficacy testing in the winter of 2020–2021, the expanded safety evaluation will include the older age group because of the greater benefit from an effective vaccine in this population. Forty-two participants will receive 1 µg in the dose escalation and evaluation components and a further 200 (minimum) will be recruited in the expanded safety evaluation. A sample this size has more than 90% chance of observing at least 1 serious adverse reaction (SAR) should the true frequency be 1/100.
**Sponsor**	Imperial College London
**Interventions to be Compared**	LNP-nCoVsaRNA vaccine at six dose levels
**Objectives**	➢ To compare the safety of two immunisations with LNP-nCoVsaRNA administered IM 4 weeks apart at six different dose levels in 192 participants age 18-45 years<br>➢ To evaluate the safety of two immunisations of LNP-nCoVsaRNA administered IM 4 or 14 weeks apart at various dose levels in 414 participants age 18-75 years<br>➢ To compare the immunogenicity of two immunisations with LNP-nCoVsaRNA administered IM 4 weeks apart at six different dose levels in 192 participants age 18-45 years
**Outcome Measures**	➢ Solicited local injection site reactions starting within 7 days of administration of the vaccine: pain, tenderness, erythema, swelling<br>➢ Solicited systemic reactions starting within 7 days of administration of the vaccine: pyrexia, fatigue, myalgia, headache, chills, arthralgia<br>➢ Unsolicited adverse reactions (ARs) throughout the study period (including serious ARs)<br>➢ Serious Adverse Events<br>➢ Unsolicited adverse events throughout the study period<br>➢ The titre of serum neutralising antibodies 2 weeks after the second vaccination in the SARS-CoV-2 pseudovirus-based neutralization assay<br>➢ The titre of vaccine-induced serum IgG binding antibody responses to the SARS-CoV-2 S glycoprotein 2 weeks after the first and second vaccinations
**Exploratory Objectives**	➢ To characterise the humoral and cellular immune responses to LNP-nCoVsaRNA administered at six different doses<br>➢ To characterise the profile of class and sub-class of antibody response

SUMMARY INFORMATION TYPE	SUMMARY DETAILS
	➢ To characterise infection-induced immune responses in participants with naturally acquired infection who are also exposed to the vaccine
**Exploratory Outcome Measures**	➢ Cell-mediated vaccine-induced immune responses measured by T- and B-cell ELISpot in participants in the dose escalation and evaluation cohorts<br>➢ Cell-mediated vaccine-induced immune responses measured by flow cytometry and intracellular cytokine staining in participants in the dose escalation and evaluation cohorts<br>➢ The profile of class and sub-class of antibody response<br>➢ Laboratory markers of infection and infection-induced immunity
**Randomisation**	The only randomised component is the dose evaluation in 105 participants in the first dose evaluation cohort.
**Number of Participants to be Studied**	Up to 420
**Duration**	Each participant will be followed for one year. The trial is anticipated to start in June and complete enrolment within 3-4 months.
**Funder**	MRC, via UK Research and Innovation.
**Chief Investigator**	Dr Katrina Pollock

TRIAL SCHEMA

Table 1: **Groups to be studied**

Group	Description	Dose prime (μg)	Dose boost (μg)	Age (years)	Participating centre	N
A1	First cohort dose escalation	0.1<br>0.3<br>1	0.1<br>0.3<br>1	18-45	ICRF	4<br>4<br>7
A2	Second cohort dose escalation	2.5<br>5<br>10	2.5<br>5<br>10	18-45	ICRF	4<br>4<br>4
					Total Group A	27
B1	First cohort randomised dose evaluation	0.1<br>0.3<br>1	0.1<br>0.3<br>1	18-45	ICRF	35<br>35<br>35
B2	Second cohort dose evaluation	2.5<br>5<br>10	2.5<br>5<br>10	18-45	ICRF and collaborating centres	20<br>20<br>20
					Total Group B	165
C	Expanded safety evaluation	1	1, 2.5, 5 or 10	18-75	Collaborating centres	222
					Total Group C	222
					Total	414

Figure 1: Trial Entry through the First Dose Evaluation Cohort (Groups A1 and B1) and Expanded Safety First Dose (Group C)

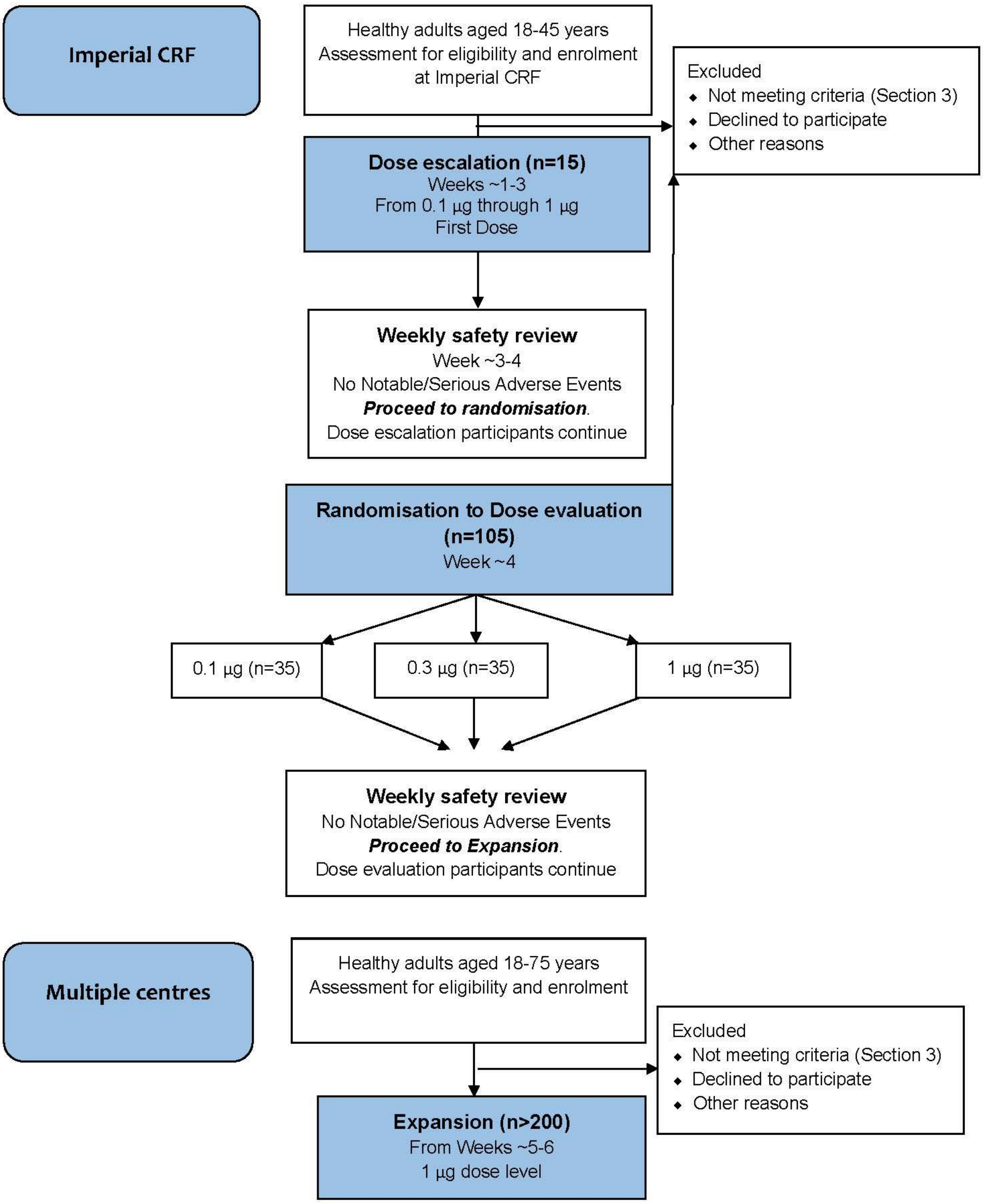

Figure 2: Trial Entry through the Second Dose Escalation (Group A2) and Evaluation (Group B2) and Second Dose in Expanded Safety group (Group C)

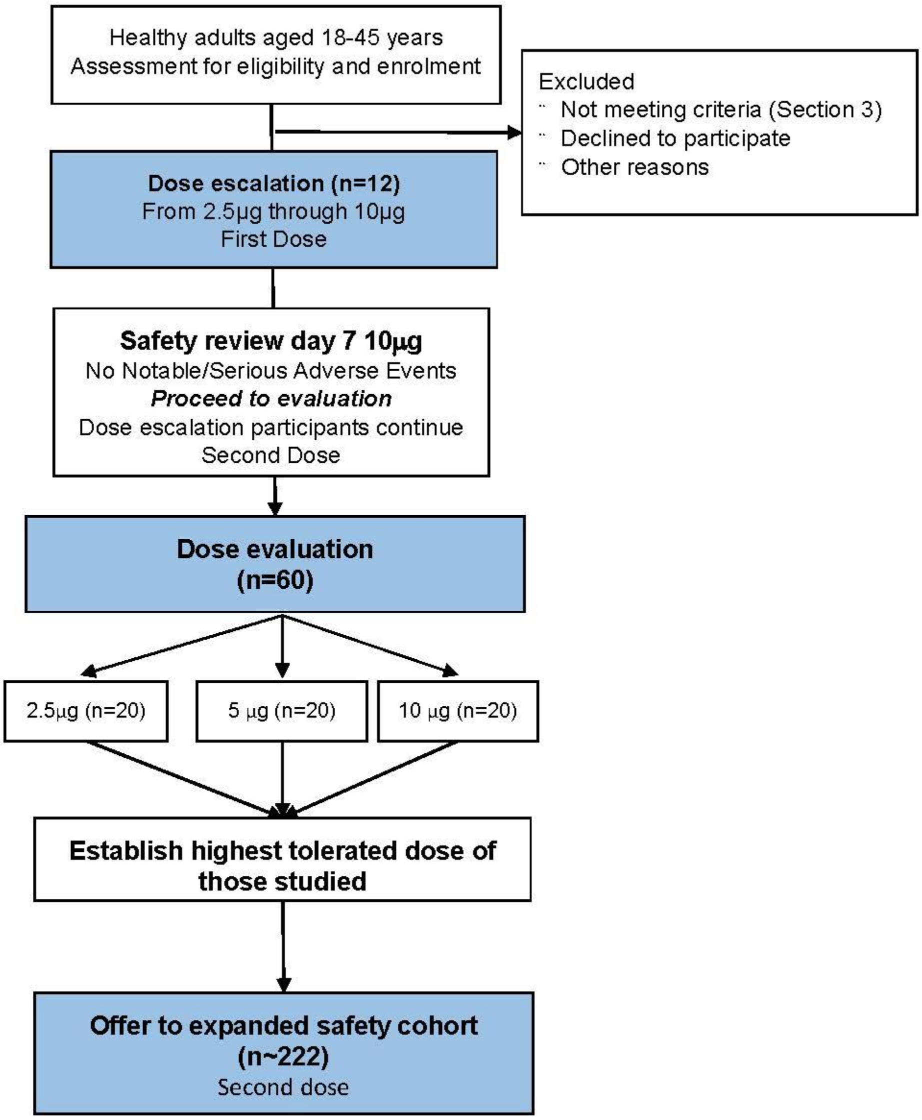

TRIAL ASSESSMENT SCHEDULES

Table 2: **Trial Assessment Schedule for dose escalation and dose evaluation, both cohorts (Groups A and B)**

**Trial Visit**	**V1**	**V2**	**V2a**	**V3**	**V4**	**V5**	**V5a**	**V6**	**V7**	**V8**	**V9**	**V10**	**V11**
Visit Type (site or telephone)	Site	Site	Phone	Site	Site	Site	Phone	Site	Site	Site	Site	Site	Site
Trial Week	–6	0	0	1	2	4	4	5	6	8	12	24	52
Trial Day	–	0	2	7	14	28	30	35	42	56	84	168	364
Windows (days)	–42 to –1	–	none	0 to +2	–2 to +2	–4 to +10	none	0 to +2	–2 to +2	–4 to +4	–7 to +7	–20 to +7	–36 to +7
Informed consent	X												
Medical history demographics	X												
Eligibility assessment	X	X											
Physical examination	X	X		X		X		X					
Weight/height (BMI)	X												
Vital signs (BP, HR, O_2 saturation and oral temperature)	X	X		X	X	X		X	X	X	X	X	X
Concomitant medication	X	X		X	X	X		X	X	X	X	X	X
Randomisation[1]		X											
Vaccination		X				X							
Issue diary card for AEs		X				X							
Review diary card for AEs			X	X			X	X					
Symptom-directed physical examination					X				X	X	X	X	X
Record COVID-19 symtoms, swab if COVID-19 suspected	X	X	X	X	X	X	X	X	X	X	X	X	X
Record adverse events	X	X	X	X	X	X	X	X	X	X	X	X	X
HIV and HCV screen	~5 mL												
SARS-CoV-2 antibodies	~5 mL												
Laboratory safety tests[2]	~10 mL	~10 mL		~10 mL	~10 mL	~10 mL		~10 mL	~10 mL	~10 mL	~10 mL	~10 mL	~10 mL
Urine dipstick	X												
Urinary pregnancy test (WOCP only)	X	X				X					X		
Blood for central serum immunogenicity assays		6 mL		6 mL	6 mL	6 mL		6 mL	6 mL	6 mL	6 mL	6 mL	6 mL
Blood for central cellular immunogenicity assays		48 mL				48 mL			48 mL		48 mL		
Blood volume (approx.)	20 mL	64 mL		16 mL	16 mL	64 mL		16 mL	64 mL	16 mL	64 mL	16 mL	16 mL

1 Only at Imperial CRF during the first dose evaluation (0.1, 0.3 and 1 µg LNP-nCoVsaRNA)

2 Haemoglobin, lymphocytes, neutrophils, platelets, creatinine, AST/ALT, ALP, total bilirubin, non-fasting glucose throughout. GGT at screening only.

Table 3: **Trial Assessment Schedule for expanded safety evaluation (Group C)**

**Trial Visit**	**V1**	**V2**	**V2a**	**V3**	**V4**	**V4a[1]**	**V4b[1]**	**V5**	**V5a**	**V6**	**V7**	**V8**	**V9**	**V10**	**V11**
Visit Type (site or telephone)	Site	Site	Phone	Site	Site	Site	Phone	Site	Phone	Site	Site	Site	Site	Site	Site
Trial Week no delay	–6	0	0	1	2	-	-	4	4	5	6	8	12	24	52
Trial Week with delay[1]	–	0	0	1	2	4[1]	8[1]	14[1]	14[1]	15[1]	16[1]	18[1]	22[1]	-[1]	52
Windows (days)	–42 to –1	–	none	0 to +2	–2 to +2	-2 to +14	-2 to +28	–4 to +10[1]	none	0 to +2	–2 to +2	–4 to +4	–7 to +7	–20 to +7	–36 to +7
Informed consent	X														
Medical history demographics	X														
Eligibility assessment	X	X													
Physical examination	X	X		X				X		X					
Weight/height (BMI)	X														
Vital signs (BP, HR, O_2 saturation and oral temperature)	X	X		X	X	X		X		X	X	X	X	X	X
Concomitant medication	X	X		X	X	X		X		X	X	X	X	X	X
Vaccination		X						X							
Issue diary card for AEs		X						X							
Review diary card for AEs			X	X					X	X					
Symptom-directed physical examination					X	X					X	X	X	X	X
Record COVID-19 symtoms, swab if COVID-19 suspected	X	X	X	X	X	X	X	X	X	X	X	X	X	X	X
Record adverse events	X	X	X	X	X	X	X	X	X	X	X	X	X	X	X
HIV and HCV screen	~5mL														
Laboratory safety tests[2]	~10 mL	~10 mL		~10 mL	~10 mL	~10 mL		~10 mL		~10 mL	~10 mL	~10 mL	~10 mL	~10 mL	~10 mL
Urine dipstick	X														
Urinary pregnancy test (WOCP only)	X	X						X					X		
Blood for central serum immunogenicity assays		6 mL		6 mL	6 mL	6 mL		6 mL		6 mL	6 mL	6 mL	6 mL	6 mL	6 mL
Blood volume (approx.)	15 mL	16 mL		16 mL	16 mL	16 mL		16 mL		16 mL	16 mL	16 mL	16 mL	16 mL	16 mL

1 If visit 5 (second vaccination) is delayed, participants will be invited to attend at week 4 (visit 4a) for an AE assessment, routine bloods and serology selecting the **unscheduled visit option on the worksheets/eDC**. Visit 4b can take place on the phone. If delayed, the window around the second vaccination can be wider -14/+28 days (weeks 12–20). **Visit 5 worksheets and eDC should only be used for the second vaccination visit as they activate the diary.** Visits 5a, 6, 7 and 8 will be 2d, 7d, 14d and 28d after the second vaccination; visit 9 at week 22. Visit 10 is not needed.

2 Haemoglobin, lymphocytes, neutrophils, platelets, creatinine, AST/ALT, ALP, total bilirubin, non-fasting glucose throughout. GGT at screening only.

LAY SUMMARY

COVAC1 is a study that is looking at the use of a new vaccine against the virus (SARS-CoV-2) which causes COVID-19. There are two parts to the study. One part is to assess the safety of this vaccine, since this will be the first time that it has been used in humans. The second part is to see how well, and for how long, the vaccine activates the immune system. It is this activation that may provide protection against developing COVID-19. But this trial is NOT looking at whether or not the vaccine is effective in terms of protection. It is just assessing whether and how well the immune system responds.

Since this is the first time the vaccine has been used in humans, the safety will be assessed initially in healthy young adults. 15 participants aged 18-45 years will be given one of three different doses (0.1, 0.3 and 1 µg) by injection into the muscle, going slowly from the lowest to the highest over a period of several weeks. There will be careful monitoring for any reactions to the vaccine.

As long as there are no safety concerns, the second part of the study can then be started. This will see how well the immune system has been activated using different dose levels of the vaccine. About 100 participants aged 18-45 years will be given one of three doses (0.1, 0.3 and 1 µg). Chance will determine which dose each individual is given. The vaccine is given by injection into the muscle of the upper arm. Two injections, four weeks apart, are needed. There are likely to be mild side-effects near to the injection site. There may also be more general side-effects such as headache, temperature and chills. Participants will be asked to record any symptoms in an online diary. In order to see how well the immune system is responding, participants will need to give blood samples several times during the first 6 weeks; then monthly for a few months; then at 6 months and 12 months. This will happen at one study centre. If this part of the study shows that the vaccines are well tolerated, a new group aged 18-45 will be recruited to study three higher doses (2.5, 5 and 10µg) at one or more centres. If any part of the study shows that a dose is unsafe or poorly tolerated, it wll be omitted from further study.

In addition, as long as there are no serious adverse events after about 6 weeks of the second part of the trial, the safety evaluation will be expanded. A further 200 people or more, from multiple sites, will be recruited. The safety of the highest vaccine dose that is known to be well tolerated (1 µg) will be assessed at the first injection. At the second injection, participants will get a chance to have the 10 µg dose provided there are no safety concerns. The age range will now also be extended to include people up to the age of 75 years. This is important, since we know older people are more at risk of serious complications from COVID-19, and so are most likely to benefit from a successful vaccine.

An independent steering committee will regularly review the information on safety and look at the immune responses to see which dose of the candidate vaccine should go forward to effectiveness testing in future trials. Before this study can start, the protocol describing the procedures and information to be provided to volunteers will have been reviewed by the national drug authority and a multi-centre research ethics committee.

CONTENTS

GENERAL INFORMATION II

SUMMARY OF TRIAL V

TRIAL SCHEMA VIII

TRIAL ASSESSMENT SCHEDULES XI

LAY SUMMARY XIV

CONTENTS XV

ABBREVIATIONS XIX

1 BACKGROUND 1
1.1 INTRODUCTION 1
1.1.1 Modification and stabilisation of the S glycoprotein immunogen 3
1.1.2 Manufacturing process 4
1.1.3 Pre-clinical Data 4
1.2 POTENTIAL RISKS AND MITIGATIONS 8
1.2.1 Established use of VEEV as the saRNA backbone 8
1.2.2 Lipid nanoparticles (LNPs) delivery system 8
1.2.3 Strong Th1 biased response to antigen 8
1.2.4 Antibody-dependent enhancement (ADE) of COVID-19 8
1.3 POTENTIAL BENEFITS 9
1.4 RATIONALE FOR CURRENT STUDY 9
1.4.1 Objectives 9
1.5 STUDY DESIGN 10
1.5.1 First dose escalation and evaluation 10
1.5.2 Second dose escalation and evaluation 11
1.5.3 Expanded safety evaluation 15
1.5.4 Rationale for choice of trial population 15

2 SELECTION OF SITES/CLINICIANS 16
2.1 SITE/INVESTIGATOR INCLUSION CRITERIA 16
2.1.1 PI's Qualifications & Agreements 16
2.1.2 Adequate Resources 16
2.1.3 Site Assessment 17
2.2 APPROVAL AND ACTIVATION 17
2.3 SITE/INVESTIGATOR EXCLUSION CRITERIA 17

3 SELECTION OF PARTICIPANTS 18
3.1 PARTICIPANT INCLUSION CRITERIA 18
3.2 PARTICIPANT EXCLUSION CRITERIA 19
3.3 NUMBER OF PARTICIPANTS 19
3.4 CO-ENROLMENT GUIDELINES 19
3.5 SCREENING PROCEDURES & PRE-RANDOMISATION INVESTIGATIONS 19

4 REGISTRATION & RANDOMISATION ... 21
4.1 RANDOMISATION PRACTICALITIES ... 21
4.2 CO-ENROLMENT GUIDELINES AND REPORTING ... 21

5 TREATMENT OF PARTICIPANTS ... 22
5.1 INTRODUCTION ... 22
5.1.1 LNP-nCoVsaRNA vaccine ... 22
5.1.2 Administration ... 23
5.1.3 Storage ... 23
5.1.4 Dispensing ... 23
5.1.5 Schedule Modifications & Interruptions ... 23
5.2 HANDLING CASES OF TRIAL MEDICATION OVERDOSE ... 24
5.3 UNBLINDING / UNMASKING ... 24
5.4 PROTOCOL TREATMENT DISCONTINUATION ... 24
5.4.1 Discontinuation Of Injections In All Participants ... 25
5.5 PARTICIPANT WITHDRAWAL ... 25
5.6 ACCOUNTABILITY & UNUSED VACCINE ... 25
5.7 COMPLIANCE & ADHERENCE ... 25
5.8 TREATMENT DATA COLLECTION ... 25
5.9 NON-TRIAL TREATMENT ... 26
5.9.1 Medications to be Used With Caution ... 26
5.9.2 Other Vaccinations ... 26
5.9.3 Management of COVID-19 symptoms ... 26

6 ASSESSMENTS & FOLLOW-UP ... 27
6.1 TRIAL ASSESSMENT SCHEDULE ... 27
6.2 PROCEDURES DURING THE SCREENING PERIOD ... 27
6.2.1 Informed Consent ... 27
6.2.2 Eligibility ... 28
6.2.3 Investigations ... 28
6.3 PROCEDURES AT ENROLMENT ... 29
6.3.1 Eligibility ... 29
6.3.2 Enrolment/Randomisation ... 29
6.3.3 Injection ... 29
6.4 PROCEDURES FOR ASSESSING SAFETY ... 29
6.4.1 COVID-19 Symptoms and SARS-CoV-2 Nucleic Acid testing ... 30
6.5 PROCEDURES FOR ASSESSING IMMUNE RESPONSES ... 30
6.5.1 Cellular immune responses ... 30
6.6 OTHER ADVERSE EVENTS ... 31
6.7 INCIDENTAL FINDINGS ... 31
6.8 EARLY STOPPING OF FOLLOW-UP ... 31
6.9 PARTICIPANT TRANSFERS ... 32
6.10 LOSS TO FOLLOW-UP ... 32
6.11 COMPLETION OF PROTOCOL FOLLOW UP ... 32

7 SAFETY REPORTING ... 33
7.1 DEFINITIONS ... 33
7.1.1 Medicinal Products ... 34
7.1.2 Adverse Events ... 34
7.1.3 Exempted Adverse Events ... 34
7.2 OTHER NOTABLE EVENTS ... 34
7.2.1 Pregnancy ... 34

7.3 INVESTIGATOR RESPONSIBILITIES ... 35
7.3.1 Investigator Assessment ... 35
7.3.2 Notification Procedure ... 36
7.4 CTU RESPONSIBILITIES ... 37

8 QUALITY ASSURANCE & CONTROL ... 38
8.1 RISK ASSESSMENT ... 38
8.1.1 Safety and Rights of Partcipants ... 38
8.1.2 Project Design and Reliability ... 39
8.1.3 Project Management and Governance ... 39
8.2 CENTRAL MONITORING AT CTU ... 39
8.3 ON-SITE AND REMOTE MONITORING ... 39
8.3.1 Direct Access to Participant Records ... 39
8.3.2 Confidentiality ... 39

9 STATISTICAL CONSIDERATIONS ... 40
9.1 METHOD OF RANDOMISATION ... 40
9.2 OUTCOME MEASURES ... 40
9.3 SAMPLE SIZE ... 40
9.4 INTERIM MONITORING & ANALYSES ... 41
9.5 ANALYSIS PLAN ... 42

10 REGULATORY & ETHICAL ISSUES ... 43
10.1 COMPLIANCE ... 43
10.1.1 Regulatory Compliance ... 43
10.1.2 Site Compliance ... 43
10.1.3 Data Collection & Retention ... 43
10.2 ETHICAL CONDUCT ... 43
10.2.1 Ethical Considerations ... 43
10.2.2 Favourable Ethical Opinion, HRA Approval, and Host Site Permission ... 45
10.3 COMPETENT AUTHORITY APPROVALS ... 45
10.4 TRIAL CLOSURE ... 46

11 INDEMNITY ... 47

12 FINANCE ... 48

13 OVERSIGHT & TRIAL COMMITTEES ... 49
13.1 TRIAL MANAGEMENT TEAM (TMT) ... 49
13.2 TRIAL MANAGEMENT GROUP (TMG) ... 49
13.3 TRIAL STEERING COMMITTEE (TSC) ... 49
13.4 INDEPENDENT DATA MONITORING COMMITTEE (I[DMC]) ... 49
13.5 PATIENT AND PUBLIC INVOLVEMENT ADVISORY GROUPS ... 49
13.6 ROLE OF STUDY SPONSOR ... 50

14 PATIENT AND PUBLIC INVOLVEMENT ... 51
14.1 POTENTIAL IMPACT OF PPI ... 51
14.2 IDENTIFYING PPI CONTRIBUTORS ... 51
14.3 REPORTING AND EVALUATING IMPACT OF PPI ... 51

15 PUBLICATION AND DISSEMINATION OF RESULTS 52

16 DATA AND/OR SAMPLE SHARING 53

17 PROTOCOL AMENDMENTS 54

18 REFERENCES 56

19 APPENDIX 1 – COVAC1 SUB-STUDY 59

19.1 SUMMARY OF SUB-STUDY 59
19.2 SUB-STUDY ASSESSMENT SCHEDULE 60
19.3 LAY SUMMARY 60
19.4 BACKGROUND 61
19.4.1 INTRODUCTION 61
19.4.2 POTENTIAL RISKS AND MITIGATIONS 61
19.4.3 Potential Benefits 61
19.4.4 Rationale for sub-study 61
19.4.5 Objectives 61
19.4.6 Outcome measures 62
19.4.7 Study Design 62
19.5 SELECTION OF SITES/CLINICIANS 62
19.6 SELECTION OF PARTICIPANTS 62
19.6.1 Participant Inclusion Criteria 62
19.6.2 Number of PaRtiCIPants 62
19.7 ASSESSMENTS & FOLLOW-UP 63
19.7.1 Trial Assessment Schedule 63
19.7.2 Informed consent 63
19.7.3 Procedures for Assessing Immune Responses 63
19.7.4 Early Stopping of Follow-up 63
19.7.5 Loss to Follow-up 64
19.7.6 Completion of sub-study follow up 64
19.8 DATA MANAGEMENT, RISK ASSESSMENT AND MONITORING 65
19.8.1 Data Management 65
19.8.2 Risk Assessment 65
19.8.3 Monitoring 65
19.9 STATISTICAL CONSIDERATIONS 67
19.9.1 Sample Size 67
19.9.2 Analysis Plan 67
19.10 REGULATORY & ETHICAL ISSUES 68
19.10.1 Compliance 68
19.10.2 Ethical Conduct 68
19.10.3 Competent Authority Approvals 68
19.10.4 Sub-study closure 68
19.11 INDEMNITY 69
19.12 FINANCE 70
19.13 OVERSIGHT & TRIAL COMMITTEES 71
19.13.1 Role of Study Sponsor 71
19.14 PATIENT AND PUBLIC INVOLVEMENT 72
19.15 PUBLICATION AND DISSEMINATION OF RESULTS 72
19.16 DATA AND/OR SAMPLE SHARING 73
19.17 REFERENCES 73

ABBREVIATIONS

ABBREVIATION	EXPANSION
A&E	Accident and Emergency
ADE	Antibody-dependent enhancement
AE	Adverse event
AIDS	Acquired Immune Deficiency Syndrome
ANA	Antinuclear antibody
AR	Adverse reaction
ART	Antiretroviral therapy
BMI	Body mass index
CF	Consent Form
CI	Chief Investigator
CI	Confidence interval
CLRN	Comprehensive Local Research Network
COM	Clinical Operations Manager
COVID-19	Coronavirus disease 19
CPM	Clinical Project Manager
CRF	Case Report Form
CRN	Clinical Research Network
CTA	Clinical Trials Authorisation
CTAAC	Clinical Trials Awards and Advisory Committee
CTIMP	Clinical trial of an investigational medicinal product

Abbreviation	Expansion
CTL	Cytotoxic T-lymphocyte
CTU	*See MRC CTU at UCL*
DCF	Data Clarification Form
DH	Department of Health
DM	Data Manager
DMC	Data Monitoring Committee
DNA	Deoxyribonucleic acid
DPA	(UK) Data Protection Act
DSUR	Developmental Safety Update Report
ECG	Electrocardiogram
eCRF	Electronic case report form
eDC	Electronic Data Capture
EFGCP	European Forum for Good Clinical Practice
ELISPOT	Enzyme-linked immunosorbent spot
EMA	European Medicines Agency
EU	European Union
EudraCT	European Union Drug Regulatory Agency Clinical Trial
FDA	(US) Food and Drug Administration
GCP	Good Clinical Practice
GP	General Practitioner

ABBREVIATION	EXPANSION
HIV	Human Immunodeficiency Virus
HRA	Health Research Authority
IB	Investigator Brochure
ICH	International Conference on Harmonisation of Technical Requirements for Registration of Pharmaceuticals for Human Use
IDMC	Independent Data Monitoring Committee
IFN	Interferon
IgG	Immunoglobulin G
IL	Interleukin
IM	Intramuscular
IMP	Investigational medicinal product
IRAS	Integrated Research Application System
ISF	Investigator Site File
ISRCTN	International Standard Randomised Controlled Trial Number
ITT	Intention-to-treat
IUD	Intrauterine device
LNP	Lipid nanoparticles
LNP-nCoVsaRNA	Lipid nanoparticle new coronavirus self-amplifying ribonucleic acid
MedDRA	Medical Dictionary for Regulatory Activities
MHRA	Medicines and Healthcare products Regulatory Agency
mL	Millilitre
MRC	Medical Research Council
MRC CTU at UCL	Medical Research Council Clinical Trials Unit at University College London (also generally abbreviated to “CTU”)
MREC	Multi-centre Research Ethics Committee
mRNA	Messenger ribonucleic acid

ABBREVIATION	EXPANSION
MVA	Modified vaccinia Ankara
NCRN	National Cancer Research Network
NHP	Non-human primate
NHS	National Health Service
NHSCR	National Health Service Central Register
NHS-IC	National Health Service Information Centre
NIHR	National Institute for Health Research
NIHR CSP	National Institute for Health Research Co-ordinated System for gaining NHS Permission
NOAEL	No Observed Adverse Effect Level
NRES	National Research Ethics Service
PEG	Polyethylene glycol
PI	Principal Investigator
PIS	Participant Information Sheet
QMAG	Quality Management Advisory Group
QP	Qualified Person
R&D	Research and Development

ABBREVIATION	EXPANSION
REC	Research Ethics Committee
RGC	Research Governance Committee
RNA	Ribonucleic acid
SAE	Serious adverse event
SAP	Statistical Analysis Plan
SAR	Serious adverse reaction
saRNA	Self-amplifying ribonucleic acid
SARS	Severe acute respiratory syndrome
SARS-CoV-2	Severe acute respiratory syndrome coronavirus 2
SAS	Safety analysis set
SD	Standard deviation
SGP	Subgenomic promoter
SIR	Suspected infected and recovered
siRNA	Small interfering RNA
SPC	Summary of Product Characteristics
SSA	Site-specific approval
SSG	Scientific Strategy Group
SSI	Site-specific information
SUSAR	Suspected unexpected serious adverse reaction
TM	Trial Manager
TMF	Trial Master File
TMG	Trial Management Group
TMT	Trial Management Team

Abbreviation	Expansion
TSC	Trial Steering Committee
UAR	Unexpected adverse reaction
UKCRN	UK Clinical Research Network (now the NIHR CRN)
UN	United Nations
VEEV	Venezuelan equine encephalitis virus
VLP	Virus-like particle
WBC	White blood cells
WHO	World Health Organization
WOCP	women of childbearing potential
µg	microgram

1 BACKGROUND

1.1 INTRODUCTION

The new pandemic coronavirus, severe acute respiratory syndrome coronavirus 2 (SARS-CoV-2) emerged in humans in China sometime between October to November 2019, and the disease coronavirus infectious disease 2019 (COVID-19) was identified in China in December 2019. By the end of March 2020, SARS-CoV-2 has infected with confirmed diagnosis 1,007,103 people worldwide, resulting in 51,703 deaths and 210,577 people recovered from hospitalised disease. This yields a nominal infection fatality rate (IFR) of ~5%. This number is likely an overestimate, due to hidden, asymptomatic or mild, and non-diagnosed cases. Although the true infection fatality rate is not known, estimates range from 10-fold lower (0.5%), based on epidemiological modelling, to 1.4% based on accurate case ascertainment in South Korea.

This pandemic is the most serious infectious disease in humans since Spanish influenza in 1918 which killed an estimated 17-50 million people. At the current attack rate, and assuming eventually 80% of the world population become infected in the first year, without significant medical interventions, SARS-CoV-2 could kill well in excess of 40 million people, eclipsing the 32 million that have died from HIV/AIDS.

COVID-19 can be a mild to moderate self-limiting disease in about 80% of infected people. These people experience symptoms of fever, myalgia, dry persistent cough and shortness of breath. This disease course is usually complete in 7-10 days, but recovery to full health may take a little longer. However, in ~20% of cases, a more aggressive and severe disease occurs, either with rapid progression from symptom onset or a rapid decline from the initial 7-10 days of moderate infection when recovery was apparently beginning. Such serious disease may require breathing support and ~20-25% of the serious cases become critically ill. The IFR for critical patients is historically ~40-50%, consistent with the overall IFR of 1-2 people per 100 diagnosed as infected.

In the UK, susceptible infected and recovered (SIR) epidemiological models are predicting reasonably accurately the epidemic curve slopes and peak of virus infection given the complete suppression strategy. Unfortunately, these same models predict that only ~10-20% of the population are likely to have been infected in the current wave of infections, meaning that a larger second wave of infection awaits the northern hemisphere in the winter of 2020/21.

The developing assessment is that the only way the world can exit from the COVID-19 pandemic is through the deployment of an effective vaccine. The problem comes with the scale needed and the time frame in which new vaccines need to be developed. A self-amplifying RNA (saRNA) vaccine provides a novel, feasible, and time-sensitive solution to begin to address the SARS-CoV-2 problem in 2020.

A number of studies have demonstrated that nucleic acid-based vaccination can protect against viral infections in non-human primate (NHP) studies [1-6], providing proof of concept that gene-based vaccination can induce protective antibodies. However, DNA vaccines have thus far failed to live up to their full potential as a standalone vaccine technology and require multiple immunizations with the use of electroporation to induce significant immune response in humans [7]. Non-replicating mRNA-based therapeutics have been widely explored in the field of oncology [8] and more recently against viral infections [9-10], but typically use high doses of RNA (100-600 µg) [10]. The requirement for high doses, and associated cost, suggest non-replicating mRNA may struggle to produce the potential hundreds of millions of doses required to rapidly respond to a pandemic. In contrast, small animal and non-human primate (NHP) experiments suggest that saRNA induces significantly enhanced responses in comparison to either DNA vaccines delivered with electroporation or mRNA. Indeed, a single

immunization with a saRNA vaccine has shown protection against Ebola virus in animal models [1]. Should a single 1 µg dose of saRNA provide protection from COVID-19, this would provide critical advantages for manufacturing where a million doses can by synthesised in a one litre reaction volume [11] with a 300-400 fold development window over mRNA vaccines. In this respect, the delivery of our COVID-19 vaccine using saRNA offers significant advantages over more conventional vaccine platforms (viral vectors, recombinant proteins and attenuated pathogens) in terms of cost, speed and reactogenicity. In contrast to viral vectors, lack of anti-vector immunity provides the opportunity for repeat immunizations with multiple RNA-encoded immunogens.

We have developed a specific codon-optimised construct that facilitates membrane expression of mature trimeric glycoproteins of SARS-CoV-2. This construct contains unique sequence modifications that facilitate surface expression of membrane bound pre-fusion native-like trimer, the major target for neutralising antibodies [12]. Our approach is based on strong scientific evidence that gene-based approaches can protect against viral infection in animal models including NHP [1-6] and that protection is mediated by induction of neutralising antibodies providing a serological marker of efficacy [1]. We have identified the synthetic saRNA platform as the most cost-effective approach to develop our COVID-19 vaccine [11-15]. Our chosen vector is based on a non-infectious Venezuelan equine encephalitis virus (VEEV) replicon backbone encoding non-structural proteins required for self-amplification, where the SARS-CoV-2 S glycoprotein has been inserted in place of structural genes downstream of the subgenomic promoter (SGP; Figure 2).

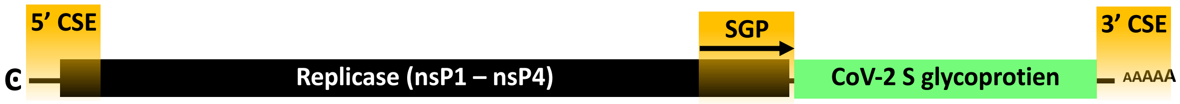

Figure 2. Structural configuration of the COVID-19 saRNA vaccine

The saRNA is strictly confined to the cytosol, does not require a DNA intermediary or penetration into the nucleus, and can generate high expression of a gene product with a relatively low initial dose [14-16]. Formulated saRNA is taken up into the cytoplasm of target cells. This leads to intracellular amplification of the saRNA by the encoded polymerase machinery and very high expression levels of the gene of interest. This process in turn induces strong immune-stimulatory potency against the selected immunogen due to its intrinsic adjuvant activity [14-17]. However, this does not generate viral particles and there is no onward transmission of the saRNA to other cells. Use of the VEEV backbone builds on recent promising data showing that a VEEV replicon could protect against other viral targets in animal challenge studies [1]. The known protective utility of gene-based glycoprotein vaccines provides a strong scientific rationale for the approach [1-6] (Figure 3).

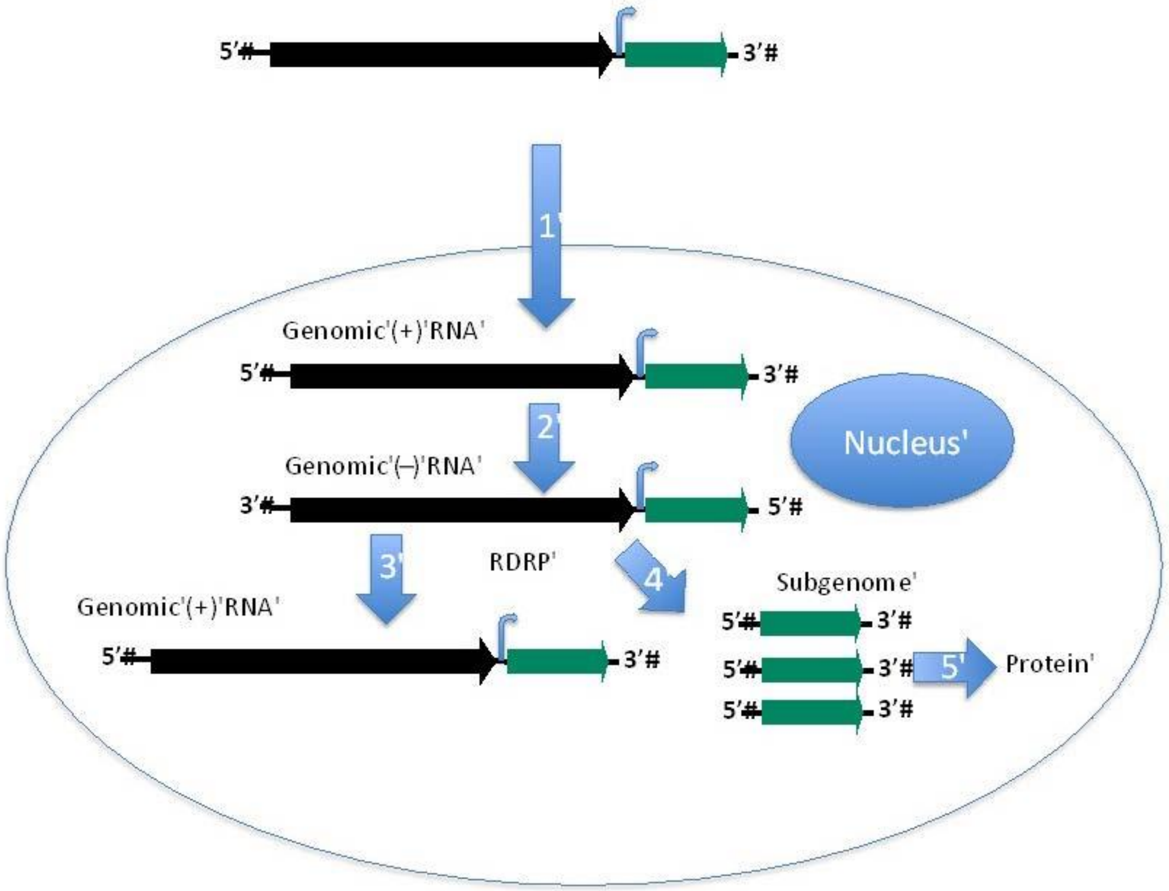

Figure 3. On delivery into cells (1), the polymerase machinery of the VEEV backbone is preferentially expressed. This in turns leads to amplification of the RNA through production of negative (2) and positive copies of the saRNA vector (3). Within a few hours, sequential cleavage of the polymerase machinery leads to a shift to preferential transcription of the subgenomic sequence encoding the S glycoprotein of SARS-CoV-2 (4). This is then translated to express high level of the S glycoprotein antigen (5).

1.1.1 MODIFICATION AND STABILISATION OF THE S GLYCOPROTEIN IMMUNOGEN

We recognise that the optimum immune response elicited by a vaccine would be one triggered by exposure to an antigen which resembles the coronavirus before it attaches to, and fuses with, a host cell, rather than after attachment and fusion with a host cell. However, the trimeric pre-fusion glycoprotein is 'meta-unstable' and can undergo conformation change spontaneously even in the absence of receptor binding. Therefore, we have made two amino acid mutations to the wild-type sequence SARS-CoV-2 surface glycoprotein (K968P and V969P). These mutations have been identified as being required for the stabilisation of the glycoprotein trimer in its native-like pre-fusion conformation [12]. Hence, our saRNA vaccine comprises a nucleic acid sequence downstream of the subgenomic promotor encoding modified codon optimised SARS-CoV-2 surface glycoprotein stabilised in a pre-fusion conformation. In this respect, stabilisation of viral glycoprotein in a pre-fusion native trimeric form optimises the induction of neutralising responses, while reducing off target responses to post-fusion intermediates and dissociated subunits. Such an approach has proven transformative in the development of respiratory syncytial virus (RSV) immunogens able to elicit high titre neutralising antibody responses [18]. It is anticipated that this will also be the case for the S glycoprotein of SARS-CoV-2. The stabilisation mutations also render the encoded S glycoprotein incapable of facilitating fusion, providing an additional safety parameter. In summary, the saRNA vector encodes a full length,

pre-fusion, stabilised, non-functional version of the SARS-CoV-2 S glycoprotein and no other structural proteins.

1.1.2 MANUFACTURING PROCESS

The manufacturing process for the generation of our saRNA drug substance consists of three main steps [11]: (1) *in vitro* saRNA transcription from the linear DNA template using bacteriophage T7 RNA polymerase followed by hydrolysis of the DNA template; (2) purification of the saRNA; and (3) the incorporation of saRNA into lipid nanoparticles (LNPs). The saRNA is encapsulated within LNPs with a unique lipid composition, using a self-assembly in-line process in which >90% of saRNA in aqueous solution is encapsulated, generating an ionizable cationic LNP of 80-100 nm in diameter. This provides protection of the nucleic acid payload against nucleases while facilitating cellular uptake and endosomal escape.

1.1.3 PRE-CLINICAL DATA

Immunogenicity has been evaluated in small murine studies and macaques, and GLP toxicology conducted in rats.

Results in mice indicate that a single immunization of 0.01 to 10 µg gives rise to high titre binding antibodies that are able to neutralize pseudotype virus expression of the functional S glycoprotein (**Figure 4**). Responses are increased following a second injection and have a strong Th1 bias based on IgG1:IgG2a ratio of elicited responses.

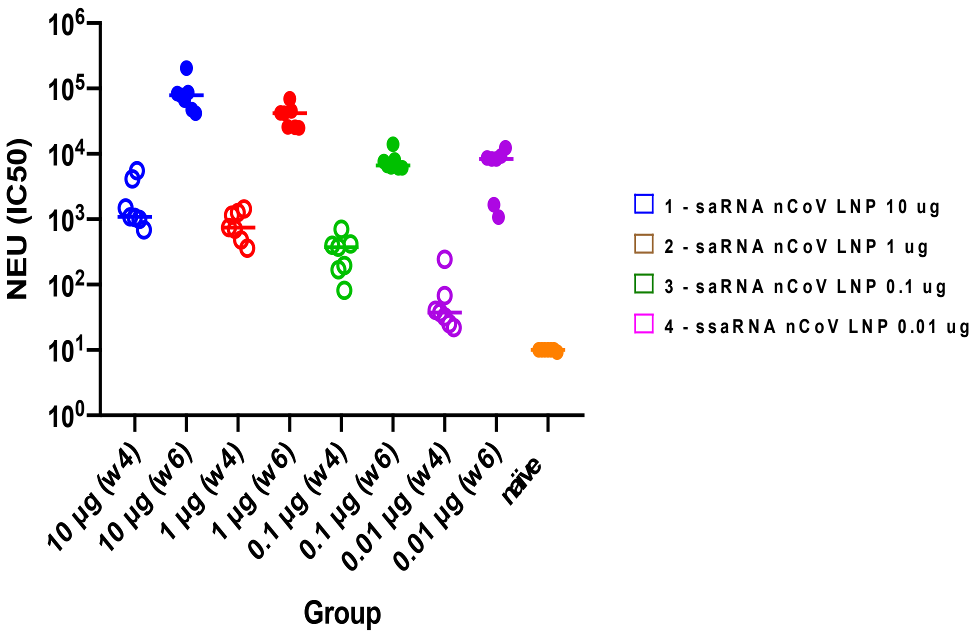

Figure 4. SARS-CoV-2 pseudotyped virus neutralization of sera from BALB/c mice vaccinated with doses of LNP-formulated saRNA ranging from 0.01-10 µg at Weeks 0 and 4.

Although increased responses were seen with a 10 µg dose in mice this was associated with pilo-erection and reduced motility for 24 hours the day after dosing.

A Good Laboratory Practice compliant preclinical toxicology study was performed in rats (see additional details in the Investigator's Brochure). The objective of the study was to determine the local and/or systemic toxic effects of the LNP-nCoVsaRNA vaccine, following three intramuscular administrations to the rat at Weeks 0, 1 and 2. Animals were immunised with 10 µg (equivalent to the highest proposed clinical dose, in absolute terms) or 1 µg of LNP-nCoVsaRNA vaccine.

Reversible, LNP-nCoVsaRNA-related clinical observations included inguinal swelling in the hind limb, following each administration, for both sexes administered 10 µg, and mildly decreased general activity on Day 2 of the dosing phase for males administered 10 µg. Elevated body temperatures, not exceeding 40 °C, were noted in both sexes following each administration at 10 µg.

Minimal, reversible body weight loss was recorded during the dosing phase for both sexes administered 10 µg, which correlated with lower food consumption.

Clinical pathology changes on Day 28 of the dosing phase were limited to animals administered 10 µg, compared with controls, and consisted of minimal decreases in hemoglobin levels, which correlated with a compensatory increase in reticulocyte values.

The GLP Study Director concluded that IM injection of LNP-nCoVsaRNA at 1 and 10 µg/dose resulted in increased incidence of localized inflammatory infiltrates at the dose sites and swelling of the inguinal lymph nodes. Both findings clearly demonstrated reversibility and were therefore not considered adverse, and the no observed adverse effect level (NOAEL) was deemed to be 10 µg/dose. The proposed starting human dose, 0.1 µg, is 100 times lower than the NOAEL in the rat study, and the highest proposed dose is equivalent, in absolute terms. On a body weight basis, taking 70 kg as the average human's body weight and 0.3 kg as the rat's, the highest proposed dose is about 230 times lower than the NOAEL.

The raised body temperature, inguinal swelling associated with the site of injection, and mildly decreased general activity in the male rats are typical of type I interferon responses [46] commonly associated with saRNA vaccination [47]. There was no advantage in terms of binding antibody responses between 1 and 10 µg of saRNA in rats (**Figure 5**).

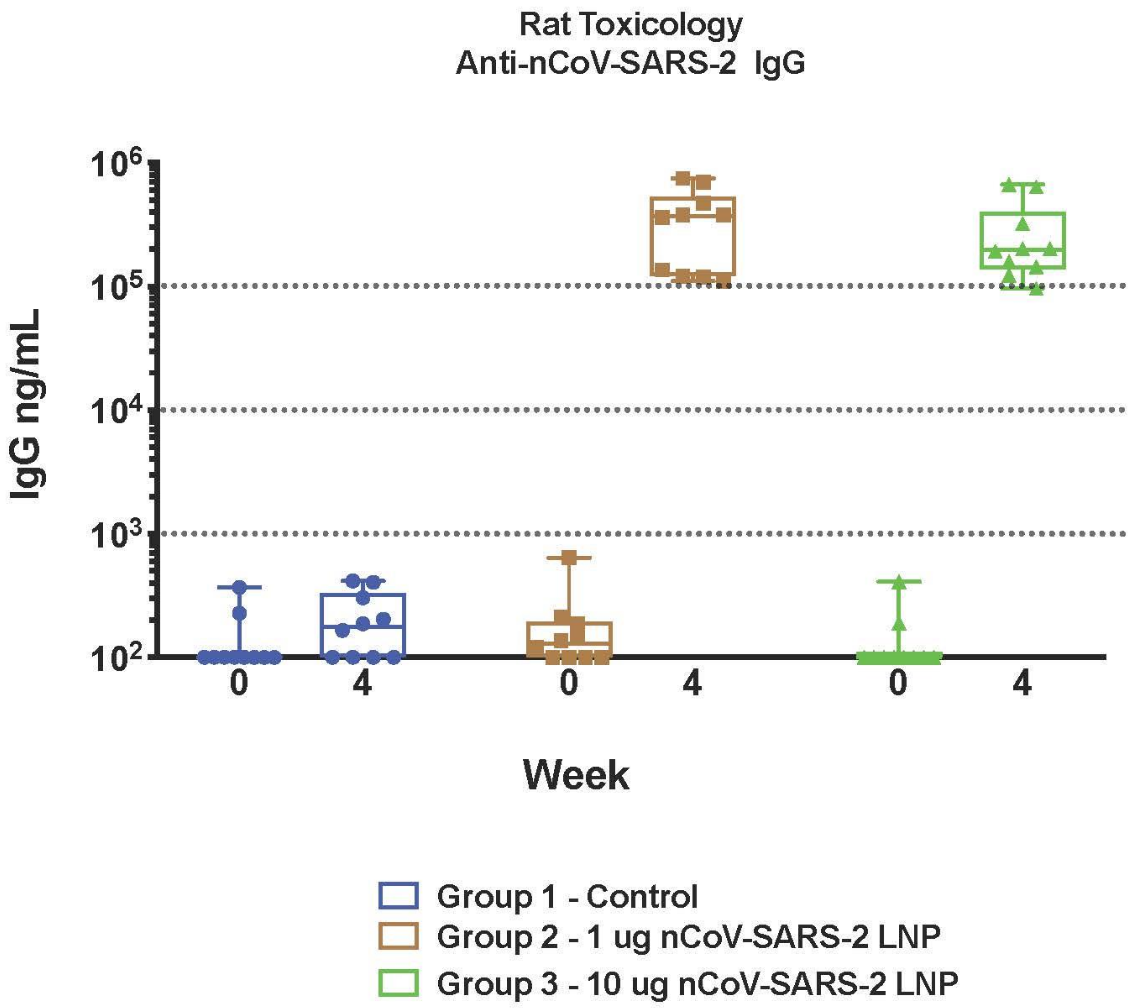

Figure 5. Binding antibodies against SARS-CoV-2 S glycoprotein of sera from rats immunized with 1 and 10 µg doses of LNP-formulated saRNA at Weeks 0, 1 and 2. Sera were taken at baseline (Week 0) and at Week 4, two weeks post last vaccination.

Although low level induction of type I interferon release that has adjuvant properties, when induced above a critical threshold such responses are counter-productive, limiting antigen expression [47]. Indeed results in cynomolgous macaques shows that while a 1 µg dose induces antibody responses in 5/6 animals two weeks after a single immunization, a 10 µg dose of saRNA induces responses in only 2/6 animals (**Figure 6**).

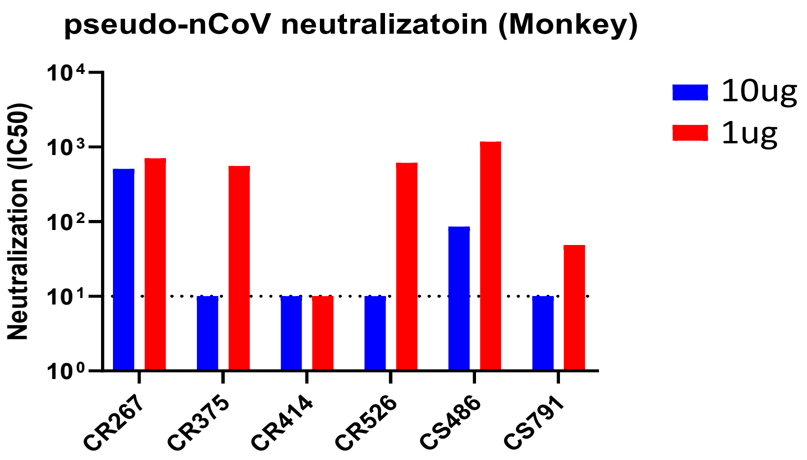

Figure 6. SARS-CoV-2 pseudotyped virus neutralization of sera from cynomologus macaques two weeks post vaccination with 1 and 10 µg doses of LNP-formulated saRNA

Cumulatively these data suggest that 0.1–10 µg is likely to be the dose range within which immune responses peak and tolerance is acceptable in humans.

In the mouse model there is only a modest difference in the level of neutralising antibodies induced by the 1 µg dose when compared with 0.1 µg. We wish to evaluate the immunogenicity of 0.1, 0.3, 1, 2.5, 5 and 10 µg dose levels, because the lowest of these dose levels would equate a 100-fold increase in manufacturing productivity over the highest. Selecting 0.1 µg as the first-in-human starting dose also provides a conservative approach in terms of safety, being 100 times lower than the NOAEL in the rat toxicology study. We have chosen to study the dose range of 0.1–10 µg because although no advantage was observed in higher order animals between 1 and 10 µg, and in fact the response appeared to be attenuated at the higher dose level (Figure 6), we wish to establish the dose response over this range in humans.

Taken together our dose selection approach is conservative while covering the range in which immunogenicity is likely to be highest in humans. If each dose level proves to be equally safe and immunogenic then we would likely take the lowest dose level forward for efficacy testing, given the advantage this would provide for manufacturing at scale.

In the mouse model, a booster immunisation given 4 weeks after the first resulted in significantly higher titres of binding and neutralising IgG antibodies (Figure 4). Four weeks is the minimal interval to see maximal benefit from the boosting immunisation. An interval of 3 weeks was less potent, and there was no significant advantage of a longer interval (6 weeks). We wish to achieve protective immunity in the shortest possible time, and therefore have selected for the clinical trial the shortest interval that does not negatively impact on the benefit of the booster immunisation.

If any of the lower proposed doses do not induce an adequate immune response, there is no known reason why participants who received those doses could not be offered a further booster immunisation with LNP-nCoVsaRNA at a dose that has been shown to be safe and effective. Also there is no known reason why participants in COVAC1 could not be immunised with other SARS-CoV-2 vaccines, including vectored or adjuvanted vaccines, should they be shown to be safe and effective.

1.2 POTENTIAL RISKS AND MITIGATIONS

1.2.1 ESTABLISHED USE OF VEEV AS THE SARNA BACKBONE

Our use of VEEV as the saRNA backbone builds on a long history in the use of VEEV for vaccination. A live-attenuated VEEV (TC-83) has been used to protect at-risk laboratory personnel humans since the 1960s with no adverse effects [19]. As a vector, alphavirus-based recombinant RNA replicons were initially packaged into viral like particles (VLPs). A number of previous clinical trials have demonstrated the safety of alphavirus-based recombinant RNA replicon viral like particles (VLPs) in humans [20-22], and NCT00439803, NCT00063778, NCT00440362, NCT01890213. More recently, with the development of formulations able to facilitate RNA uptake into cells, naked VEEV RNA has been used on its own. This still retains the same capacity for self-amplification within cells, but does not require any recombinant protein elements. This builds on the wider experience of RNA-based vaccines and pharmaceuticals that have been shown to be safe in non-clinical and clinical tests. The saRNA vaccine manufacturing process is devoid of any virus particle forming genes.

1.2.2 LIPID NANOPARTICLES (LNPS) DELIVERY SYSTEM

To ensure efficient uptake of the saRNA, it will be administered intramuscularly formulated in lipid nanoparticles (LNPs). These particles are made from a mixture of ionizable cationic lipid, phosphatidylcholine, cholesterol, and polyethylene glycol (PEG)-lipid. The saRNA is encapsulated within the LNPs protecting the RNA from degradation and delivering the payload to the cytoplasm of cells following endocytosis of the LNP. This approach builds on the successful use of LNPs for siRNA, exemplified by the granting of a license for Onpattro (patisiran) delivered in LNPs [23]. This approach has been recently applied to mRNA vaccines [10] and is being utilised for an mRNA vaccine against COVID-19 developed by Moderna [24].

1.2.3 STRONG TH1 BIASED RESPONSE TO ANTIGEN

Certain animal models raised early concerns over the potential use of alum adjuvanted, formalin inactivated SARS-CoV-1 virions in animal models [24-48]. Vaccinated animals subsequently challenged with SARS-CoV-1 exhibited a Th2 immunopathologic lung reaction reminiscent of that described for RSV [29, 30]. However, studies using a VEEV replicon particle vaccine indicated that the nucleocapsid protein of SARS-CoV-1 is the antigen to which the immunopathologic reaction is directed [31]. Critically no pathology was shown with the same vector encoding the S glycoprotein of SARS-CoV-1. Our vaccine encodes the pre-fusion stabilised version of the SARS-CoV-2 S glycoprotein and drives a strong Th1 biased response.

1.2.4 ANTIBODY-DEPENDENT ENHANCEMENT (ADE) OF COVID-19

Theoretical concerns have also been raised about the potential for antibody-dependent enhancement (ADE) by vaccine-induced antibodies against SARS-CoV-2. ADE occurs when virus-specific antibodies facilitate virus entry into host immune cells (monocytes, macrophages, granulocytes) that express Fc receptors. It is hypothesised that enhanced virus replication within these target cells could exacerbate disease [32]. Sub-neutralising (or non-neutralising) antibodies have been implicated in ADE, which has been observed for a variety of viruses, most notably flaviviruses (e.g. dengue virus), but also RSV, HIV, filoviruses and an animal coronavirus [32, 33]. The phenomenon of ADE has been mostly characterised *in vitro* [34-40] and somewhat in experimental animal models [41-43].

It is now hypothesized that antibodies raised against the post-fusion conformation of F protein of RSV can result in deleterious outcome, since they are non-neutralizing. This concern has been overcome for RSV through the use of mutations that stabilise the RSV glycoprotein in a pre-fusion conformation, and avoid the risk of ADE by directing a stronger neutralizing response [44]. We have adopted the same strategy by using a pre-fusion stabilised version of the SARS-CoV-2 S glycoprotein [12].

Thus, the risk of vaccine-induced enhancement of disease by our SARS-CoV-2 saRNA vaccine is minimised by the strong induction of functional antibody responses relative to total binding antibody, and the induction of a Th1-biased response.

It is highly unlikely that this trial will generate evidence to address this theoretical problem, due to the size and likely low incidence of ADE, if it exists (based on dengue for example). However, participants will be followed up for development of COVID-19 symptoms for one year and consent will be sought to capture long-term morbidity and mortality data through NHS Digital, in a similar way to dengue vaccine trials focusing on hospitalisations and death as signs of severe disease [45].

1.3 POTENTIAL BENEFITS

A theoretical benefit of participating in this clinical safety trial is that the vaccine may provide protection against SARS-CoV-2 infection and associated COVID-19 disease. Despite promising pre-clinical data, at this stage equipoise remains as to relative risks and benefits.

On the basis of the background risks and mitigations described above, it is considered safe and appropriate to enter a clinical early phase immunogenicity and safety trial with COVID-19 saRNA vaccine.

1.4 RATIONALE FOR CURRENT STUDY

This SARS-CoV-2 pandemic has infected over 4 million people and without significant medical interventions, could kill well in excess of 40 million people. As a novel zoonotic virus no herd immunity is present and the only interventions available to date are social distancing to reduce pressure on intensive care beds and health systems, but this is not sustainable for economic reasons. Clinical trials of antivirals and other drug therapies are ongoing but the intervention most likely to mitigate the long-term medical, social and economic impact of the pandemic remains population-wide immunisation, and there is an urgent need to find an effective candidate.

The pre-clinical data described above suggest that LNP-nCoVsaRNA will elicit neutralising antibodies in a higher proportion of individuals than natural infection (<50%), and that 0.1 µg may be as good as 10 µg. Should the lower dose prove effective 100 times as many people could be vaccinated for the same volume of product. The schedule is two immunisations given four weeks apart to maximise neutralising antibodies by eliciting an anamnestic T cell response. However, we will allow the second injection to be delayed in the expanded safety cohort so that participants can have a higher dose more likely to induce strong responses.

1.4.1 OBJECTIVES

The overall aims of the trial are to determine the dose to take forward to efficacy testing, and to build a sufficiently large safety database to enrol to an efficacy trial in winter 2020–2021 (target 200).

The stated objectives are:

- To compare the safety of two immunisations with LNP-nCoVsaRNA administered IM 4 weeks apart at six different dose levels in 192 participants age 18-45 years
- To evaluate the safety of two immunisations of LNP-nCoVsaRNA administered IM 4–18 weeks apart at various dose levels in 414 participants age 18-75 years
- To compare the immunogenicity of two immunisations with LNP-nCoVsaRNA administered IM 4 weeks apart at six different dose levels in 192 participants age 18-45 years

As this is a first-in-human trial, accrual will be limited to healthy young adults for the dose escalation and evaluation, expanding to older age groups when 42 individuals have received the first injection of 1 µg and there are no safety concerns in the 7 day (minimum) reactogenicity data. The dose escalation

and evaluation cohorts will be limited to individuals who are SARS-CoV-2 antibody negative at screening, to facilitate the analysis of immune responses to the vaccine. Although male gender is a risk factor for COVID-19, herd immunity will require immune protection in the whole population and therefore the protocol aims to recruit equal numbers of males and females.

Safety data will be assessed as an event rate and confidence interval with clear thresholds for pausing vaccinations in an individual and the trial (see Section 5). Provided the threshold for pausing vaccines in the trial is not crossed, the dose to take forward to efficacy testing will be determined by the quantity of binding antibodies at Weeks 2 and 6, and neutralising antibodies at Week 6, in relation to the levels seen in patients who have recovered from COVID-19. As placebo does not inform the analysis for either of these endpoints, there will be no allocation to placebo.

1.5 STUDY DESIGN

This is the first-in-human trial of LNP-nCoVsaRNA vaccine. The first dose escalation and evaluation will be conducted in 18-45 year olds in a single centre supervised by the Chief Investigator and two senior clinicians experienced in first-in-human studies, starting with a sentinel cohort of 15 individuals (**Figure 7**) before randomising 105 participants to one of three doses, as described in Section 1.5.1.

Dependent on the safety and immunogenicity data accrued from the first dose escalation cohort of 15, the range of doses studied in a second escalation/evaluation may be extended to include 2.5, 5 and 10 µg dose levels – shown in Figure 8 and described in Section 1.5.2.

The second dose escalation will be conducted in 18-45 year olds in the same centre that conducted the first dose escalation, supervised in the same way. The second dose evaluation will be conducted in the same centre, and also at other centres.

1.5.1 FIRST DOSE ESCALATION AND EVALUATION

The dose escalation cohort will proceed through 0.1, 0.3 and 1.0 µg as follows over the course of 2 weeks (minimum):

- The first participant will receive 0.1 µg and be invited to enter information on local and systemic reactions, into an online diary, that evening and daily thereafter for 6 days
- At 48 hours post-vaccination the team will call the first participant and go through their diary. If the reactions are Grade 1-2, or transient Grade 3 that resolve within 24 hours, three further participants will receive 0.1 µg
- At 48 hours post-vaccination the team will call participants 2, 3 and 4 and go through their diaries. Provided there are none of the safety concerns outlined above, a fifth participant will proceed to receive the next dose (0.3 µg)
- The steps described above will be repeated in order to escalate to the highest dose (1.0 µg): the sentinel at 0.3 µg will be followed by three more participants at the same dose, provided there are none of the safety concerns outlined above at 48 hours post-vaccination of the sentinel; and a sentinel at 1.0 µg will follow if there are none of the safety concerns outlined above at 48 hours post-vaccination of the participants at 0.3 µg
- Provided there are none of the safety concerns outlined above at 48 hours post-vaccination of the sentinel at 1.0 µg, a further three participants will receive 1.0 µg and, following the same safety check at 48 hours post-vaccination, a further three will receive 1.0 µg
- When all seven participants to receive 1.0 µg have attended their follow-up visits at Day 7, the safety data accrued to date will be reviewed and, if deemed acceptable using the same criteria described above, the randomised dose evaluation cohort of 105 may start to be enrolled, randomised 1:1:1 to 0.1, 0.3 or 1.0 µg

The steps above describe the fastest dose escalation plan. However, if there are persistent Grade 3 reactions observed in any of the sentinels, the team will invite the affected participant to attend the study site in order to evaluate these and will proceed to vaccinate a second sentinel at the same dose. The dose will not be escalated until 4 participants in total have provided safety data to at least 48 hours post-vaccination. If 2 or more of the participants at any dose level in the dose escalation part of the trial have persistent Grade 3 (or worse) reactions, or if 1 or more has a serious adverse reaction, the dose will not be escalated or expanded, and the trial stopping rules described below will be triggered.

The dose escalation plan described above will be repeated when the participants return for their second vaccinations at Week 4.

1.5.2 Second dose escalation and evaluation

Following a review of the cumulative safety data and preliminary immune responses in the first dose escalation cohort on 18th August, the Trial Steering Committee recommended that the trial team evaluate higher doses up to 10 μg. As of 9th August, 120 participants age 18-45 were enrolled to the 3 dose groups, 15 of whom had received their second vaccination. Reactions reported in the first 7 days were absent or mild with the most common local mild reaction being tenderness/discomfort (43%) followed by pain (15%). These appear to increase with dose. The most common mild systemic reactions are fatigue (25%) and headache (23%) with no clear relationship to dose. Moderate reactions have been infrequent: tenderness/discomfort and pain reported by 2% and 1% respectively; fatigue by 3% and headache by 3%. Preliminary immune responses suggested a dose response with earlier responses seen at higher doses, but no more than half the participants in the 0.1 and 0.3 μg groups had seroconverted. Insufficient data were available on the 1 μg group but the overall consensus was that the doses explored to date were too low.

The second dose escalation cohort will proceed through 2.5, 5 and 10 μg as follows over the course of 11 days (minimum):

1. The first participant will receive 2.5 μg and be invited to enter information on local and systemic reactions, into an online diary, that evening and daily thereafter for 6 days.
2. At 48 (–/+5) hours post-vaccination the team will call the first participant and go through his/her diary. If the reactions are Grade 1-2, or transient Grade 3 that resolve within 24 hours, three further participants may receive 2.5 μg
3. At 48 (–/+5) hours post-vaccination the team will call the next three participants and go through their diaries. If the reactions are Grade 1-2, or transient Grade 3 that resolve within 24 hours, a sentinel participant may receive 5 μg.
4. At 48 (–/+5) hours post-vaccination the team will call the sentinel participant at 5ug vaccinated in Step 3 and go through his/her diary. If the reactions are Grade 1-2, or transient Grade 3 that resolve within 24 hours, three further participants may receive 5 μg.
5. At 48 (–/+5) hours post-vaccination the team will call the three participants vaccinated in Step 4 and go through their diaries. If the reactions are Grade 1-2, or transient Grade 3 that resolve within 24 hours, a sentinel participant may receive 10 μg.
6. At 48 (–/+5) hours post-vaccination the team will call the sentinel participant vaccinated in Step 5 and go through his/her diary. If the reactions are Grade 1-2, or transient Grade 3 that resolve within 24 hours, three further participants may receive 10 μg.
7. At 48 (–/+5) hours post-vaccination the team will call the three participants vaccinated in Step 6 and go through their diaries. When all four participants to receive 10 μg have attended their follow-up visits at Day 7, the safety data accrued to date will be reviewed and, if deemed

acceptable using the same criteria described above, the second dose evaluation cohort of 60 participants may start to be enrolled to receive 2.5, 5 or 10 µg

The steps above describe the fastest dose escalation plan from 2.5–10 µg. However, if there are persistent Grade 3 reactions observed in any of the sentinels, the team will invite the affected participant to attend the study site in order to evaluate these and will proceed to vaccinate a second sentinel at the same dose. The dose will not be escalated until at least 1 participant has provided acceptable safety data to at least 48 (–/+5) hours post-vaccination. If 2 or more of the participants at any dose level in the dose escalation part of the trial have persistent Grade 3 (or worse) reactions, or if 1 or more has a serious adverse reaction, the dose will not be escalated or expanded, and the trial stopping rules described below will be triggered.

Figure 7. Fastest possible first dose escalation plan[1], provided there are no persistent Grade 3 (severe), or serious, reactions

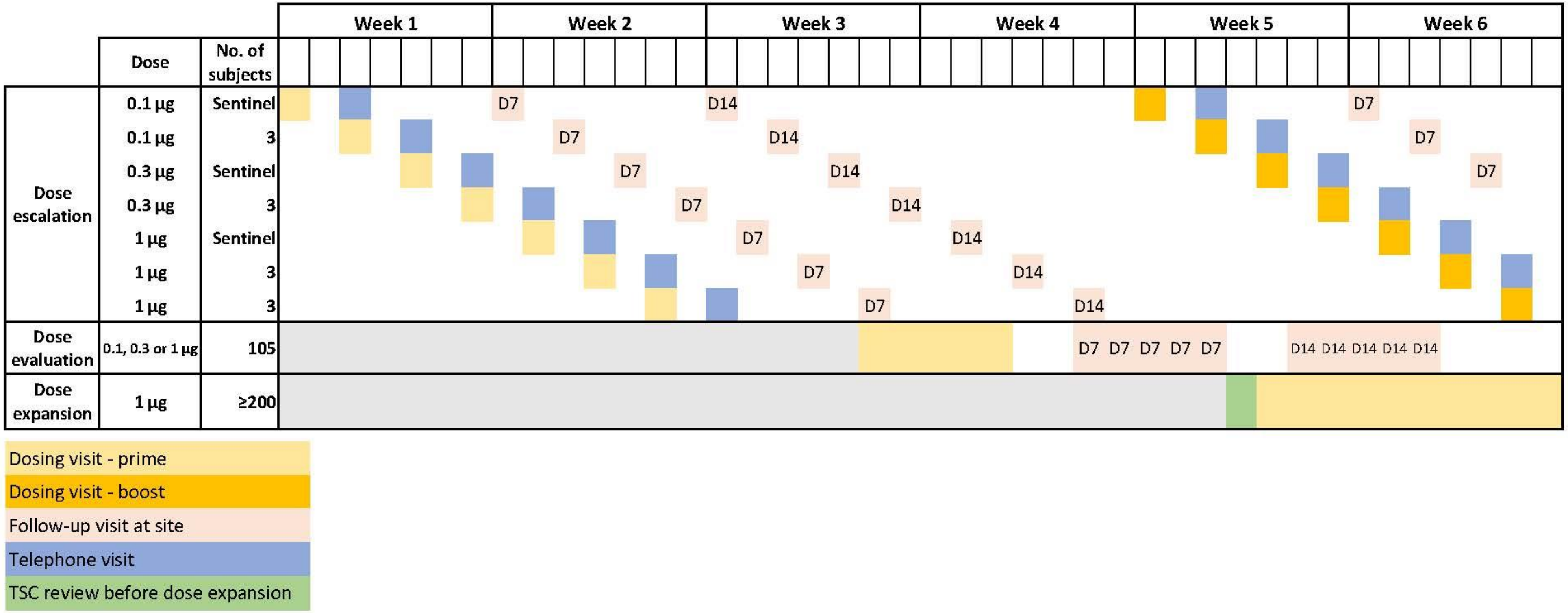

1 This is the fastest possible dose escalation plan; it is not a fixed schedule. Reasons for a slower dose escalation include persistent Grade 3 or serious reactions, availability of participants and staff, and other operational factors.

Figure 8. Fastest possible second dose escalation plan[1], provided there are no persistent Grade 3 (severe), or serious, reactions

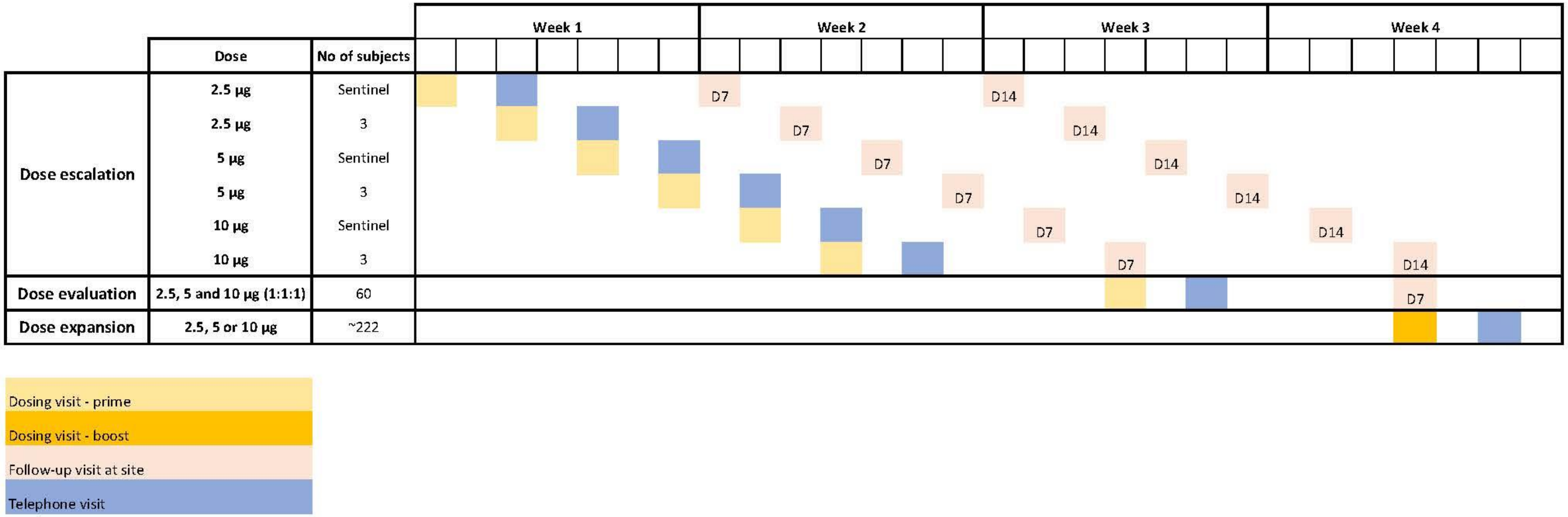

1 This is the fastest possible dose escalation plan; it is not a fixed schedule. Reasons for a slower dose escalation include persistent Grade 3 or serious reactions, availability of participants and staff, and other operational factors.

1.5.3 EXPANDED SAFETY EVALUATION

Provided there are nothing worse than transient Grade 3 reactions in the safety data collected to Day 7 (minimum) from the first 42 participants to receive one vaccination at 1 µg, further centres will be able to start injections, accumulating data in at least 200 individuals for the expanded safety evaluation of the 1 µg dose at Week 0. This will inform the safety of lower doses and is the most efficient way to proceed prior to review of immune responses.

The restriction on upper age of 45 years will be removed from the database and participants up to age 75 years will be eligible for enrolment in the expanded safety evaluation in order to evaluate immune responses across a broader age range.

At any point during the trial, persistent Grade 3 reactions or any serious adverse reactions will be immediately reported to the Trial Steering Committee (TSC). If there are two participants with persistent Grade 3 reactions, or one with a serious adverse reaction, in the first 20, vaccinations will be paused in all participants and the TSC will be asked to review the accumulated safety data and make a recommendation. If vaccinations are paused in all participants, a substantial amendment will be submitted to the competent authorities. Likewise, approval from the competent authorities is needed before the study can be resumed.

Although the original intention was for participants in the expanded safety evaluation to receive a 1 µg dose at both the first and second vaccinations, the TSC recommended that a higher, and we hope more immunogenic, dose be given at the second vaccination. We will proceed with 10 µg, provided this is sufficiently well tolerated in the second dose escalation/evaluation. The TSC agreed that we should assess the Day 7 (minimum) safety data from 20 (minimum) participants at each dose level in the second dose escalation/evaluation cohort, before asking the TSC to recommend which dose to use for the second vaccination of participants in the expanded safety evaluation. We believe this decison will be made in time for vaccinations at Week 14 of the participants' schedule. Alternatively, participants can elect to receive the 1 µg dose at Week 4, as originally planned.

1.5.4 RATIONALE FOR CHOICE OF TRIAL POPULATION

First-in-human dosing for vaccine candidates is typically in healthy young adult populations as these individuals generate the most robust responses. However, it will be important to demonstrate immunogenicity in those most at risk of severe disease. As this risk increases with age, the upper age limit for the expanded safety evaluation is 75 years, recognising that government guidance on strict social distancing of the over 70 may make it difficult for participants over 70 years old to attend trial sites. All participants will provide samples for immunogenicity assessment, including measurement of titres of serum binding and neutralising antibodies, induction of high levels of which is believed to be essential for an effective vaccine. Thus we will be able to confirm immunogenicity of the vaccine in older participants.

Both male and female participants will be included and trial sites will attempt to keep an equal proportion, although the priority will be to ensure timely accrual to the trial.

In order to study the safety and immunogenicity of LNP-nCoVsaRNA vaccine in individuals with a history of SARS-CoV-2 infection, one investigator site (at St Mary's Hospital, London) will preferentially enrol participants known to have infection-induced SARS-CoV-2 antibodies prior to enrolment. The site's enrolment target is 30 participants.

2 SELECTION OF SITES/CLINICIANS

Sites and investigators were selected based on the project proposal and grant application.

2.1 SITE/INVESTIGATOR INCLUSION CRITERIA

To participate in the COVAC1 trial, investigators and clinical trial sites must fulfil a set of basic criteria that have been agreed by the COVAC1 Trial Management Group (TMG) and are defined below.

Each site will be issued with the appropriate COVAC1 master file documentation with the regulatory and ethics approvals and MRC CTU at UCL accreditation documents. Sites must complete the COVAC1 Accreditation Form for the trial at the same time as applying for their local approval.

2.1.1 PI'S QUALIFICATIONS & AGREEMENTS

1. The investigators should be qualified by education, training, and experience to assume responsibility for the proper conduct of the trial at their site and should provide evidence of such qualifications through an up-to-date curriculum vitae and/or other relevant documentation requested by the Sponsor, the REC, the IRB, and/or the regulatory authorities.

2. The investigator should be thoroughly familiar with the appropriate use of the investigational products, as described in the protocol, in the current Investigator Brochure, in the product information and in other information sources provided by the Sponsor.

3. The investigator should be aware of, and should comply with, the principles of GCP and the applicable regulatory requirements. A record of GCP training should be accessible for all investigators.

4. The investigator/site should permit monitoring and auditing by the Sponsor, and inspection by the appropriate regulatory authorities.

5. The investigator should maintain a delegation log of appropriately-qualified persons to whom the investigator has delegated significant trial-related duties.

6. The investigator should sign an investigator statement, which verifies that the site is willing and able to comply with the requirements of the trial.

2.1.2 ADEQUATE RESOURCES

1. The investigator should be able to demonstrate a potential for recruiting the required number of suitable subjects within the agreed recruitment period (that is, the investigator regularly treats the target population).

2. The investigator should have sufficient time to properly conduct and complete the trial within the agreed trial period.

3. The investigator should have available an adequate number of qualified staff and adequate facilities for the foreseen duration of the trial to conduct the trial properly and safely.

4. The investigator should ensure that all persons assisting with the trial are adequately informed about the protocol, the investigational product(s), and their trial-related duties and functions.

5. The site should have sufficient data management resources to allow prompt data entry and response to data queries from MRC CTU via a web-based eDC system.

2.1.3 Site Assessment

Each selected clinical trial site must complete the COVAC1 Accreditation Form for the trial, which includes the Investigator Statement, Signature and Delegation of Responsibilities Log, and staff contact details. The Investigator Statement verifies that the site is willing, and able to comply with the requirements of the trial. This will be signed by the Principal Investigator (PI) at the site. In addition and in compliance with the principles of GCP, all site staff participating in the trial must complete the Signature and Delegation of Responsibilities Log and forward this to the MRC CTU at UCL. The MRC CTU at UCL must be notified of any changes to trial personnel and/or their responsibilities. An up-to-date copy of this log must be stored in the Investigator Site File (ISF) at the site and also at the MRC CTU at UCL in the Trial Master File (TMF).

2.2 APPROVAL AND ACTIVATION

The Clinical Trial Authorisation (CTA) for the trial requires that the Medicines and Healthcare products Regulatory Agency (MHRA) be supplied with the names and addresses of all participating site principal investigators, hence it is vital to receive full contact details for all investigators prior to their entering participants.

On receipt of the above documents at the MRC CTU at UCL, written confirmation will be sent to the PI and copied to Imperial College London. The site's pharmacist, or other person delegated by the PI, will also be informed of the site's activation and the vaccines will be dispatched to the named pharmacist, or other person delegated by the PI, in the Accreditation Form documents.

1. The site should conduct the trial in compliance with the protocol as agreed by the Sponsor and the regulatory authority, and which was given favourable opinion by the REC.
2. The PI or delegate should document and explain any deviation from the approved protocol, and communicate this with the trial team at the MRC CTU at UCL.

A list of activated sites can be obtained from the Trial Managers.

2.3 SITE/INVESTIGATOR EXCLUSION CRITERIA

Sites which are unable to store the study vaccine at below –60 °C will be excluded.

3 SELECTION OF PARTICIPANTS

There will be **no exceptions** to eligibility requirements at the time of enrolment. Questions about eligibility criteria should be addressed prior to attempting to enrol the participant.

The eligibility criteria are the standards used to ensure that only medically appropriate participants are considered for this study. Participants not meeting the criteria should not join the study. For the safety of the participants, as well as to ensure that the results of this study can be useful for making treatment decisions regarding other patients with similar health statuses, it is important that no exceptions be made to these criteria for admission to the study.

Participants will be considered eligible for enrolment in this trial if they fulfil all the inclusion criteria and none of the exclusion criteria as defined below.

3.1 PARTICIPANT INCLUSION CRITERIA

1. Healthy adults from the following age ranges:
    a. For the dose escalation and evaluation, aged 18-45 years on the day of screening
    b. For the expanded safety evaluation, aged 18-75 years on the day of screening
2. At similar risk of acquiring SARS-CoV-2 infection to the general population
3. Willing and able to provide written informed consent
4. If female and of childbearing[i] potential, willing to use a highly effective method[ii] of contraception from screening until 18 weeks[iv] after last injection
5. If male and not sterilised, willing to avoid impregnating female partners[iii] from screening until 18 weeks[iv] after last injection
6. Willing to avoid all other vaccines from within 4 weeks before the first injection through to 4 weeks after the second injection[v]
7. Willing and able to comply with visit schedule, complete online diaries and provide samples
8. Willing to grant authorised persons access to his/her trial-related medical record and GP records either directly or indirectly

i A woman will be considered of childbearing potential following menarche and until becoming post-menopausal (no menses for 12 months without an alternative medical cause) unless permanently sterile. Permanent sterilisation methods include hysterectomy, bilateral salpingectomy and bilateral oophorectomy. A post-menopausal state is defined as no menses for 12 months without an alternative medical cause.

ii The following methods are considered highly effective:
- combined (estrogen and progestogen containing) hormonal contraception associated with inhibition of ovulation – oral, intravaginal or transdermal;
- progestogen-only hormonal contraception associated with inhibition of ovulation – oral, injectable or implantable
- intrauterine device (IUD);
- intrauterine hormone-releasing system (IUS);
- bilateral tubal occlusion;
- vasectomised partner, where the vasectomised partner has received medical assessment of the surgical success; and
- sexual abstinence, defined as refraining from heterosexual intercourse – must be the preferred and usual lifestyle of the participant.

iii Through the use of condoms or sexual abstinence (see definition in footnote ii above)

iv Nonclinical studies of saRNAs [48] showed maximal expression of the vaccine immunogen at 7 days post-immunisation, approaching baseline by 3 weeks post-immunisation, with some residual very low expression seen out to 9 weeks. Biodistribution studies with LNP-nCoVsaRNA are planned, but in the absence of data we wish to take a conservative approach to the contraception period, and require an 18-week washout period.

v The exception is the flu vaccine, which participants may have – provided they have it at least 7 days before or after a study vaccination. It is recommended that participants have an up to date vaccination status for any required immunisations.

3.2 PARTICIPANT EXCLUSION CRITERIA

1. Pregnant or lactating
2. Has a significant clinical history, physical finding on clinical examination during screening, or presence of a disease that is active or requires treatment to control it, including cardiac, respiratory, endocrine, metabolic, autoimmune, liver, neurological, oncological, psychiatric, immunosuppresive/immunodeficient or other disorders which in the opinion of the investigator is not compatible with healthy status, increases the risk of severe COVID-19, may compromise the volunteer's safety, preclude vaccination or compromise interpretation of the immune response to vaccine. Individuals with mild/moderate, well-controlled comorbidities are allowed.
3. History of COVID-19 infection[iii]
4. History of anaphylaxis or angioedema
5. History of severe or multiple allergies to drugs or pharmaceutical agents
6. History of severe local or general reaction to vaccination defined as:
    a. **local**: extensive, indurated redness and swelling involving most of the arm, not resolving within 72 hours
    b. **general**: fever ≥39.5 °C within 48 hours; bronchospasm; laryngeal oedema; collapse; convulsions or encephalopathy within 72 hours
7. Ever received an experimental vaccine against COVID-19
8. Receipt of any immunosuppressive agents within 18 weeks of screening by any route other than topical
9. Detection of SARS-CoV-2 antibodies prior to enrolment[i]
10. Detection of antibodies to hepatitis C
11. Detection of antibodies to HIV
12. Grade 1[ii] and above abnormalities in routine laboratory parameters (see **Table 5**) using the FDA toxicity table Toxicity Grading Scale for Healthy Adult and Adolescent Volunteers Enrolled in Preventive Vaccine Clinical Trials, taking account of local laboratory reference ranges. https://www.fda.gov/media/73679/download
13. Participating in another clinical trial with an investigational drug or device, or treated with an investigational drug within 28 days of screening.
14. Has received an immunisation within 28 days of screening

i Only in participants enrolled into the dose escalation and dose evaluation cohorts A1, A2, B1, B2

ii Trace of protein and/or blood on dipstick urinalysis and ALT/AST ≤1.2 x ULN are not exclusion

iii This exclusion criterion applies at all sites with the exception of the St Mary's Hospital site, which will preferentially enrol participants known to have infection-induced SARS-CoV-2 antibodies prior to enrolment

3.3 NUMBER OF PARTICIPANTS

414 participants.

3.4 CO-ENROLMENT GUIDELINES

Co-enrolment in previous or future trials can be reviewed on a case basis (see **Section 4.2**).

3.5 SCREENING PROCEDURES & PRE-RANDOMISATION INVESTIGATIONS

Informed consent to enter into the trial must be obtained from participants after explanation of the aims, methods, benefits and potential hazards of the trial and *before* any trial-specific procedures are

performed or any blood is taken for the trial. Participants must be willing and able to provide informed consent (as detailed in **Section 3.1**). This therefore excludes: persons deprived of their liberty by a judicial or administrative decision, persons under psychiatric care and persons admitted to a health or social institution for purposes other than research; and persons who are the subject of a legal protection measure or who are unable to express their consent.

It must be made completely and unambiguously clear that the participant is free to refuse to participate in all or any aspect of the trial, at any time and for any reason, without incurring any penalty or affecting their treatment.

Signed consent forms must be kept by the investigator and documented on the case report form (CRF) and a copy given to the participant. With consent, the participant's GP will be sent a letter informing them of their patient's intention to participate in the trial, and requesting that they corroborate their patient's medical history. Corroboration may also be obtained via the study team accessing patient's electronic care summaries, GP and other medical records from local systems, or via participants bringing their medical care summaries from their GP to the study team. However, partcipants may be enrolled based on the medical history given at the screening visit only, at the investigator's discretion.

Investigator sites registered with The Overvolunteering Protection System (TOPS) should run checks for the purposes of assessing exclusion criterion 13.

Screening procedures and investigations are listed in **Tables 2 and 3** and covered in more detail in **Section 6**.

4 REGISTRATION & RANDOMISATION

4.1 RANDOMISATION PRACTICALITIES

Further details on the process of randomisation can be found in **Section 9.1**.

Randomisation will only take place at one centre, and only for the first dose evaluation, with a paper randomisation list at the investigator site.

Investigator site staff will know the allocated dose, but participants and laboratory staff will not.

4.2 CO-ENROLMENT GUIDELINES AND REPORTING

Co-enrolment in other clinical trials with an investigational or non-investigational drug or device is not permitted, and individuals who have been treated with an investigational drug within 28 days before the first vaccination will be excluded.

All other co-enrolments should be discussed with MRC CTU at UCL and the Chief Investigator and will be decided on a case by case basis.

5 TREATMENT OF PARTICIPANTS

5.1 INTRODUCTION

Participants in the dose escalation and evaluation will each receive two IM doses of LNP-nCoVsaRNA (0.1, 0.3, 1.0, 2.5, 5 or 10 µg), into the deltoid muscle at Week 0 and Week 4 (see **Table 4**).

Participants in the expanded safety evaluation will each receive two IM doses of LNP-nCoVsaRNA: 1.0 µg at Week 0; and 1.0 µg at Week 4 or 2.5, 5 or 10 µg at Week 14, as recommended by the Trial Steering Committee, into the deltoid muscle (see **Table 4**).

Storage, dispensing, reconstitution and dilution of IMP, the volume for injection and method of administration for each dose level will be described in the COVAC1 Pharmacy Manual.

Table 4: **Vaccine candidates and dispensing schedule**

Study component	Route	Week 0<br><br>Visit 2	Week 4 or 14*<br><br>Visit 5
Dose escalation and evaluation	IM	0.1 µg<br>LNP-nCoVsaRNA	0.1 µg<br>LNP-nCoVsaRNA
	IM	0.3 µg<br>LNP-nCoVsaRNA	0.3 µg<br>LNP-nCoVsaRNA
	IM	1.0 µg<br>LNP-nCoVsaRNA	1.0 µg<br>LNP-nCoVsaRNA
	IM	2.5 µg<br>LNP-nCoVsaRNA	2.5 µg<br>LNP-nCoVsaRNA
	IM	5 µg<br>LNP-nCoVsaRNA	5 µg<br>LNP-nCoVsaRNA
	IM	10 µg<br>LNP-nCoVsaRNA	10 µg<br>LNP-nCoVsaRNA
Expanded Safety	IM	1.0 µg<br>LNP-nCoVsaRNA	1.0 µg or<br>2.5, 5 or 10 µg<br>LNP-nCoVsaRNA

*The timing of the second vaccination in the expanded safety cohort is 4 or 14 weeks after the first vaccination

5.1.1 LNP-NCOVSARNA VACCINE

The LNP-nCoVsaRNA vaccine candidate is manufactured by Polymun Scientific Immunbiologische Forschung GmbH, Klosterneuburg, Austria in accordance with European GMP on behalf of the sponsor, who is also responsible for the product development.

LNP-nCoVsaRNA will be supplied to the sites free-of-charge by the sponsor's distribution partner, PCI Pharma Services. PCI will supply the IMP with COVAC1-specific labels, according to GMP.

The vaccine will be supplied in glass vials at a concentration of 500 µg RNA per mL, with a 100 µL fill volume. It will be supplied with a sterile dilution buffer for reconstitution – phosphate buffered

saline in glass vials with an 18 mL fill volume. The vaccine is a liquid suspension with a white to off-white, appearance.

This vaccine candidate is not classified as a genetically modified organism (GMO).

5.1.2 ADMINISTRATION

Vaccine should be administered intramuscularly in the deltoid muscle of the upper arm using a 23G 1-inch needle. The participant may choose which arm.

Participants will be observed for at least 60 minutes after the injection.

5.1.3 STORAGE

The LNP-nCoVsaRNA vaccine will be stored in a secure, limited access storage area under the specified storage requirements. The vaccine will be stored according to the Pharmacy Manual and the arrangements will be reviewed at site initiation.

5.1.4 DISPENSING

The pharmacist, or other person delegated by the PI, will ensure that the vaccines are dispensed in accordance with the protocol and Pharmacy Manual and local procedures as appropriate. Local working instructions will be reviewed at site initiation, if applicable.

A vaccine accountability log will be kept to record the identification of the participant to whom the vaccine was given and the date they received it. Any damaged or unused vials that are returned will also be documented. The log will be checked during the monitoring visits and at the end of the trial.

5.1.5 SCHEDULE MODIFICATIONS & INTERRUPTIONS

Target visit dates (based on the enrolment date) should be adhered to as far as possible, based on the allowable window (detailed in **Tables 2 and 3**).

The schedule may be interrupted if a participant has symptoms or signs on the day of scheduled injection, and the investigator considers it best to defer the injection. A temperature over 37.5 °C would prevent injection on the day. Clinicians should consult the PI/Medical Delegate if there are any Grade 1 (mild) symptoms or signs listed in **Table 5,** or any symptoms suggestive of COVID-19. The participant will be asked to return for review within the ideal window period of the scheduled injection. Provided the injection is administered during the window period outlined in the paragraph below for missed visits, this will not be a protocol deviation.

The PI or Medical Delegate should interrupt the vaccine schedule and inform MRC CTU at UCL within 24 hours using the safety email (see **section 7**) if there is a confirmed:

(1) Grade 3 (severe) or worse solicited adverse event that has persisted for more than 72 hours regardless of relationship or

(2) other Grade 3 (severe) or worse adverse event that is possibly, probably or definitely related to vaccine or

(3) serious adverse reaction regardless of grade

The Chief Investigator/Delegate may recommend further investigations or referral to an independent expert to support the clinical management of the participant. Such events are highly likely to result in discontinuation, but a decision to resume will be taken by the PI with the participant, and only with the approval of the independent members of the TSC.

Injections for all participants will be paused pending a review of all safety data by the Trial Steering Committee if two participants out of 20, and the equivalent (10%) thereafter develop persisting Grade

3 (severe) adverse reactions within 7 days of immunisation, or any participant experiences a serious adverse reaction at any time. If vaccinations are paused in all participants, a substantial amendment must be submitted to the competent authorities. Likewise, approval from the competent authorities must be sought before the study can be resumed.

Participant visits will continue during a pause. Missed injections will be rescheduled and the remaining study visits rescheduled/repeated accordingly.

All visits will be suspended for any participant with suspected, probable or confirmed COVID-19 until they are out of necessary quarantine and until the diagnosis can be confirmed by appropriate testing.

5.2 HANDLING CASES OF TRIAL MEDICATION OVERDOSE

There are no known important risks associated with LNP-nCoVsaRNA vaccine. There is no known antidote. Participants who are overdosed should be closely monitored and provided with medical support per the investigator's judgment.

5.3 UNBLINDING / UNMASKING

Participants in groups B1 and B2 who do not know the dose they received should request this information from their site team if they are offered an authorised vaccine (see **section 5.9.2.A**)..

5.4 PROTOCOL TREATMENT DISCONTINUATION

In consenting to the trial, participants are consenting to trial treatment, trial follow-up and data collection.

An individual participant may stop injections early or be stopped early for any of the following reasons:

- Pregnancy in the participant
- Unacceptable toxicity that precludes further injections
- Laboratory confirmed COVID-19 infection before or after enrolment
- Intercurrent illness that prevents further injections including emergent conditions that meet the exclusion criteria
- Withdrawal of consent for injections by the participant

A decision by the Medical Delegate to discontinue further injections should be discussed with the local PI, and the Chief Investigator/Medical Delegate should be informed using the expedited reporting method described in **Section 7**. They may recommend additional investigations and/or referral for a specialist opinion.

As participation in the trial is entirely voluntary, a participant may choose to discontinue the trial treatment at any time without penalty or loss of benefits to which they are otherwise entitled. Although the participant is not required to give a reason for discontinuing their trial treatment, a reasonable effort should be made to establish this reason while fully respecting the participant's rights. The implications of withdrawing and how this may impact on the results and interpretation of the trial, will be explained to the participant.

Participants should remain in the trial for the purpose of follow-up and data analysis (unless they withdraw their consent from all stages of the trial, in which case refer to **Section 6.8**).

Data that are already collected from participants who stop follow-up early will be included in the analysis.

5.4.1 Discontinuation Of Injections In All Participants

Protocol planned interruptions and discontinuations will be reported to the Chief Investigator/Medical Delegate who will make a recommendation regarding immediate reporting to the TSC. The MRC CTU at UCL staff responsible for preparing reports for the TSC will forward the clinical report and the dose allocation. All serious adverse reactions will be unexpected and reported on to the authorities (see **section 7.4**) by the staff at MRC CTU at UCL. The TSC may recommend to the Sponsor that injections are discontinued in all participants.

5.5 PARTICIPANT WITHDRAWAL

Participants are free to withdraw from the trial at any time without giving a reason. Those who withdraw will be encouraged to attend a final visit, primarily for safety reasons.

The investigator may at any time withdraw a participant if his/her participation is no longer considered safe or relevant.

The date and reason (if given) for withdrawal must be recorded in the CRF.

If a participant for any reason withdraws or is withdrawn from the trial or has a significant protocol deviation, he/she will be replaced to ensure the per-protocol targets are achieved.

Data and samples collected up to the date of withdrawal will be kept and analysed if appropriate.

5.6 ACCOUNTABILITY & UNUSED VACCINE

The Pharmacist, or other person delegated by the PI, will ensure that all injection products are dispensed in accordance with the protocol, Pharmacy Manual and local procedures, and that records are maintained of receipt, dispensing and destruction of all supplies.

At the end of the trial, IMP accountability will be checked by the designated member of staff responsible for the inventory and by the trial monitors. The Sponsor and the PI will retain copies of the complete IMP accountability records and copies will be provided to the supplier of the vaccines.

All used vials of injections will be destroyed immediately after use in compliance with the instruction manual. The associated packaging will be retained at the investigator site until monitoring is completed and monitors give instruction for their destruction.

Sites will be instructed to either return unused vaccine to the supplier, or to destroy it at site. Following IMP destruction, the pharmacist, or other person delegated by the PI, at each site must complete a certificate of IMP destruction and send it to the MRC CTU at UCL with copies to the Sponsor.

5.7 COMPLIANCE & ADHERENCE

All injections will be administered by site staff and recorded on worksheets and in the eDC system. If an injection is not given within the ideal window (see **Section 5.1.5**), this will be recorded in the eDC system together with the reason. Compliance with the schedule will be reviewed each week at MRC CTU at UCL, and reported to the TMG monthly by the MRC CTU at UCL team.

5.8 TREATMENT DATA COLLECTION

Study staff will collect vaccination information on the worksheets and enter data in the eDC system. Pharmacy staff, or other staff delegated by the PI, will maintain the IMP accountability logs which will be securely stored.

5.9 NON-TRIAL TREATMENT

Non-trial treatments will be reviewed before the first and second injections to ensure that the participant remains eligible and able to receive the vaccine. There are no known interactions between LNP-nCoVsaRNA vaccine and other drugs. All medications taken by participants from screening to the final visit will be recorded in the eDC system.

5.9.1 MEDICATIONS TO BE USED WITH CAUTION

If possible, participants should avoid taking systemic immunosuppressants, which might reduce their immune response to the vaccine

5.9.2 OTHER VACCINATIONS

Vaccination with licensed vaccines should be avoided from 28 days before screening until 28 days after the second injection because they may impact on the assessment of immune response to study vaccine, and it is plausible that the self-amplification process is ongoing for this period of time. The exception is the flu vaccine which may be given, provided it is at least 7 days before or after a study vaccination. The flu vaccine should be administered in the opposite arm to the study vaccine, if possible. Participants in the expanded safety evaluation who choose to delay their second injection, and in whom a flu vaccine is indicated, will be encouraged to have it in the gap between their first and second study vaccinations.

5.9.2.A COVID-19 AUTHORISED VACCINES

COVID-19 authorised vaccines can be administered to trial participants. Reactions to COVID-19 vaccines that contain lipid nanoparticles may be more severe in individuals who have received LNP-nCoVsaRNA, particularly at the higher doses.

Participants should inform the NHS prescriber the dose and dates they received the LNP-nCoVsaRNA vaccine and describe any reactions they had to LNP-nCoVsaRNA.

The date and dose of the authorised COVID-19 vaccine should be recorded in concomitant medications, and adverse events captured in the eDC.

5.9.3 MANAGEMENT OF COVID-19 SYMPTOMS

Participants with suspected COVID-19 symptoms after enrolment but before the second injection will be investigated and injection delayed until a SARS-CoV-2 test result is available. In the event of a positive result the participant will not proceed to the second injection. See **Section 5.1.5** regarding visits windows.

6 ASSESSMENTS & FOLLOW-UP

Potentially eligible participants will be identified through adverts, mainstream, community and social media. They will be able to request written information and be able to make an appointment for a screening visit for further discussion should they prefer to do this in person.

The Delegation Log will determine which members of the study team are authorised to conduct the assessments and procedures described in this section.

6.1 TRIAL ASSESSMENT SCHEDULE

The assessments in clinic and online, samples and volumes to be collected are outlined in **Table 2 Trial Assessment Schedule for the dose escalation and evaluation, both cohorts, and Table 3 Trial Assessment Schedule for the expanded safety cohort**. The maximum blood volume drawn from a participant in the dose escalation or evaluation parts, who completes 52 weeks of follow-up in the study period, is approximately 372 mL. The corresponding volume for participants in the expanded safety evaluation is 175 mL. These volumes do not include the blood volume that would be required if additional diagnostic tests are needed or safety tests have to be repeated. The required volumes per visit together with sample collection and processing guidelines are described in detail in the Laboratory Manual.

The visits in the trial assessment schedule are: a screening visit (Visit 1); an enrolment visit which must take place within 6 weeks of the screening visit and is defined as Week 0 (Visit 2); injection visits at Weeks 0 and 4 or 14 (Visits 2 and 5); safety visits at Weeks 1, 2, 5, 6, 8, 12 and 24 (Visits 3, 4, 6, 7, 8, 9 and 10) and a final visit at Week 52 (Visit 11). The visit windows are provided in **Tables 2 and 3**.

If the second injection which is scheduled at Visit 5 (week 4) is delayed pending a Trial Steering Committee recommendation on the dose to administer, a Visit 4a should be conducted at week 4 instead in order to record any adverse events, collect routine laboratory tests and serology. **The unscheduled visit option should be selected on the Visit Worksheet and eDC screens.** Study staff should maintain contact with participants and one further visit may be required, Visit 4b. This can be conducted over the phone. **The unscheduled visit option should be selected on the Visit Worksheet and eDC screens.** When participants attend for their delayed second injection, study staff should use Visit 5 worksheets and eDC screens which activate the participant diary. **Study staff must not use the Vaccination Visit Worksheet for week 4 or the week 4 eDC screens before the second injection visits.**

Information on vaccine reactions will be solicited directly from participants for one week following each injection using the participant facing tools in the eDC system. They will also be contacted, by telephone, 2 days after each injection.

6.2 PROCEDURES DURING THE SCREENING PERIOD

Screening will take place as close to the planned enrolment as possible, no longer than 6 weeks before enrolment but allowing time for all relevant laboratory assessment to check participant eligibility.

6.2.1 INFORMED CONSENT

Participants will be provided with information about the product, trial design and data collection in writing. They will have the opportunity to ask questions in person or on the phone.

Key points to communicate during the informed consent process:

- That we do not know if the vaccine will prevent SARS-CoV-2 infection
- That pregnancy is to be avoided until 18 weeks after the second injection as the safety of the vaccine is not known
- Although considered very unlikely based on previous coronavirus vaccines, there is a risk that COVID-19 disease could be more severe in vaccine recipients

If they are happy to proceed, they will be asked to indicate their informed consent in writing prior to answering questions about their health and providing samples for the screening investigations. Laboratory investigations or other screening procedures defined in this protocol that have been performed at the local clinical sites(s) for some other purpose (routine NHS visit, healthy volunteer database screening, other research) may be used for the purpose of screening so long as the date they were performed is within the window period defined in this protocol.

A copy will be provided to the participant and one copy kept in the study file according to local procedures.

6.2.2 Eligibility

To assess eligibility, demographic information, a past and current medical history, and details of all current medication will be collected on worksheets and transcribed into the eDC system. Details of contraception to assess the risk of pregnancy arising in the participant/their partner will also be collected.

The screening examination will include weight (kg), height (cm), temperature, blood pressure, pulse, oxygen saturation, inspection of the skin to exclude severe eczema and respiratory, cardio-vascular, abdominal and neurological examination. An assessment of cervical, axillary and inguinal lymph nodes will also be undertaken.

Clinical Investigators should carefully assess participants who have pre-existing conditions and consider the maximum severity in the past, the extent of treatment needed to control the condition at the time of screening, and how long the participant has been stable for on their current treatment. If further investigation is required, including an ECG, or the Clinical Investigator suspects the need for a change in treatment, the participant should be considered ineligible under exclusion criterion number 2 (see **Section 3.2**).

6.2.3 Investigations

Blood will be collected for analysis of routine parameters and processed in the local NHS laboratories for full blood count and biochemistry. The parameters are listed in a footnote to **Tables 2 and 3, and in Table 5**, but additional tests to be conducted at screening are:

- SARS-CoV-2 antibodies (only in participants enrolled into the dose escalation and dose evaluation)
- Hepatitis C antibody
- HIV antibody
- Gamma glutaryl transferase
- Urine dipstick for glucose, blood, WBC, nitrite and protein

Volunteers with Grade 1 abnormalities in haematology, biochemistry or urinalysis parameters at the initial screening visit may have the tests repeated once and may enter the study if the repeat result is normal, at the investigator's discretion. In the event the repeat reveals a new Grade 1 abnormality the volunteer will be considered ineligible.

For female participants of childbearing potential a pregnancy test will be performed by analysis of a urine sample for Human Chorionic Gonadotrophin (HCG).

6.3 PROCEDURES AT ENROLMENT

6.3.1 ELIGIBILITY

Study staff will review any new medical conditions or new medications since the screening data were collected and, for female participants, any changes in contraception. Temperature, blood pressure, pulse, oxygen saturation and inspection of the skin will be repeated at the enrolment visit in case anything has changed. Study staff will repeat the other aspects of the physical examination done at the screening visit if symptoms indicate the need for this (**Section 6.2.2**). All this information will be recorded on the eDC system.

A temperature over 37.5 °C would prevent injection on the day. Clinicians should consult the PI/Medical Delegate if there are any Grade 1 (mild) symptoms or signs listed in **Table 5,** or any symptoms suggestive of COVID-19.

Blood for immunogenicity testing will be collected. Samples will be sent to the central laboratory at Imperial College London for analysis.

For female participants of childbearing potential a pregnancy test will be performed by analysis of a urine sample for Human Chorionic Gonadotrophin (HCG) and a negative result will be confirmed before proceeding to enrolment.

6.3.2 ENROLMENT/RANDOMISATION

Confirmation of eligibility will depend on entering screening data in the eDC system.

6.3.3 INJECTION

Study staff will draw up the injection from the vial and administer it into the deltoid muscle of the participant's choice and record this in the eDC system including the time of injection.

Following injection participants should remain in clinic for at least 60 minutes, in order to assess and record solicited adverse events within 25-60 minutes following injection. Study staff will go through the detail to be added in the online vaccine diary cards with participants and explain how and when to complete these over the following 7 days.

6.4 PROCEDURES FOR ASSESSING SAFETY

Vaccines are associated with a number of well-characterised local, systemic and laboratory reactions referred to as solicited adverse events (**Table 5**). These adverse events will be purposively collected.

Local and systemic assessments will take place on the day of each injection before the injection, and 25-60 minutes after the injection. **Participants should remain in the clinic for at least one hour after each injection.**

Participants will be asked to complete vaccine diary cards on the eDC system to assist collection and grading of local and systemic adverse events that start within 7 days of the injection. They will be advised to contact study staff if any events are Grade 3 (severe), and these will be flagged in the eDC system for immediate attention with a view to organising an early visit.

The information entered will be checked by staff on the phone 2 days after each injection and at the next visit at Weeks 1 and 5.

Blood (~10 mL) for routine safety parameters will be collected at all study visits. If the total bilirubin is elevated, study staff will request a result for conjugated bilirubin in order to grade the abnormality and determine any action to be taken with respect to further investigation and interruption to the vaccine schedule.

Vital signs (BP, HR, oxygen saturation and oral temperature) will be measured at every study visit.

Physical examinations of the injection site, and other body systems if indicated, will be performed on the day of each vaccination, and 1 week after. Symptom-directed physical examinations will be performed at all other follow-up visits.

6.4.1 COVID-19 Symptoms and SARS-CoV-2 Nucleic Acid testing

Participants will be questioned about symptoms associated with COVID-19 at each visit, and will be asked to contact their investigator site if they have symptoms of COVID-19. Symtomatic participants will be asked to provide a swab for SARS-CoV-2 nucleic acid testing.

6.5 PROCEDURES FOR ASSESSING IMMUNE RESPONSES

Vaccine immunogenicity will be assessed through collection of blood samples at every post-screening visit and sent to a central laboratory to evaluate binding and neutralising antibodies according to the Laboratory Analytic Plan. Neutralisation titre will be determined by SARS-CoV-2 pseudovirus-based neutralization assay.

6.5.1 Cellular immune responses

In addition, blood samples will be collected at Weeks 0, 4, 6 and 12 from participants enrolled into the dose escalation and dose evaluation components, and sent to a central laboratory for processing and to assess B and T cellular responses in gamma interferon ELISpot assays and through flow cytometry using intracellular cytokine staining, according to the Laboratory Analytic Plan.

Table 5: **Solicited adverse events**

TYPE	EVENT
Local AEs<br>(injection site)	Pain<br>Tenderness/Discomfort<br>Redness/Erythema<br>Swelling (soft or hard)
Systemic Clinical AEs following injections	Temperature<br>Chills/shivers<br>Myalgia/flu-like general muscle aches<br>Arthralgia<br>Fatigue<br>Headache<br>Nausea<br>Vomiting

Systemic Laboratory AEs	Creatinine<br>ALT/AST<br>Alkaline phosphatase<br>Total bilirubin<br>Non-fasting glucose	Haemoglobin<br>Total White Cell Count<br>Neutrophils<br>Lymphocytes<br>Platelets

6.6 OTHER ADVERSE EVENTS

Other adverse events will be collected through an open question about health at every study visit, and during telephone visits at 2 days after each vaccination. Study staff will record the diagnosis or the symptoms if a diagnosis is not apparent, the date of onset and the date of resolution if appropriate. If the event is ongoing, it may be appropriate to conduct a symptom-directed examination.

Events should be graded according to the FDA: Toxicity Grading Scale for Healthy Adult and Adolescent Volunteers Enrolled in Preventive Vaccine Clinical Trials, taking account of local laboratory reference ranges. https://www.fda.gov/media/73679/download

6.7 INCIDENTAL FINDINGS

An incidental finding is one that has potential health or reproductive importance which is discovered unexpectedly in the course of conducting research, but is unrelated to the purpose or aims of the study – e.g. an abnormal laboratory safety test result. Investigator sites should follow their own local procedures. Depending on the nature of the finding, the subject might have to be withdrawn or vaccinations discontinued, per Sections 5.4 and 5.5, and his/her GP informed if consent to do so is received from the participant.

6.8 EARLY STOPPING OF FOLLOW-UP

If a participant chooses to discontinue injections, they should always be followed up for safety and pregnancy events providing they are willing. If they do not wish to remain on trial follow-up, however, their decision must be respected and the participant will be withdrawn from the trial completely. The CTU should be informed of this in writing using the appropriate documentation.

Medical data collected in the trial will be kept for research and analysis purposes are pseudo-anonymised. Consent for future use of stored samples already collected can be refused when leaving the trial early (but this should be discouraged and should follow a discussion).

Participants do not need to withdraw from the trial if they receive an authorised COVID-19 vaccine. They should be encouraged to continue as per trial schedule if possible. If the reason for withdrawal does relate to receiving an authorised COVID-19 vaccine, and the participant has not yet completed Visit 9, then the procedures and investigations listed in Visit 9 in the schedule should be collected, namely:

- Vital signs, concomitant medications, adverse events including any symptoms of COVID-19 and symptom directed examination
- Routine laboratory safety parameters, SARS-CoV2 swab if indicated, serology
- Urine pregnancy test
- PBMC collection if in cohort A2 or B2 (A1 and B1 have all completed Visit 9)

6.9 PARTICIPANT TRANSFERS

Participant transfers are not anticipated, but every effort will be made for the participant to be seen at another participating trial site should this happen. The participant data in the eDC system will be made available to the new site and the participant will need to sign a new consent form. Once this has been done, the new site will take over responsibility; until this has been done, responsibility for the participant lies with the original site.

6.10 LOSS TO FOLLOW-UP

Study staff will make every effort to contact participants who do not attend their scheduled visits. At least three attempts will be made to contact the participant during the period from enrolment through to Week 52 (Visit 11).

Participants who are lost to follow-up after at least three attempts to contact them will be able to return to follow-up if they make contact at a later date before trial closure.

Participants will be followed up in the long-term through usual mechanisms and with the appropriate consent, which may include flagging via NHS Digital, or similar approaches.

6.11 COMPLETION OF PROTOCOL FOLLOW UP

The trial will be closed when all participants have made their final follow-up visit and assessments are completed including those to determine resolution of any adverse events, the data entered into the database and checked and the database locked.

7 SAFETY REPORTING

The principles of GCP require that both investigators and Sponsors follow specific procedures when notifying and reporting adverse events or reactions in clinical trials. These procedures are described in this section of the protocol. **Section 7.1** lists definitions, **Section 7.3** gives details of the investigator responsibilities and **Section 7.4** provides information on CTU responsibilities.

7.1 DEFINITIONS

The definitions of the EU Directive 2001/20/EC Article 2 based on the principles of GCP apply to this trial protocol. These definitions are given in **Table 6**.

Table 6: **Definitions**

TERM	DEFINITION
Adverse Event (AE)	Any untoward medical occurrence in a patient or clinical trial subject to whom a medicinal product has been administered including occurrences that are not necessarily caused by or related to that product.
Adverse Reaction (AR)	Any untoward and unintended response to an investigational medicinal product related to any dose administered.
Unexpected Adverse Reaction (UAR)	An adverse reaction, the nature or severity of which is not consistent with the information about the medicinal product in question set out in the Summary of Product Characteristics (SPC) or Investigator Brochure (IB) for that product.
Serious Adverse Event (SAE) or Serious Adverse Reaction (SAR) or Suspected Unexpected Serious Adverse Reaction (SUSAR)	Respectively any adverse event, adverse reaction or unexpected adverse reaction that:<br>▪ Results in death<br>▪ Is life-threatening*<br>▪ Requires hospitalisation or prolongation of existing hospitalisation**<br>▪ Results in persistent or significant disability or incapacity<br>▪ Consists of a congenital anomaly or birth defect<br>▪ Is another important medical condition***

*The term life-threatening in the definition of a serious event refers to an event in which the patient is at risk of death at the time of the event; it does not refer to an event that hypothetically might cause death if it were more severe, for example, a silent myocardial infarction.

**Hospitalisation is defined as an inpatient admission, regardless of length of stay, even if the hospitalisation is a precautionary measure for continued observation.

*** Medical judgement should be exercised in deciding whether an AE or AR is serious in other situations. The following should also be considered serious: important AEs or ARs that are not immediately life-threatening or do not result in death or hospitalisation but may jeopardise the subject or may require intervention to prevent one of the other outcomes listed in the definition above; for example, a secondary malignancy, an allergic bronchospasm requiring intensive emergency treatment, seizures or blood dyscrasias that do not result in hospitalisation or development of drug dependency.

7.1.1 MEDICINAL PRODUCTS

An investigational medicinal product is defined as the tested investigational medicinal product (IMP) and the comparators used in the study (EU guidance ENTR/CT 3, April 2006 revision).

Adverse reactions include any untoward or unintended response to the vaccines (IMP). Reactions should be reported appropriately.

7.1.2 ADVERSE EVENTS

Adverse Events include:

- An exacerbation of a pre-existing illness
- An increase in frequency or intensity of a pre-existing episodic event or condition
- A condition (even though it may have been present prior to the start of the trial) detected after trial drug administration
- Continuous persistent disease or a symptom present at baseline that worsens following administration of the study treatment

7.1.3 EXEMPTED ADVERSE EVENTS

Adverse Events do not include:

- Medical or surgical procedures; the condition that leads to the procedure is the adverse event
- Pre-existing disease or a condition present before treatment that does not worsen
- Hospitalisations where no untoward or unintended response has occurred, e.g., elective cosmetic surgery, social admissions

7.2 OTHER NOTABLE EVENTS

Notable adverse events which impact on the injection schedule and therefore require expedited (within 24 hours of the investigator becoming aware of the event) reporting whether or not they meet the serious criteria include:

- Grade 3 (severe) and above solicited adverse events which last more than 72 hours and Grade 3 and above laboratory adverse events that are confirmed on repeat testing if possible
- Any adverse event leading to a clinical decision to interrupt or discontinue the injection schedule
- Pregnancy within 18 weeks of an injection (see **section 7.2.1**)

7.2.1 PREGNANCY

Pregnancy is not an adverse event. However, it is a reportable event and should be reported to MRC CTU at UCL within 24 hours of the investigator becoming aware of a pregnancy. The pregnancy should be reported using the Notable Event form and Pregnancy form in the eDC system. All pregnancies, including those occurring in female partners of male participants will be followed up to collect information about the outcome. Collecting information about the outcome from partners is subject to consent from the partner.

In the event of a pregnancy in a female participant, injections will be discontinued.

All Notable events, including pregnancy must be reported immediately using the eDC system. Under no circumstances should this exceed 24 hours following knowledge of the event.

7.3 INVESTIGATOR RESPONSIBILITIES

All non-serious AEs and ARs, whether expected or not, should be recorded in the participants' medical notes and reported on the AE form in the eDC system on the day of the visit from screening through to the last study visit for each subject.

SAEs and SARs should be notified to the sponsor (Imperial College London) and MRC CTU at UCL within 24 hours of the investigator becoming aware of the event via the eDC system Under no circumstance should this exceed 24 hours following knowledge of the SAE or SAR. Immediate reporting should allow the sponsor to take the appropriate measures to address any potential new risks in the trial.

Participants must be followed up until clinical recovery is complete and laboratory results have returned to normal or baseline, or until the event has stabilised. Follow-up should continue after completion of the protocol if necessary.

7.3.1 INVESTIGATOR ASSESSMENT

7.3.1.A Seriousness

When an AE or AR occurs, the investigator responsible for the care of the patient must first assess whether or not the event is serious using the definition given in **Table 4**. If the event is serious, then an SAE details must be entered in the eDC system within 24 hours. If the event is not a SAE but meets the notable event criteria (see **section 7.2**) complete a Notable Event Form and forward the report within 24 hours via the same mechanism.

7.3.1.B Severity or Grading of Adverse Events

The severity of all AEs and/or ARs (serious and non-serious) in this trial should be graded using the FDA: Toxicity Grading Scale for Healthy Adult and Adolescent Volunteers Enrolled in Preventive Vaccine Clinical Trials.

7.3.1.C Causality

The investigator must assess the causality of all events in relation to the trial therapy using the definitions in **Table 7**. There are five categories: unrelated, unlikely, possible, probable, and definitely related. If the causality assessment is unrelated or unlikely to be related, the event is classified as an unrelated AE. If the causality is assessed as possibly, probably or definitely related, then the event is classified as an AR.

The investigator will also be asked to record concomitant medications and to assess the relationship of the event to each of these, including authorised COVID-19 vaccines. If a serious adverse event is considered related to a concomitant medication, the investigator should report this to the MHRA via a yellow card and inform the CTU via email mrcctu.covac@ucl.ac.uk.

7.3.1.D Expectedness

If there is at least a possible involvement of any trial treatment given to the participants, the Chief Investigator, on behalf of the Sponsor, will make an initial assessment of the expectedness of each SAR. An unexpected serious adverse reaction is one not previously reported in the current Reference Safety Information, which is in the LNP-nCoVsaRNA Investigator's Brochure. A SAR that is more frequent or more severe than stated in the reference safety information would also be considered to be unexpected. If an SAR is assessed as being unexpected, it becomes a SUSAR.

7.3.1.E Notification

The MRC CTU at UCL and Imperial College London should be notified of all SAEs within 24 hours of the investigator becoming aware of the event via the eDC system. Under no circumstance should this exceed 24 hours following knowledge of the event.

Investigators should report all SAEs occurring from the time of screening until the last protocol visit via the safety email.

SARs and SUSARs must be notified to the MRC CTU at UCL and Imperial College London until trial closure.

Any subsequent events that may be attributed to injections should be reported to the national ethics committee and regulatory body as per national guidelines using the yellow card system for the MHRA.

Table 7: **Assigning Type of AE Through Causality**

RELATIONSHIP	DESCRIPTION	SAE TYPE
Definitely	There is clear evidence to suggest a causal relationship and other possible contributing factors can be ruled out.	AR
Probable	There is evidence to suggest a causal relationship and the influence of other factors is unlikely.	AR
Possible	There is some evidence to suggest a causal relationship (for example, because the event occurs within a reasonable time after administration of the trial medication). However, the influence of other factors may have contributed to the event (for example, the patient's clinical condition, other concomitant treatments).	AR
Unlikely	There is little evidence to suggest that there is a causal relationship (for example, the event did not occur within a reasonable time after administration of the trial medication). There is another reasonable explanation for the event (for example, the patient's clinical condition, other concomitant treatment).	Unrelated AE
Unrelated	There is no evidence of any causal relationship	Unrelated AE

If an AE is considered to be related to trial treatment and the injections are interrupted please refer to **Section 5.2.6 and Section 5.4**.

7.3.2 NOTIFICATION PROCEDURE

The minimum criteria required for reporting an SAE are the participant's trial number and date of birth, name of investigator reporting, the event, and why it is considered serious.

The SAE details should be entered in the eDC system by an investigator (a clinician named on the Signature List and Delegation of Responsibilities Log, who is responsible for the participant's care), with due care being paid to the grading and causality as outlined above. In the absence of the responsible investigator, the data should be entered by a member of the site trial team. The responsible investigator should subsequently check the SAE details on the eDC system, make changes as appropriate, as soon as possible.

Follow up details on the SAE should be entered in the eDC system as they become available. Extra, annotated information and/or copies of test results may be provided separately and can be emailed securely (e.g. via Galaxkey). The participant must be identified by trial number, month and year of birth and initials only. The participant's name should not be used on any correspondence and should be deleted from any test results.

Staff should follow their institution's procedure for local notification requirements.

SAE REPORTING
Please report all SAEs and Notable Events via the eDC system within 24 hours of becoming aware of an SAE/NE
If you have any issues with entering the SAE/NE or have any questions please email
mrcctu.covac@ucl.ac.uk

7.4 CTU RESPONSIBILITIES

Medically-qualified staff at the CTU and/or the Chief Investigator (or a medically-qualified delegate) will review all SAE reports received in the eDC system. The causality assessment given by the local investigator at the hospital cannot be overruled; in the case of disagreement, both opinions will be provided in any subsequent reports.

The CTU is undertaking the duties of trial Sponsor and is responsible for the reporting of SUSARs and other SARs to the regulatory authorities (MHRA) and the research ethics committee, as appropriate. Fatal and life-threatening SUSARs must be reported to the competent authority within 7 days of the CTU becoming aware of the event; other SUSARs must be reported within 15 days.

The CTU will also keep all investigators informed of any safety issues that arise during the course of the trial.

The CTU, on behalf of the Sponsor, will submit Annual Safety Reports in the form of a Developmental Safety Update Report (DSUR) to Competent Authorities (Regulatory Authority and Ethics Committee) and the final clinical study report.

8 QUALITY ASSURANCE & CONTROL

8.1 RISK ASSESSMENT

The Quality Assurance (QA) and Quality Control (QC) considerations have been based on a formal Risk Assessment, which acknowledges the risks associated with the conduct of the trial and how to address them with QA and QC processes. QA includes all the planned and systematic actions established to ensure the trial is performed and data generated, documented and/or recorded and reported in compliance with the principles of GCP and applicable regulatory requirements. QC includes the operational techniques and activities done within the QA system to verify that the requirements for quality of the trial-related activities are fulfilled. This Risk Assessment has been reviewed by the CTU's Research Governance Committee (RGC) and has led to the development of a Data Management Plan (DMP), Safety Reporting Plan and Monitoring Plan which will be separately reviewed by the Quality Management Advisory Group (QMAG).

8.1.1 SAFETY AND RIGHTS OF PARTCIPANTS

The pre-clinical data available for LNP-nCoVsaRNA for three species suggest that this product will be similar to licensed vaccines without adjuvant and cause mild-moderate reactions that are transient. However, this is a first in human study and therefore there will be a sentinel cohort with one individual allocated to each of the three doses in one centre only. There will be immediate review of notable and serious adverse events with onward reporting to the TSC, and clear indications for pausing injections in individuals and the trial.

It will be necessary to hold personal contact details in order to collect the data on reactogenicity and COVID-19 symptoms. The justification for this will be explained in the PIS as well as the storage and destruction of these data after the study. Explicit consent will be collected for this, as well as long term flagging in the ONS and NHS databases for COVID-19 disease.

8.1.1.A Authorised COVID-19 vaccines

The recommended minimum gap between LNP-nCoVsaRNA and an authorised COVID-19 vaccine is 28 days, in part to complete the safety and immunogenicity assessments but also because it is plausible that the self-amplifcation continues during this period. There were no toxicity signals in a pre-clinical experiment conducted in a research laboratory in mice combining the ChAdOx vaccine and LNP-nCoVsaRNA. There are no experiments combining more than one lipid nanoparticle RNA vaccines. Lipid nano-particles used to deliver drugs intravenously have been associated with complement activation related pseudoallergy (CARPA) but this is usually observed with the first intravenous administration. Nonetheless, individuals who have experienced possible allergic reactions following LNP-nCoVsaRNA may be at increased risk of an allergic reaction to another LNP-RNA vaccine and they should be advised to discuss this with the NHS prescriber accordingly. Similarly it makes sense that participants who experienced moderate or severe reactions to LNP-nCoVsaRNA after their first and/or second vaccines will experience similar reactions following an authorised LNP-RNA vaccine. They will be reminded of the advice provided to manage reactions in COVAC1 and asked to report these events at their next visit. Where a second injection has not been given for a reason considered related to the trial vaccine (individual stopping rule) the participant should be advised to discuss this with the NHS team delivering the NHS COVID-19 immunisation programme before receiving an authorised COVID-19 vaccine.

Please see **Section 10.2.1** on ethical considerations.

8.1.2 Project Design and Reliability

There is considerable interest in participating, and retention in previous early phase studies at the participating centres has always been good. Immune responses will be assessed in a single laboratory and couriered using a reliable courier.

8.1.3 Project Management and Governance

Imperial College London has worked with the CTU on prevention trials including vaccines for over 25 years and therefore no issues are anticipated in managing the trial between the two institutions.

8.2 CENTRAL MONITORING AT CTU

CTU staff will review electronic data for errors and missing data points.

Other essential trial issues, events and outputs will be detailed in the Monitoring Plan that is based on the trial-specific Risk Assessment.

8.3 ON-SITE AND REMOTE MONITORING

The frequency, type and intensity for routine monitoring and the requirements for triggered monitoring will be detailed in the Monitoring Plan. This plan will also detail the procedures for review and sign-off. Remote or self- monitoring will be utilised through the course of the trial. Site staff may be asked to scan and send anonymised sections of a participant's medical record to the CTU for remote verification or asked to complete a form to confirm compliance with protocol procedures.

8.3.1 Direct Access to Participant Records

Participating investigators should agree to allow trial-related monitoring, including audits, ethics committee review and regulatory inspections by providing direct access to source data and documents as required. Participants' consent for this must be obtained.

8.3.2 Confidentiality

Investigator sites, CTU and Sponsor must follow the principles of the UK Data Protection Act.

All personal data leaving the investigator site(s) will be pseudonymised in that it will bear the participants' trial ID and not readily identifiable information such as name or contact details. The investigator site(s) will maintain a participant identification list which links trial IDs to participants' names and NHS numbers (NHS sites only).

The exceptions to the above, mentioned also in **Section 8.1.1**, are:

1. Details such as first name, telephone number and email address will be entered by investigator site staff into the eDC system, so that the system can communicate directly with participants, to send reminder messages for example. These details will be encrypted and stored in the eDC system such that only the relevant investigator site staff can view them. Sponsor and MRC CTU staff will be unable to view them.
2. NHS numbers will be transferred to MRC CTU to facilitate long-term follow-up of COVID-19-related health outcomes, using NHS databases.

9 STATISTICAL CONSIDERATIONS

9.1 METHOD OF RANDOMISATION

Only a single site (ICRF) will participate in the first randomised dose-finding study (0.1, 0.3 and 1.0 µg dose levels). Participants will be allocated in a 1:1:1 ratio to the three different doses based on block randomisation.

For the second dose evaluation the doses to be evaluated are 2.5, 5 and 10 µg and this study will be conducted at two or more sites. Participants will be allocated in a 1:1:1 ratio to the three different doses, but this will not be individually randomised. Participants and laboratory staff will not know the dose but the clinic staff will.

9.2 OUTCOME MEASURES

The outcome measures are:

- Solicited local injection site reactions starting within 7 days of administration of the vaccine: pain, tenderness, erythema, swelling
- Solicited systemic reactions starting within 7 days of administration of the vaccine: pyrexia, fatigue, myalgia, headache, chills, arthralgia
- Unsolicited adverse reactions (ARs) throughout the study period (including serious ARs)
- Serious Adverse Events
- Unsolicited adverse events throughout the study period
- The titre of serum neutralising antibodies 2 weeks after the second vaccination in the SARS-CoV-2 pseudovirus-based neutralization assay
- The titre of vaccine-induced serum IgG binding antibody responses to the SARS-CoV-2 S glycoprotein 2 weeks after the first and second vaccinations

The exploratory outcome measures are:

- Cell-mediated vaccine-induced immune responses measured by T- and B- cell ELISpot in participants in the dose escalation and evaluation parts
- Cell-mediated vaccine-induced immune responses measured by flow cytometry and intracellular cytokine staining in participants in the dose escalation and evaluation parts
- The profile of class and sub-class of antibody response
- Laboratory markers of infection and infection-induced immunity

9.3 SAMPLE SIZE

Safety

In the first cohort, at least 39 (4+35), 39 (4+35), and 242 (42 + 200) participants are expected to receive at least one vaccination at a dose of 0.1 µg, 0.3 µg and 1.0 µg respectively. Safety analyses will include all dose groups but the most robust data will be generated on the 1.0 µg dose.

Table 8 shows the probability of observing at least 1, 2 , 3, or 4 adverse reactions in the 1.0 µg group, by the true frequency. The study is reasonably powered to detect a true frequency of between 1/50 to 1/100 or higher.

Table 8: **Probability (%) of observing X or more adverse reactions by true frequency (First Cohort)**

Number of notable adverse reactions	**True frequency**			
	**1/50**	**1/100**	**1/200**	**1/500**
1 or more	99	91	70	38
2 or more	95	69	34	8
3 or more	86	43	12	1
4 or more	71	22	3	<1

In the second cohort, safety outcomes will be compared between the 2.5, 5, and 10 µg dose groups. In addition: (a) analyses will be conducted of the dose-response relationship across all evaluated doses, combining data across the first and second cohorts, even though this comparison is not strictly randomised (b) the expanded safety cohort will provide additional information on safety although some participants may receive a different dose at their first and second vaccinations.

Immunogenicity

In the first cohort, a total of 105 participants will be included in the randomised dose evaluation of immunogenicity, 35 at each dose. The primary analysis will compare SARS-CoV-2 neutralization IC_{50} values at 6 weeks (2 weeks after the booster at 4 weeks). Data on mice and recovered COVID-19 patients indicate a standard deviation of approximately 1.5 for neutralization $\log_{10}$ IC_{50} values. The sample size calculation for the dose evaluation part of this study is based on testing the slope co-efficient in a linear regression, using the $\log_{10}$ scale for dose. The study has 97% statistical power (2α=0.05) to detect a true difference of 0.7 ($\log_{10}$ IC_{50} scale) between adjacent doses (corresponding to a slope of 1.4), 91% power a difference of 0.6, 79% power a difference of 0.5, and 59% power a difference of 0.4.

Data on the first and second cohorts will be considered together to explore the overall relationship between immunogenicity and dose, using the same primary endpoint. As it is suspected that the relationship will be non-linear over this wide dose range (100-fold), evaluation of statistical power is not possible without strong assumptions.

9.4 INTERIM MONITORING & ANALYSES

The trial will be overseen by a Trial Steering Committee (TSC), which will include membership independent of the Trial Management Group (TMG). An Independent Data Monitoring Committee (IDMC) will be set up for the efficacy trial but not for COVAC1. The reason for this is the need for real-time assessment of the safety data. The TSC will be informed by the Chief Investigator when the first participant receives the first injection of 0.1, 0.3 and 1µg, and a report of 7 day reactogenicity will be provided when the first 15 individuals have completed their Week 1 visit. The TSC will receive weekly

progress reports, and will be asked to review the accumulating safety and immunogenicity data in order to recommend which doses(s) should be used to boost the safety expansion cohort, and which dose should be taken forward to efficacy testing. Any adverse reactions that lead to interruption in the schedule for an individual or plan for enrolments described in Section 1.5 will be immediately reported to the TSC. Sites will be requested to notify the Chief Investigator and Project Lead at CTU within 24 hours of any adverse reaction that is a cause for concern as described in **Section 5.1.5** These reports will be shared with the Trial Steering Committee immediately. If there are 2 out of 20 participants (10% thereafter) who experience similarly severe adverse reactions, vaccinations will be paused in all individuals and the competent authority informed. The TSC will be asked to review the accumulating safety data from all participants and make a recommendation about resuming vaccinations.

It is anticipated that serological assays will be conducted at two or three time-points during the course of the trial and might lead to changes to the protocol as recommended by the TSC.

9.5 ANALYSIS PLAN

A full analysis plan will be provided in a separate Statistical Analysis Plan.

Primary safety analyses will be based on all participants who receive at least one dose of vaccine. Safety endpoints will be compared between the different dose groups. The frequency of adverse events will be tabulated by grade and MedDRA System Organ Class and treatment group, and MedDRA Preferred Term and treatment group. Groups will be compared using Fisher's exact test in terms of the proportion ever experiencing an event in each MedDRA System Organ Class.

Primary analyses of immunological responses at 6 weeks will be based on all participants who receive both the initial vaccination and the booster at 4 weeks. These analyses will exclude participants who are identified as COVID-19 seropositive at baseline (either retrospectively, or those enrolled at the St Mary's Hospital site with known previous SARS-CoV-2 infection). Data will also be censored on participants who acquire SARS-CoV-2 infection (virologically-confirmed) during follow-up or who receive an authorised COVID-19 vaccine.

Immunological responses at all time-points will be compared between the younger and older age groups using cross-sectional analyses and longitudinal multilevel models.

10 REGULATORY & ETHICAL ISSUES

10.1 COMPLIANCE

10.1.1 REGULATORY COMPLIANCE

The trial will be conducted in compliance with the approved protocol, the Declaration of Helsinki 1996, the principles of Good Clinical Practice (GCP) as laid down by the ICH topic E6 (R2), Commission Clinical Trials Directive 2005/28/EC* with the implementation in national legislation in the UK by Statutory Instrument 2004/1031 and subsequent amendments, General Data Protection Regulation and the UK Data Protection Act 2018, and the UK Policy Framework for Health and Social Care Research.

*Until the Clinical Trials Regulation EU No 536/2014 becomes applicable, the trial will be conducted in accordance with the Clinical Trials Directive as implemented in the UK statutory instrument. When the directive is repealed on the day of entry into application of the Clinical Trial Regulation the trial will work towards implementation of the Regulation (536/2014) following any transition period.

10.1.2 SITE COMPLIANCE

An agreement will be in place between the site and the CTU, setting out respective roles and responsibilities (see **Section 12 - Finance**).

The site should inform CTU as soon as they are aware of a possible serious breach of compliance, so that the CTU can report this breach if necessary within 7 days as per the UK regulatory requirements. For the purposes of this regulation, a 'serious breach' is one that is likely to affect to a significant degree:

- The safety or physical or mental integrity of the subjects in the trial, or
- The scientific value of the trial

10.1.3 DATA COLLECTION & RETENTION

Worksheets, clinical notes and administrative documentation should be kept in a secure location (for example, locked filing cabinets in a room with restricted access) and held for a minimum of 25 years after the end of the trial. During this period, all data should be accessible, with suitable notice, to the competent or equivalent authorities, the Sponsor, and other relevant parties in accordance with the applicable regulations. The data may be subject to an audit by the competent authorities. Medical files of trial participants should be retained in accordance with the maximum period of time permitted by the hospital, institution or private practice.

Note: EU Clinical Trial Regulation 536/2014 states the following:
"Unless other Union law requires archiving for a longer period, the sponsor and the investigator shall archive the content of the clinical trial master file for at least 25 years after the end of the clinical trial. However, the medical files of subjects shall be archived in accordance with national law."

10.2 ETHICAL CONDUCT

10.2.1 ETHICAL CONSIDERATIONS

Please see **Section 8.1** for the risks identified for the safety and rights of participants, which include the risk of unexpected serious adverse reactions and the need to collect and hold personal data. The main ethical considerations, and mitigations, are described below:

- The trial is in healthy volunteers so all visits required by the trial are in addition to their usual lifestyle and therefore are a burden. Vaccine trials are (necessarily) relatively lengthy, so

participation represents a significant time commitment. The requirement to attend all study visits within specified windows might be an inconvenience to some volunteers if their circumstances change during the trial. Furthermore, attending visits may increase the risk to participants of SAR-CoV-2 infection above that if the individual had stayed at home. Participants will be instructed to follow national guidance for the reduction of risk appropriate to their demographic.

- Participants may be overly motivated to participate based on their desire to gain protection from COVID-19. Great care will be taken to inform participants of the risks and benefits in taking part in this study, and to ensure that they continue to follow national guidance for the reduction of risk appropriate to their demographic.
- Fainting may occur around the time of vaccine injection or blood sampling, particularly in those who strongly dislike needles. To minimise this risk, participants will be asked to recline or lie down during those procedures.
- Blood tests can sometimes cause bruising and soreness of the arms or, very rarely, a blockage of a vein or a small nerve injury which can cause numbness and pain.
- The vaccine has not been tested for safety in pregnancy and might harm an unborn child. The contraception requirements for women participants of child-bearing potential might be a burden. Male participants will also be required to use contraception with female partners capable of becoming pregnant.
- The collection of sensitive or personal data will be undertaken only by staff trained in GCP and in the trial protocol.
- Participants will be informed of the results either at a seminar, or by email or on the phone. The PIS explains the hope to publish the results in medical journals, and present them at international conferences, and clarifies that participants will not be named in any of these or identified in any other way.
- Participants might believe that they are entitled to a share of potential future profits from commercialisation of the vaccine. The PIS explains that this is not the case.
- The confidentiality of participants' personal information is described in **Section 8.3.2**.
- The trial has some medication restrictions which might be a burden: non-trial vaccines received within 28 days before or after any trial vaccination are not allowed (excepting flu vaccine – see Section 5.9.2); systemic immunosuppressive agents, such as a corticosteroid, may not be administered during the trial; dermal steroids are allowed but not if applied to the IM injection site.
- Based on other vaccines against different diseases, including those that use synthetic mRNA, the side effects are expected to be mild to moderate, short-lived reactions at the injection site such as discomfort, warmth, redness and swelling. Short-lived systemic symptoms such as fatigue, general malaise and headache are expected very commonly (more than 1 in 10 people). Less common reactions (expected in fewer than 1 in 10 people, but in more than 1 in 100) include chills, muscle pain, rash, and injection site itching.
- Rare reactions (less than 1 in 1,000 people, but more than 1 in 10,000) include enlarged lymph nodes, high fever (≥40 °C), hypersensitivity (exaggerated reaction to the vaccine), urticaria (raised, itchy skin rash), and granuloma (area of inflammation in the skin) or sterile abscess (lump) at the injection site.
- Very rarely (less than 1 in 10,000 people) vaccines may cause convulsions with fever, drowsiness, and macrophagic myofasciitis (a rare muscle disease).

- Severe allergic reactions to vaccines are very rare (less than 1 in 10,000 people). However, appropriate medical treatment and trained staff will be available in case this occurs.
- The theoretical risk of ADE is discussed in **Section 1.2.4**.

These risks will be carefully explained so that participants know what is involved and what to expect in the way of side effects.

Participants will be monitored closely during the trial, to identify as early as possible any problems so that they can be handled appropriately. They will be given 24-hour phone numbers in case they wish to speak to a doctor.

Because this is a first-in-human trial, there will be a dose escalation cohort with sentinel participants at each dose level, who will be vaccinated 2 days before any others. The dose escalation and evaluation plan for the first and second cohorts is detailed in Section 1.5.

The rules for pausing and discontinuing vaccinations in individuals and all participants in the trial have been clearly defined.

Participants will be paid for their time, inconvenience and travel expenses: £50 per scheduled visit, paid as a lump sum at the end of participation. This includes any travel expenses they might incur. Volunteers who attend screening but who are not enrolled will not receive payment. If their participation ends early, participants will be paid for the number of visits they've attended.

10.2.2 Favourable Ethical Opinion, HRA Approval, and Host Site Permission

Following Main REC approval and Health Research Authority (in England) approvals and before initiation of the trial at each clinical site, the protocol, all informed consent forms, and information materials to be given to the prospective participant will be submitted to each Trust's Research and Development (R&D) office. In Wales, Scotland and Northern Ireland, the R&D office will be asked to give approval. In England, the R&D office will be asked to confirm capacity and capability. Similarly, management permission will be sought from non-NHS sites. Any substantial amendments will be reviewed by the sponsor before being submitted for approval by the REC and HRA. Local R&D/management permission will be sought for amendments, as required.

The study has been developed with Participant and Public Involvement (PPI) to ensure that its design is feasible and acceptable to potential participants, and to ensure its outcomes and potential impact are relevant to the population who may benefit from its results. PPI also helps to ensure transparency and accountability throughout this research. PPI activity will continue for the duration of the study, including dissemination of study results.

The rights of the participant to refuse to participate in the trial without giving a reason must be respected. After the participant has entered into the trial, the clinician must remain free to give alternative treatment to that specified in the protocol, at any stage, if he/she feels it to be in the best interest of the participant. The reason for doing so, however, should be recorded; the participant will remain within the trial for the purpose of follow-up and for data analysis. Similarly, the participant must remain free to change their mind at any time about the vaccinations and trial follow-up without giving a reason and without prejudicing his/her further treatment.

10.3 COMPETENT AUTHORITY APPROVALS

This protocol will be reviewed by the national competent or equivalent authority (MHRA, FDA etc.) as appropriate in each country where the trial will be run.

This is a Clinical Trial of an Investigational Medicinal Product (IMP) as defined by the EU Directive 2001/20/EC. Therefore, a Clinical Trial Authorisation is required in the UK. Any substantial amendments will be reviewed by the sponsor before being submitted for authorisation by the MHRA. Local R&D/management permission will be sought for amendments, as required.

The EudraCT number for the trial is: 2020-001646-20.

The progress of the trial and safety issues will be reported to the competent authority, regulatory agency or equivalent in accordance with local requirements and practices in a timely manner.

Safety reports, including expedited reporting of SUSARS, will be submitted to the competent authority in accordance with each authority's requirements in a timely manner.

10.4 TRIAL CLOSURE

The end of trial is defined in **Section 6.11**.

11 INDEMNITY

Imperial College London holds negligent harm and non-negligent harm insurance policies which apply to this study.

Imperial College London cannot take legal responsibility for a prescription that is not in the trial protocol.

12 FINANCE

Imperial College London (ICL) is in receipt of a grant award which will fund the trial. ICL will enter into agreement with University College London to formalise the arrangements around MRC CTU's role in the trial. ICL and UCL will enter into tripartite agreements with each investigator site to formalise the arrangements around their roles in the trial. ICL will enter into agreement with PCI Pharma Services to formalise the arrangements around PCI's role in the trial. These agreements will include financial aspects.

13 OVERSIGHT & TRIAL COMMITTEES

There are a number of committees involved with the oversight of the trial. These committees are detailed below.

13.1 TRIAL MANAGEMENT TEAM (TMT)

A Trial Management Team (TMT) will be formed comprising the Chief Investigator, the Scientific Lead, and members of the MRC Clinical Trials Unit (CTU) and Imperial Clinical Research Facility (ICRF) who have a coordinating role. The TMT will be responsible for preparing and reviewing the central data monitoring reports including the weekly safety reports, and for onward reporting to the TMG and TSC.

13.2 TRIAL MANAGEMENT GROUP (TMG)

A Trial Management Group (TMG) will be formed comprising the Chief Investigator, other lead investigators (clinical and non-clinical) including the Principal Investigators from each site, and members of the MRC Clinical Trials Unit (CTU) and a PPI contributor. The TMG will be responsible for the day-to-day running and management of the trial. It will convene approximately every two weeks in the first instance and usually by tele/video conference. The full details can be found in the TMG Charter.

13.3 TRIAL STEERING COMMITTEE (TSC)

The Trial Steering Committee (TSC) has membership from the TMG plus independent members, including the Chair and a PPI contributor. The role of the TSC is to provide overall supervision for the trial, in particular to provide advice on safety and immune responses. The ultimate decision for the continuation of the trial lies with the TSC. Further details of TSC functioning are presented in the TSC Charter.

13.4 INDEPENDENT DATA MONITORING COMMITTEE (I[DMC])

There is no plan to form an IDMC for this study because there is no placebo and a need for real-time monitoring of safety data. Only participants and laboratory staff are blind to the dose administered in the dose evaluation cohorts. Therefore we propose to submit regular reports of safety to the independent members of the TSC, and to forward any safety concerns should they emerge (see **Sections 5.1.5, 5.4.1 and 9.4**).

13.5 PATIENT AND PUBLIC INVOLVEMENT ADVISORY GROUPS

PPI contributors will be individuals that are identified toreflect the wider community from which participants are drawn.

Any issues identified by the PPI contributors will be forwarded to the TMT as they arise and onward to TMG if this is necessary in order to address the issue (see Section 14.6). If there are issues that the TMT or TMG identify that require consultation with the PPI contributors, this may be done via a survey or web forum.

Additional information can be found in the PPI policy.

13.6 ROLE OF STUDY SPONSOR

Imperial College London, as represented by the Imperial Joint Clinical Research Office, is the sponsor of the trial and maintains overall responsibility for the conduct and reporting of the trial.

14 PATIENT AND PUBLIC INVOLVEMENT

Participant and Public Involvement (PPI) in research is defined by INVOLVE (an advisory group established by the NIHR) as research being carried out 'with' or 'by' members of the public rather than 'to', 'about' or 'for' them.

COVAC1 has a comprehensive PPI policy which involves participants, the public and key community stakeholders throughout the life-cycle of this trial and the transition to clinical efficacy testing. At the core of the policy will be the commitment to active PPI during the study, building partnerships with participants, the public and national stakeholders to shape decisions about the research.

The goal is to gain a broad and diverse range of perspectives rather than achieve representativeness of specific population groups.

The policy will describe

- the models of PPI that will be used in the COVAC1 trial
- the use of PPI through the research cycle of the trial
- the framework for assessing the impact of PPI
- the procedures for supporting PPI

14.1 POTENTIAL IMPACT OF PPI

PPI will provide the study team with insight into the best way to engage participants, explain the study accurately, and minimise the burden of data collection. This is likely to optimise recruitment and retention to the trial as well as accuracy and completeness of data. Every effort will be made to ensure that the PIS, vaccine reaction and symptom diaries are comprehensible to all ages, races, ethnic groups including those whose first language is not English.

14.2 IDENTIFYING PPI CONTRIBUTORS

Individuals who have previously participated in early phase trials, or have experience of prevention trials, or have experience of clinical trial committee work, or have a strong involvement in PPI, and who may be able to reflect on the concerns and issues of the target population for this trial and the efficacy trial will be invited to become PPI contributors and provided with the CTU induction pack.

14.3 REPORTING AND EVALUATING IMPACT OF PPI

An impact assessment form will be used to collate the purpose, implementation and response for each PPI activity throughout the course of the trial. If recommendations from PPI activity are not implemented, the reason why will be documented on the assessment form.

15 PUBLICATION AND DISSEMINATION OF RESULTS

The preparation of a manuscript for publication in a peer-reviewed professional journal or an abstract for presentation, oral or written, to a learned society or symposium will be discussed on the TMG calls and with the PPI Advisory Group. Details of dissemination can be found in the trial specific communication plan.

Authorship will reflect work done by the investigators and other personnel involved in the analysis and interpretation of the data, in accordance with generally recognised principles of scientific collaboration.

Details regarding the roles and responsibilities and timelines are contained in the Clinical Trial Agreement.

16 DATA AND/OR SAMPLE SHARING

Data will be shared according to the CTU's controlled access approach, based on the following principles:

- No data should be released that would compromise an ongoing trial or study.
- There must be a strong scientific or other legitimate rationale for the data to be used for the requested purpose.
- Investigators who have invested time and effort into developing a trial or study should have a period of exclusivity in which to pursue their aims with the data, before key trial data are made available to other researchers.
- The resources required to process requests should not be under-estimated, particularly successful requests which lead to preparing data for release. Therefore adequate resources must be available in order to comply in a timely manner or at all, and the scientific aims of the study must justify the use of such resources.
- Data exchange complies with Information Governance and Data Security Policies in all of the relevant countries.

Data will be available for sharing. Researchers wishing to access COVAC1 data should contact the Trial Management Group in the first instance.

17 PROTOCOL AMENDMENTS

The protocol current at the start of the trial was v3.0, dated 11 Jun 2020.

In amendment AM002, the protocol was updated to v4.0, dated 06 Jul 2020, in order to:

- increase the screening window;
- clarify the post-vaccination visit windows;
- add ALP to footnote 2 to Table 1;
- fix a formatting error in Section 1;
- allow the enrolment of SARS-CoV-2 antibody positive participants at the St Mary's site;
- clarify that a full physical exam is not required at Week 0.

In amendment AM004, the protocol was updated to v5.0, dated 12 Aug 2020, in order to:

- incease the window for the Week 4 vaccination visit;
- clarify that participants must remain in clinic for at least 60 minutes post-vaccination.

In amendment AM006, the protocol was updated to v6.0, dated 09 Sep 2020, in order to:

- Add rationale and process for higher dose escalation and evaluation
- Add Table 1 to clarify the groups to be studied
- Increase the total number of participants to up to 420
- Add two visits (4a and 4b) for those participants in the safety evaluation who choose to delay their second vaccination
- Change the timeline for the subsequent efficacy trial
- Clarify eligibility for participants with Grade 1 abnormalities at screening
- Update the total approx. blood volume collected from participants, because the sample volume for cellular immunogenicity has decreased slightly
- Make the flu vaccine an acceptable concomitant medication - provided it is at least 7 days before or after a study vaccination.
- Remove mention of a COVID-19 online symptom diary - it was not possible to develop this system in time.
- Clarify the randomisation arrangements in the dose evaluations
- Fix of various minor inconsistencies and typographical errors

In amendment AM008, the protocol was updated to v7.0, dated 06 Nov 2020, in order to:

- Add, in Section 19, a sub-study of immunogenicity in participants recruited at the St Mary’s site only
- Fix two errors in Table 3: the timing of Visit 9 in footnote 1; add back in the serum immunogenicity sampling which had been deleted in error (no change to per-visit blood draws)
- Fix a formatting error in Section 9.1/9.2

In amendment AM009, the protocol was updated to v8.0, dated xx Dec 2020, in order to :

- Update section 5.3 on unblinding and unmasking it in the rial
- Add, a new sub-section 5.9.2 to provide guidance on the use of authorised COVID-19 vaccines
- Update to section 6.8 to provide additional information on early stopping of follow-up
- Updated guidance on causality assessment in section 7.1.3C,
- Add, a new sub-section 8.1.1.A on authorised vaccines
- Update to section 9.5 on the analysis plan
- Clarify indemnity provision in section 11

18 REFERENCES

[1] Herbert AS, et al. Venezuelan equine encephalitis virus replicon particle vaccine protects nonhuman primates from intramuscular and aerosol challenge with ebolavirus. J Virol. 2013;87:4952-64.

[2] Li W, et al. Characterization of Immune Responses Induced by Ebola Virus Glycoprotein (GP) and Truncated GP Isoform DNA Vaccines and Protection Against Lethal Ebola Virus Challenge in Mice. J Infect Dis. 2015;212 Suppl 2:S398-403.

[3] Cashman KA, et al. Enhanced Efficacy of a Codon-Optimized DNA Vaccine Encoding the Glycoprotein Precursor Gene of Lassa Virus in a Guinea Pig Disease Model When Delivered by Dermal Electroporation. Vaccines. 2013;1(3):262-77.

[4] Grant-Klein RJ, et al. Codon-optimized filovirus DNA vaccines delivered by intramuscular electroporation protect cynomolgus macaques from lethal Ebola and Marburg virus challenges. Hum Vaccin Immunother. 2015;11:1991-2004.

[5] Shedlock DJ, et al. Induction of broad cytotoxic T cells by protective DNA vaccination against Marburg and Ebola. Mol Ther. 2013; 21:1432-44.

[6] Grant-Klein RJ, et al A multiagent filovirus DNA vaccine delivered by intramuscular electroporation completely protects mice from ebola and Marburg virus challenge. Hum Vaccin Immunother. 2012;8:1703-6.

[7]. Li L, Petrovsky N. Molecular mechanisms for enhanced DNA vaccine immunogenicity. Expert Rev Vaccines. 2016;15:313-29.

[8] Sahin U, et al. mRNA-based therapeutics. Nat Rev Drug Discov. 2014;13:759.

[9] Alberer M, et al. Safety and immunogenicity of a mRNA rabies vaccine in healthy adults: an open-label, non-randomised, prospective, first-in-human phase 1 clinical trial. Lancet. 2017;390:1511-1520.

[10] Feldman RA, et al. mRNA vaccines against H10N8 and H7N9 influenza viruses of pandemic potential are immunogenic and well tolerated in healthy adults in phase 1 randomized clinical trials. Vaccine. 2019;37:3326-3334.

[11] Kis Z, et al. Emerging Technologies for Low-Cost, Rapid Vaccine Manufacture. Biotechnol J. 2019;14:e1800376.

[12] Wrapp D, et al. Cryo-EM structure of the 2019-nCoV spike in the prefusion conformation. Science. 2020;367:1260-1263.

[13]. Ljungberg K, and Liljeström P. Self-replicating alphavirus RNA vaccines. Expert Rev Vaccines. 2015;14:177-94.

[14]. Ulmer JB, Mansoura MK, Geall AJ. Vaccines 'on demand': science fiction or a future reality. Expert Opin Drug Discov. 2015;10:101-106.

[15]. Brito LA, et al. Self-amplifying mRNA vaccines. Adv Genet. 2015;89:179-233.

[16]. Erwin-Cohen RA, Porter AI, Pittman PR, Rossi CA, DaSilva L. Human transcriptome response to immunization with live-attenuated Venezuelan equine encephalitis virus vaccine (TC-83): Analysis of whole blood. Hum Vaccin Immunother. 2017;13:169-179

[17]. Bernstein DI, et al.Randomized, double-blind, Phase 1 trial of an alphavirus replicon vaccine for cytomegalovirus. Vaccine. 2009;28:484-93

[18] Crank MC, et al. A proof of concept for structure-based vaccine design targeting RSV in humans. Science. 2019;365:505-509.

[19]. Slovin SF, et al. A phase I dose escalation trial of vaccine replicon particles (VRP) expressing prostate-specific membrane antigen (PSMA) in subjects with prostate cancer. Vaccine. 2013;31:943-9.

[20] Bernstein DI, et al. Randomized, double-blind, Phase 1 trial of an alphavirus replicon vaccine for cytomegalovirus in CMV seronegative adult volunteers. Vaccine. 2009;28:484-93.

[21] Démoulins T, et al. Polyethylenimine-based polyplex delivery of self-replicating RNA vaccines. Nanomedicine. 2016;12:711-722.

[22] Vogel AB, et al. Self-Amplifying RNA Vaccines Give Equivalent Protection against Influenza to mRNA Vaccines but at Much Lower Doses. Mol Ther. 2018;26:446-455.

[23] Hoy SM. Patisiran: First Global Approval. Drugs. 2018;78:1625-1631.

[24] NCT04283461. Safety and Immunogenicity Study of 2019-nCoV Vaccine (mRNA-1273) to Prevent SARS-CoV-2 Infection

[25] Lokugamage KG, Yoshikawa-Iwata N, Ito N, Watts DM, Wyde PR, et al. Chimeric coronavirus-like particles carrying severe acute respiratory syndrome coronavirus (SCov) S protein protect mice against challenge with SCoV. Vaccine 2008;26: 797–808.

[26] Yasui F, Kai C, Kitabatake M, Inoue S, Yoneda M, et al. Prior immunization with severe acute respiratory syndrome (SARS)-associated coronavirus (SARS-CoV) nucleocapsid protein causes severe pneumonia in mice infected with SARS-CoV. J Immunol 2008; 181: 6337–6348.

[27] Bolles M, Deming D, Long K, Agnihothram S, Whitmore, et al. A double-inactivated severe acute respiratory syndrome coronavirus vaccine provides incomplete protection in mice and induces increased eosinophilic proinflammatory pulmonary response upon challenge. J Virol 2011;85: 12201–12215.

[28] Tseng CT, et al. Immunization with SARS coronavirus vaccines leads to pulmonary immunopathology on challenge with the SARS virus. PLoS One. 2012;7(4):e35421.

[29] Castilow EM, Olson MR, Varga SM. Understanding respiratory syncytial virus (RSV) vaccine-enhanced disease. Immunol Res 2007;39: 225–239.

[30] Collins PL, Graham BS. Viral and host factors in human respiratory syncytial virus pathogenesis. J Virol 2008;82: 2040–2055.

[31] Deming D, Sheahan T, Heise M, Yount B, Davis N, et al. Vaccine efficacy in senescent mice challenged with recombinant SARS-CoV bearing epidemic and zoonotic spike variants. PLoS Medicine 2006;3: 2359-2375.

[32] Takada, A. & Kawaoka, Y. Antibody-dependent enhancement of viral infection: molecular mechanisms andin vivo implications. Rev. Med. Virol. 2003;13, 387–398.

[33] MASCOLA, J. R. et al. Summary Report: Workshop on the Potential Risks of Antibody-Dependent Enhancement in Human HIV Vaccine Trials. AIDS Res. Hum. Retroviruses 1993;9, 1175–1184.

[34] Jaume, M. et al. Anti-Severe Acute Respiratory Syndrome Coronavirus Spike Antibodies Trigger Infection of Human Immune Cells via a pH- and Cysteine Protease-Independent Fc R Pathway. J. Virol. 2011;85, 10582–10597.

[35] Wan, Y. et al. Molecular Mechanism for Antibody-Dependent Enhancement of Coronavirus Entry. J. Virol. 2020;94. pii: e02015-19.

[36] Wang, Q. et al. Immunodominant SARS Coronavirus Epitopes in Humans Elicited both Enhancing and Neutralizing Effects on Infection in Non-human Primates. ACS Infect. Dis. 2, 2016;361–376.

[37] Yip, M. S. et al. Antibody-dependent enhancement of SARS coronavirus infection and its role in the pathogenesis of SARS. Hong Kong Med. J. = Xianggang yi xue za zhi 2016;22, 25–31.

[38] Yip, M. et al. Antibody-dependent infection of human macrophages by severe acute respiratory syndrome coronavirus. Virol. J. 2014;11, 82.

[39] Yang, Z. -y. et al. Evasion of antibody neutralization in emerging severe acute respiratory syndrome coronaviruses. Proc. Natl. Acad. Sci. 2005;102, 797–801'

[40] Wang, S.-F. et al. Antibody-dependent SARS coronavirus infection is mediated by antibodies against spike proteins. Biochem. Biophys. Res. Commun. 2014;451, 208–214.

[41] Liu, L. et al. Anti-spike IgG causes severe acute lung injury by skewing macrophage responses during acute SARS-CoV infection. JCI insight 2019;4.

[42] Kam, Y. W. et al. Antibodies against trimeric S glycoprotein protect hamsters against SARS-CoV challenge despite their capacity to mediate FcγRII-dependent entry into B cells in vitro. Vaccine 2007;25, 729–740.

[43] Luo, F. et al. Evaluation of Antibody-Dependent Enhancement of SARS-CoV Infection in Rhesus Macaques Immunized with an Inactivated SARS-CoV Vaccine. Virol. Sin. 2-14;33, 201–204.

[44] McLellan JS, et al. Structure-based design of a fusion glycoprotein vaccine for respiratory syncytial virus. Science. 2013;342(6158):592-8.

[45] Hadinegoro SR, et al M; CYD-TDV Dengue Vaccine Working Group. Efficacy and Long-Term Safety of a Dengue Vaccine in Regions of Endemic Disease. N Engl J Med. 2015 Sep 24;373(13):1195-206. doi: 10.1056/NEJMoa1506223. Epub 2015 Jul 27. PubMed PMID: 26214039

[46] Kentner AC, Miguelez M, James JS, Bielajew C. Behavioral and physiological effects of a single injection of rat interferon-alpha on male Sprague-Dawley rats: a long-term evaluation. Brain Res. 2006;1095:96-106.

[47] Pepini T, Pulichino AM, Carsillo T, Carlson AL, Sari-Sarraf F, Ramsauer K, Debasitis JC, Maruggi G, Otten GR, Geall AJ, Yu D, Ulmer JB, Iavarone C. Induction of an IFN-Mediated Antiviral Response by a Self-Amplifying RNA Vaccine: Implications for Vaccine Design. J Immunol. 2017 May 15;198(10):4012-4024. doi: 10.4049/jimmunol.1601877.

[48] Geall AJ, et al. Nonviral delivery of self-amplifying RNA vaccines. Proc Natl Acad Sci U S A. 2012;109(36):14604-14609.

19 APPENDIX 1 – COVAC1 SUB-STUDY

19.1 SUMMARY OF SUB-STUDY

SUMMARY INFORMATION TYPE	SUMMARY DETAILS
**Sub-study title**	Characterisation of the cellular immune responses to vaccination against SARS-CoV-2 in individuals with and without existing antibodies to the virus.
**Study Design**	This is a sub-study of COVAC1 to explore in-depth the immune responses to the LNP-nCoVsaRNA vaccine in a subset of individuals already recruited into COVAC1. Participants are based at the St Mary's Hospital site and have so far received one 1 µg dose of vaccine and are due to receive a second dose. They fall into two categories (i) negative for SARS-CoV-2 antibodies at baseline, and (ii) positive for SARS-CoV-2 antibodies at baseline.
**Setting**	Imperial College Clinical Trials Centre, St Mary's Hospital, Imperial College Healthcare NHS Trust, London
**Type of Participants to be Studied and Justification**	Healthy adults aged 18-75 years who have already consented to take part in COVAC1 and received one dose of vaccine at the St Mary's Hospital site (PI Alan Winston-O'Keefe). This includes two distinct groups of individuals that can be directly compared:<br>• Negative for SARS-CoV-2 antibodies at baseline<br>• Positive for SARS-CoV-2 antibodies at baseline
**Sponsor**	Imperial College London
**Interventions to be Compared**	LNP-nCoVsaRNA vaccine in individuals positive and negative for SARS-CoV-2 antibodies at baseline.
**Objectives**	• To explore SARS-CoV-2 vaccine induced cellular immune responses in individuals naïve to SARS-CoV-2<br>• To explore SARS-CoV-2 vaccine induced cellular immune responses in Individuals recovered from natural SARS-CoV-2 infection<br>• To compare immune responses to LNP-nCoVsaRNA in those with and without SARS-CoV-2 antibodies at baseline
**Exploratory Outcome Measures**<br>**These are in addition to those of the main study protocol**	These may include but are not limited to in the following ways:-<br>• Cell-mediated vaccine-induced immune responses measured by T- and B-cell ELISpot<br>• Cell-mediated vaccine-induced immune responses measured by flow cytometry, activation induced marker assays and intracellular cytokine staining<br>• B cell receptor sequence prior to the 2nd vaccination dose, at 1 week post 2nd vaccination dose and at 52 weeks.
**Number of Participants to be Studied**	Up to 37
**Duration**	This sub-study will start at visit 5 of the main COVAC1 trial (on the day of the 2nd vaccine dose), and end at visit 11 of the main trial (38 weeks after the 2nd vaccine dose)
**Funder**	The Wellcome Trust
**Chief Investigator**	Dr Katrina Pollock

19.2 SUB-STUDY ASSESSMENT SCHEDULE

Table 9: **Sub-study Assessment Schedule for participants**

**Main Trial Visit**	**V5**		**V6**	**V7**	**V8**		**V11**
**Sub-study Visit**	**V1**		**V2**	**V3**	**V4**		**V5**
Visit Type (site or telephone)	Site		Site	Site	Site		Site
Main Trial Week	14		15	16	18		52
Sub-study Week	0		1	2	4		38
Windows (days)	−14 to +28		0 to +2	−2 to +2	−4 to +4		−36 to +7
Blood for cellular immunogenicity assays	48 mL		48 mL	48 mL	48 mL		48 mL
Total blood volume in addition to that collected in the main trial	48 mL		48 mL	48 mL	48 mL		48 mL

19.3 LAY SUMMARY

Severe acute respiratory syndrome coronavirus-2 (SARS-CoV-2) is the virus responsible for the COVID-19 pandemic. It is not currently known what protection is achieved after natural infection with SARS-CoV-2. COVAC1 is testing a new vaccine against SARS-CoV-2. If successful, this vaccine could be used to offer protection against infection with SARS-CoV-2 or the disease (COVID-19) or help prevent re-infection in those that have already had it.

This sub-study of COVAC1 is looking specifically at how well, and for how long, the vaccine activates the immune system in both people who have never had the virus before and in people who have had the virus and recovered. This activation may provide protection against infection and re-infection with the virus.

Participants have already received one dose of the vaccine and are due to have a second dose. In order to see how well the immune system is responding, participants in the sub-study will need to give five additional blood samples: one before the second vaccination, and four afterwards. The sub-study samples will be collected at the same times as those for the main trial, and at the St Mary's Hospital study centre only.

19.4 BACKGROUND

19.4.1 INTRODUCTION

Coronaviruses are single stranded RNA viruses that are causative for SARS (epidemic in 2003, SARS-CoV), MERS (2012, MERS-CoV) and now COVID-19 (SARS-CoV-2). To date there are no licensed vaccines for SARS, MERS or COVID-19. It is not known what protection might be gained post infection with SARS-CoV-2 and cases of re-infection have been reported [1–5], so it is important that not only a vaccine is developed, but that this can be given safely to boost the immune response in those who have recovered from infection, should this be necessary.

COVAC1 is assessing the safety and immunogenicity of a self-amplifying ribonucleic acid (saRNA) vaccine encoding the S glycoprotein of SARS-CoV-2 and started recruitment in June 2020. At the St Mary's site 37 participants have been recruited and have received a 1 µg dose of saRNA vaccine at Week 0. Around half of these participants have had a previous infection with SARS-CoV-2 and had detectable antibodies against the N-protein (using the Abbott platform) at baseline prior to vaccination. All participants at the St Mary's site have had safety bloods and serum samples taken for analysis of the serological response to the S-protein. However, in order to evaluate the cellular immune responses of these individuals additional blood for peripheral blood mononuclear cells (PBMCs) is needed. This will allow us to explore in detail SARS-CoV-2-specific T- and B- cell immunity post-vaccination over time in those previously infected compared with those naive to SARS-CoV-2 at baseline.

19.4.2 POTENTIAL RISKS AND MITIGATIONS

In this sub-study we require an additional 48 mL of blood for PBMC at five timepoints. This gives a total of an additional 240 mL of blood throughout the duration of the sub-study, and increases the total COVAC1 donation to 415 mL over 1 year, which is within a safe volume for donation and is less than the maximum volume of blood allowed for donation by the blood bank.

The risks to the participant of venepuncture are pain and bruising following the procedure. The participant may also feel faint due to a vaso-vagal response or hypotension, and in this case the venepuncture will cease and the patient will be laid flat until recovery.

19.4.3 POTENTIAL BENEFITS

There is no direct benefit to the participants of this sub-study, however a potential benefit is to aid with the understanding of the immune response to the vaccine and any protection this may give.

19.4.4 RATIONALE FOR SUB-STUDY

It is not currently known what protection might be gained post natural infection with SARS-CoV-2 but based on findings from other coronaviruses, it is likely that any protection offered will wane with time and that these individuals may benefit from vaccination to boost their immune response. This sub-study will allow us to explore in detail SARS-CoV-2-specific T- and B- cell immunity post-vaccination over time in those previously infected compared with those naive to SARS-CoV-2 at baseline.

19.4.5 OBJECTIVES

The stated objectives, in addition to those of the main study are:

- To explore SARS-CoV-2 vaccine induced cellular immune responses in individuals naïve to SARS-CoV-2

- To explore SARS-CoV-2 vaccine induced cellular immune responses in Individuals recovered from natural SARS-CoV-2 infection
- To compare immune responses to LNP-nCoVsaRNA in those with and without SARS-CoV-2 antibodies at baseline

19.4.6 Outcome Measures

These may include but are not limited to celluar and humoral immune responses measured in the following ways:-

- Cell-mediated vaccine-induced immune responses measured by T- cell ELISpot (prior to 2nd vaccination, at 2 weeks post vaccination and at 52 weeks), and memory B-cell ELISpot (prior to 2nd vaccination, at 4 weeks post vaccination and at 52 weeks)
- Cell-mediated vaccine-induced immune responses measured by flow cytometry, activation induced marker assays and intracellular cytokine staining
- B cell receptor sequencing prior to the 2nd vaccination dose, at 1 week post 2nd vaccination dose and at 52 weeks

As humoral immunity is largely being addressed in the main study, this sub-study will focus on measuring cell-mediated immunity to SARs-CoV-2 infection in those receiving the study vaccine.

19.4.7 Study Design

Participants have already been recruited into COVAC1 at the St Mary's Hospital site and received a 1 µg dose of vaccine. They are due to return at visit 5 for the second dose. Participants that consent to this sub-study will have 48 millilitres of additional blood taken at the following main trial visits: visit 5, visit 6, visit 7, visit 8, visit 11 (Table 9).

19.5 SELECTION OF SITES/CLINICIANS

St Mary's site was chosen as this is the only site where participants who were positive for SARS-CoV-2 antibodies at baseline were recruited. The PI at the site is Prof Alan Winston-O'Keefe.

19.6 SELECTION OF PARTICIPANTS

Participants will be considered eligible for enrolment in this trial if they fulfil all the inclusion criteria as defined below.

19.6.1 Participant Inclusion Criteria

1. Recruited into COVAC1 at St Mary's site
2. Willing and able to comply with visit schedule and provide samples
3. Able to give informed consent

19.6.2 Number of ParTiCIPants

Maximum 37 participants.

19.7 ASSESSMENTS & FOLLOW-UP

The Delegation Log will determine which members of the study team are authorised to conduct the assessments and procedures described in this section.

19.7.1 TRIAL ASSESSMENT SCHEDULE

The samples and volumes to be collected are outlined Table 9. The maximum blood volume drawn from a participant who completes 52 weeks of follow-up in the main trial study period, and also participates in the sub-study, is approximately 415 mL. This volume does not include the blood volume that would be required if additional diagnostic tests are needed or safety tests have to be repeated. The total is well within the volumes which are allowed for blood donations in the UK: 470 mL every 16 weeks (females) or every 12 weeks (males).

The main trial visits in the trial assessment schedule requiring additional blood for PBMCs are: 2nd vaccination visit (visit 5; Week 14), weeks 15 (visit 6), Week 16 (visit 7), Week 18 (visit 8) and week 52 (Visit 11).

These main trial visits map to the sub-study timepoints as follows:

Main COVAC1 Trial Visit	Main COVAC1 Trial Week	Sub-Study Week
5	14	0
6	15	1
7	16	2
8	18	4
11	52	38

19.7.2 INFORMED CONSENT

Participants will be provided with information about the sub-study in writing. They will have the opportunity to ask questions in person or on the phone.

Key points to communicate during the informed consent process:

- Additional blood is required for 5 of the remaining scheduled site visits.

If they are happy to proceed, they will be asked to indicate their informed consent in writing.

A copy will be provided to the participant and one copy kept in the study file according to local procedures.

19.7.3 PROCEDURES FOR ASSESSING IMMUNE RESPONSES

Blood samples will be processed at laboratories at Imperial College London.

Cellular immune responses

B and T cellular responses will be assessed through standard immunology techniques, including ELISpot assays, flow cytometry, activation induced marker assays and intracellular cytokine staining.

19.7.4 EARLY STOPPING OF FOLLOW-UP

If a participant wishes not to remain in the sub-study they are free to withdraw consent.

Medical data collected in the sub-study will be kept for research purposes and are pseudo-anonymised. Consent for future use of stored samples already collected can be withdrawn when leaving the sub-study early. following a discussion.

19.7.5 Loss to Follow-up

Study staff will make every effort to contact participants who do not attend their scheduled visits. At least three attempts will be made to contact the participant during the period from enrolment through to Week 52 (Main Trial Visit 11).

Participants who are lost to follow-up after at least three attempts to contact them will be able to return to follow-up if they make contact at a later date before sub-study closure.

19.7.6 Completion of sub-study follow up

The sub-study will be closed when all participants have made their final follow-up visit.

19.8 DATA MANAGEMENT, RISK ASSESSMENT AND MONITORING

19.8.1 Data Management

MRC CTU will not be responsible for data collection, analysis or reporting of sub-study results. The sub-study will not use the MRC CTU eDC system. Instead, paper CRFs will be used to capture the required data, which will be very limited in scope: demographics, and dates of blood samples. Dates of sub-study blood samples will be entered directly into the paper CRF, which is therefore the source document in this instance. A copy of the CRFs will be provided by the site to Imperial College London. The main data outputs of the study will be directly from laboratory analysers, in the form of Excel files.

19.8.2 Risk Assessment

Safety and Rights of Partcipants

The sub-study does not significantly increase the risk to participants in the main COVAC1 trial. Their safety and rights will be safeguarded by processes established in the main COVAC1 trial.

Please see **Section 19.10.2** on ethical considerations for the sub-study.

Project Design and Reliability

The participants in the sub-study will also be participants in the main COVAC1 trial. Participant retention in the main trial has been excellent, and we expect that to continue and be the same for the sub-study.

Project Management and Governance

The sub-study is part of an Imperial College London PhD project co-supervised by ICL academics with many years of experience of supervising successful PhD projects. The co-supervisors include the COVAC1 CI, Dr Katrina Pollock, and the Scientific Lead, Prof Robin Shattock. MRC CTU will not be involved in project management or governance of the sub-study.

19.8.3 Monitoring

MRC CTU will not be responsible for monitoring the sub-study. The responsibility for this lies with ICL, and is delegated to the CI – who may delegate this to an appropriately trained member of staff at the Imperial CRF.

All sub-study consent forms will be checked for completeness and for assurance that consent was given before any sub-study blood samples were collected.

100% of the demographics data collected in the CRFs will be verified against source data in the participants’ electronic healthcare records.

100% of dates and subject IDs in the outputs from immunology laboratory analyser will be cross-checked against the CRFs for one key cellular immune response analysis.

Monitoring reports will be sent to the sponsor, and to the MRC CTU for addition to the TMF.

Direct Access to Participant Records

Participating investigators should agree to allow trial-related monitoring, including audits, ethics committee review and regulatory inspections by providing direct access to source data and documents as required. Participants’ consent for this was obtained in the main COVAC1 trial consent form.

Confidentiality

The investigator site and Sponsor must follow the principles of the UK Data Protection Act.

All personal data leaving the investigator site will be pseudo-anonymised in that it will bear the participants' sub-study ID (which will be the same as the main COVAC1 trial ID) and not readily

identifiable information such as name or contact details. The investigator site will maintain a participant identification list which links sub-study IDs to participants' names and NHS numbers.

19.9 STATISTICAL CONSIDERATIONS

19.9.1 SAMPLE SIZE

This sub-study is investigating exploratory outcomes. All participants recruited at St Mary's site will be asked if they wish to participate in this sub-study (n=37).

19.9.2 ANALYSIS PLAN

Immunological responses will be compared between the SARS-CoV-2 seropositive and seronegative participants.

19.10 REGULATORY & ETHICAL ISSUES

19.10.1 COMPLIANCE

Regulatory Compliance

Requirements are equivalent to those for the main COVAC1 trial – see Section 10.1.1. The sub-study will be conducted in compliance with the approved protocol, the Declaration of Helsinki 1996, the principles of Good Clinical Practice (GCP) as laid down by the ICH topic E6 (R2), Commission Clinical Trials Directive 2005/28/EC with the implementation in national legislation in the UK by Statutory Instrument 2004/1031 and subsequent amendments, General Data Protection Regulation and the UK Data Protection Act 2018, and the UK Policy Framework for Health and Social Care Research. This is the

Site Compliance

An agreement is in place between the site and the CTU, setting out respective roles and responsibilities for the main COVAC1 trial (see **Section 12 - Finance**).

The site should inform CTU as soon as they are aware of a possible serious breach of compliance, so that the CTU can report this breach if necessary within 7 days as per the UK regulatory requirements. For the purposes of this regulation, a 'serious breach' is one that is likely to affect to a significant degree:

- The safety or physical or mental integrity of the subjects in the trial, or
- The scientific value of the trial

Data Collection & Retention

Requirements are equivalent to those for the main COVAC1 trial – see Section 10.1.3. CRFs, clinical notes and administrative documentation should be kept in a secure location (for example, locked filing cabinets in a room with restricted access) and held for a minimum of 25 years after the end of the sub-study.

19.10.2 ETHICAL CONDUCT

Ethical Considerations

There are no new ethical considerations for the sub-study which are not among those described for the main COVAC1 in Section 10.2.1.

Favourable Ethical Opinion, HRA Approval, and Host Site Permission

The processes for the sub-study will be the same as for the main COVAC1 trial – see Section 10.2.2.

19.10.3 COMPETENT AUTHORITY APPROVALS

Not required for this sub-study.

19.10.4 SUB-STUDY CLOSURE

The end of the sub-study coincides with the end of the main COVAC1 trial, as defined in **Section 6.11**.

19.11 INDEMNITY

Imperial College London holds negligent harm and non-negligent harm insurance policies which apply to this sub-study.

19.12 FINANCE

Tamara Elliott is supported by a Wellcome clinical PhD fellowship at Imperial College London.

19.13 OVERSIGHT & TRIAL COMMITTEES

Oversight of the sub-study will be delegated by the sponsor to the CI. Oversight of the sub-study is not within the scope of MRC CTU's responsibilities, or those of the TMT, TMG or TSC. Nevertheless the MRC CTU will be kept informed of the sub-study by being sent copies of all documents before submission for approval, all approved documents, and will be kept updated during TMT and TMG meetings.

The MRC CTU will place sub-study documentation within the TMF.

19.13.1 Role of Study Sponsor

Imperial College London, as represented by the Imperial Joint Clinical Research Office, is the sponsor of the trial and maintains overall responsibility for the conduct and reporting of the entire COVAC1 trial, including the sub-study.

19.14 PATIENT AND PUBLIC INVOLVEMENT

No Patient and Public Involvement (PPI) was sought for this small add-on to the main COVAC1 trial.

19.15 PUBLICATION AND DISSEMINATION OF RESULTS

The sub-study results will form part of an Imperial College London PhD thesis. If the data merit, the preparation of a manuscript for publication in a peer-reviewed professional journal or an abstract for presentation, oral or written, to a learned society or symposium will be discussed by the PhD candidate and supervisors.

Authorship will reflect work done by the investigators and other personnel involved in the analysis and interpretation of the data, in accordance with generally recognised principles of scientific collaboration.

19.16 DATA AND/OR SAMPLE SHARING

Data may be shared by ICL, based on the following principles:

- No data should be released that would compromise an ongoing trial or study.
- There must be a strong scientific or other legitimate rationale for the data to be used for the requested purpose.
- Investigators who have invested time and effort into developing the study should have a period of exclusivity in which to pursue their aims with the data, before key study data are made available to other researchers.
- Data exchange and processing must comply with Information Governance and Data Security Policies in all of the relevant territories.

Researchers wishing to access sub-study data should contact the CI in the first instance.

19.17 REFERENCES

1. Iwasaki A. What reinfections mean for COVID-19. Lancet Infect Dis. 2020 Oct;0(0).
2. Tillett RL, Sevinsky JR, Hartley PD, Kerwin H, Crawford N, Gorzalski A, et al. Genomic evidence for reinfection with SARS-CoV-2: a case study. Lancet Infect Dis. 2020 Oct 12;0(0).
3. To KK-W, Hung IF-N, Ip JD, Chu AW-H, Chan W-M, Tam AR, et al. Coronavirus Disease 2019 (COVID-19) Re-infection by a Phylogenetically Distinct Severe Acute Respiratory Syndrome Coronavirus 2 Strain Confirmed by Whole Genome Sequencing. Clin Infect Dis. 2020 Aug 25;
4. Van Elslande J, Vermeersch P, Vandervoort K, Wawina-Bokalanga T, Vanmechelen B, Wollants E, et al. Symptomatic SARS-CoV-2 reinfection by a phylogenetically distinct strain. Clin Infect Dis. 2020 Sep 5;
5. Prado-Vivar B, Becerra-Wong M, Guadalupe JJ, Marquez S, Gutierrez B, Rojas-Silva P, et al. COVID-19 Re-Infection by a Phylogenetically Distinct SARS-CoV-2 Variant, First Confirmed Event in South America. SSRN Electron J. 2020 Sep 3;
